# Supplementary material for: GCN sensitive protein translation in yeast
Source: PLoS One. 2020 Sep 18;15(9):e0233197. doi: 10.1371/journal.pone.0233197 (PMC7500604; doi:10.1371/journal.pone.0233197)
Supplement: S1 Fig — Histogram and density plot of the protein expression levels for all yeast genes, as measured by [33]. Protein expression levels for SKN7 and HMT1 are indicated. (PDF) [file pone.0233197.s001.pdf]

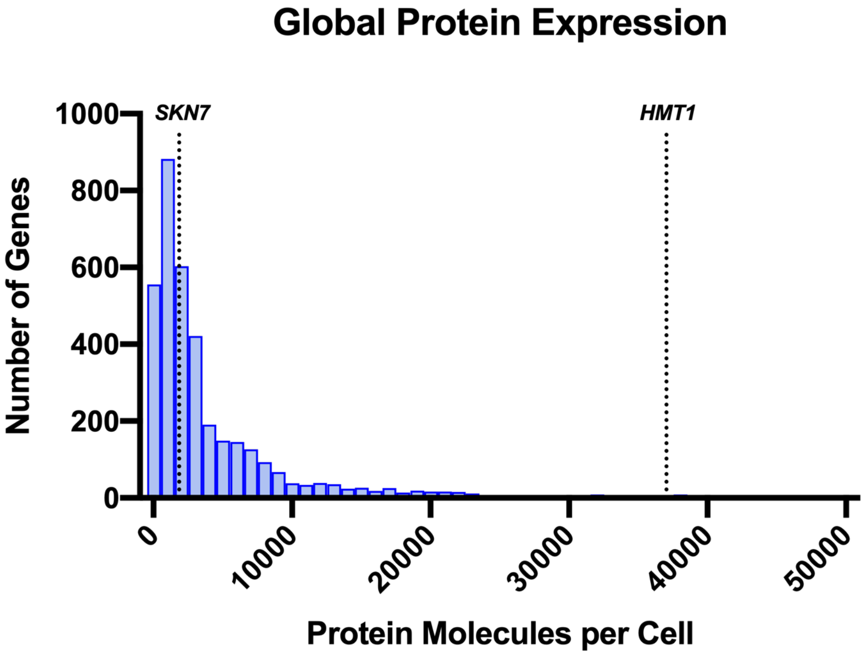

S1 Fig. Global protein expression. Histogram and density plot of the protein expression levels for all yeast genes, as measured by Ghaemmaghami *et al*. (2003). Protein expression levels for SKN7 and HMT1 are indicated.
