# Supplementary material for: GCN sensitive protein translation in yeast
Source: PLoS One. 2020 Sep 18;15(9):e0233197. doi: 10.1371/journal.pone.0233197 (PMC7500604; doi:10.1371/journal.pone.0233197)

Raw Data for Fig 4B: Western images used for steady state protein expression quantitation

All blots were visualized using the GE Amersham ECL Prime Western Blotting Detection Reagent (Fisher Scientific) and imaged in a Syngene G:Box. SynGene software was used to verify that signals did not exceed the linear dynamic range of detection. Pixel density was quantified for each sample using ImageJ. Prior to quantitation, images were converted to a 16-bit format and background signal was subtracted using a sliding-paraboloid algorithm.
Pixel density was plotted for each lane and the area under the curve was calculated as a proxy measurement for protein abundance. Blots were loaded left to right.

SKN7::GCNpm Western (set 1)
Primary: Anti-TAP
Secondary: Anti-rabbit HRP-conjugated

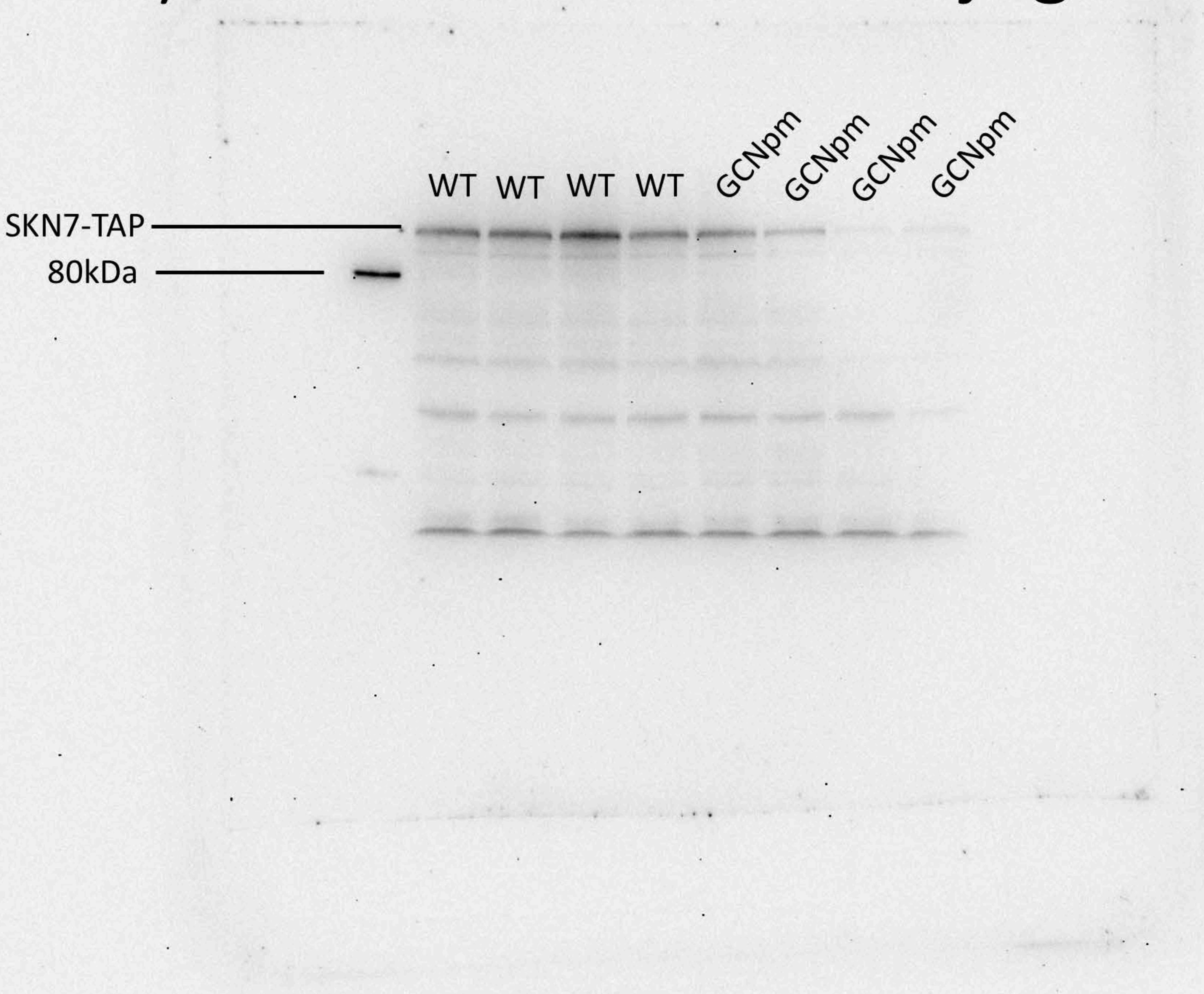

SKN7::GCNpm Western (set 1)
Primary: Anti-Tub1p
Secondary: Anti-rabbit HRP-conjugated

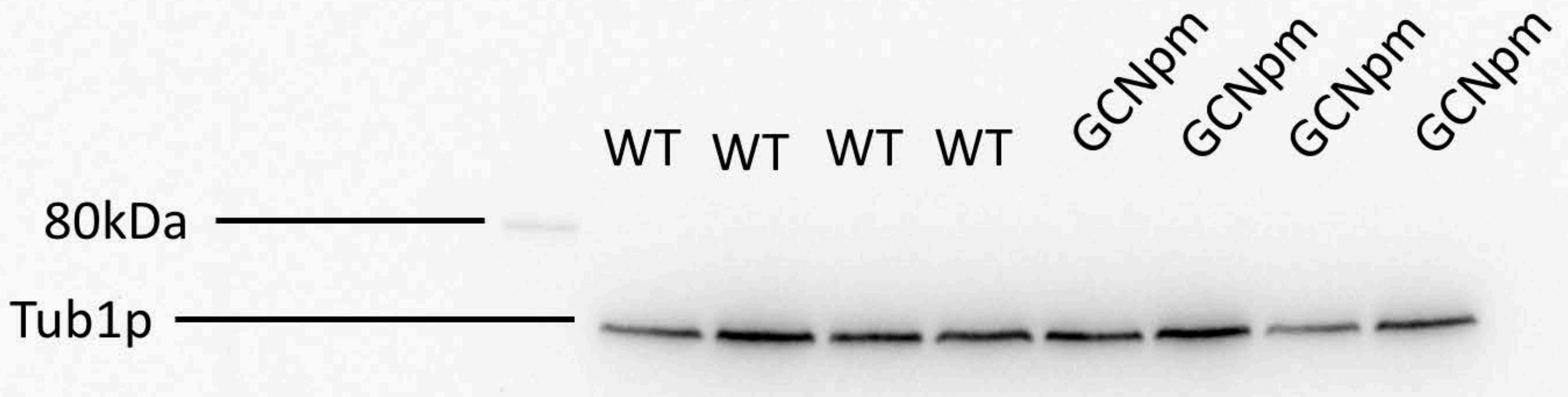

SKN7::GCNpm Western (set 2)
Primary: Anti-TAP
Secondary: Anti-rabbit HRP-conjugated

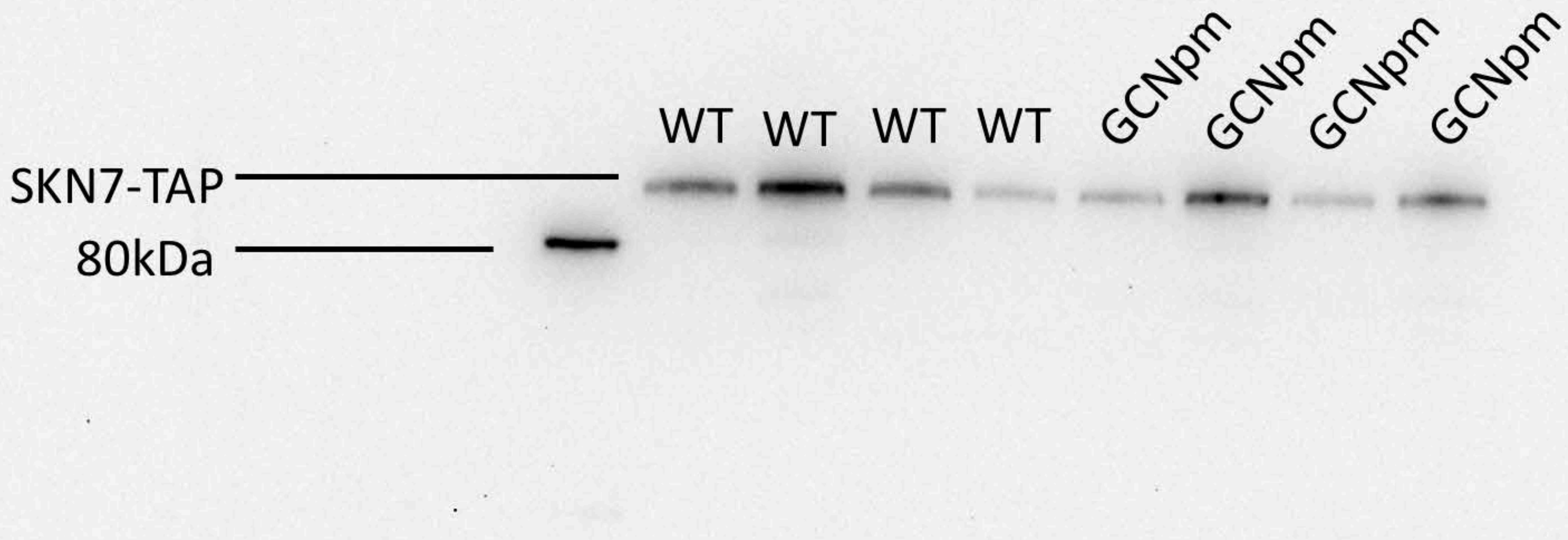

SKN7::GCNpm Western (set 2)
Primary: Anti-Tub1p
Secondary: Anti-rabbit HRP-conjugated

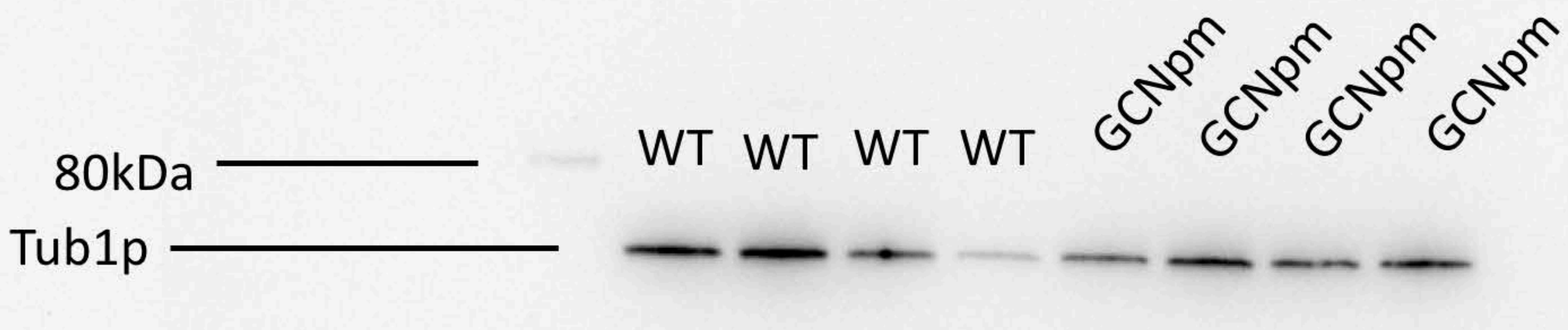

SKN7::G2 Western (set 1)
Primary: Anti-TAP
Secondary: Anti-rabbit HRP-conjugated

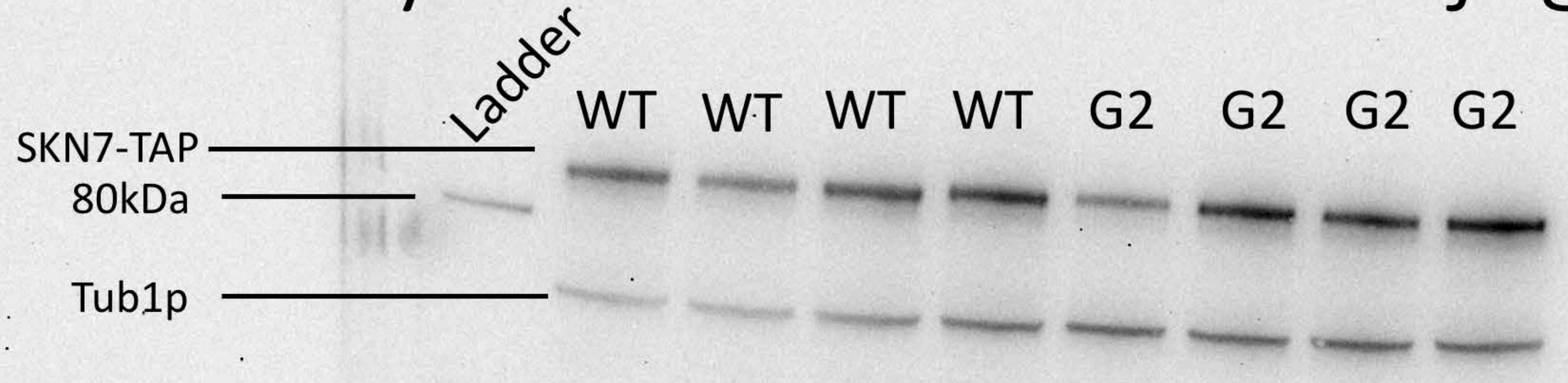

SKN7::G2 Western (set 1)
Primary: Anti-Tub1p
Secondary: Anti-rabbit HRP-conjugated

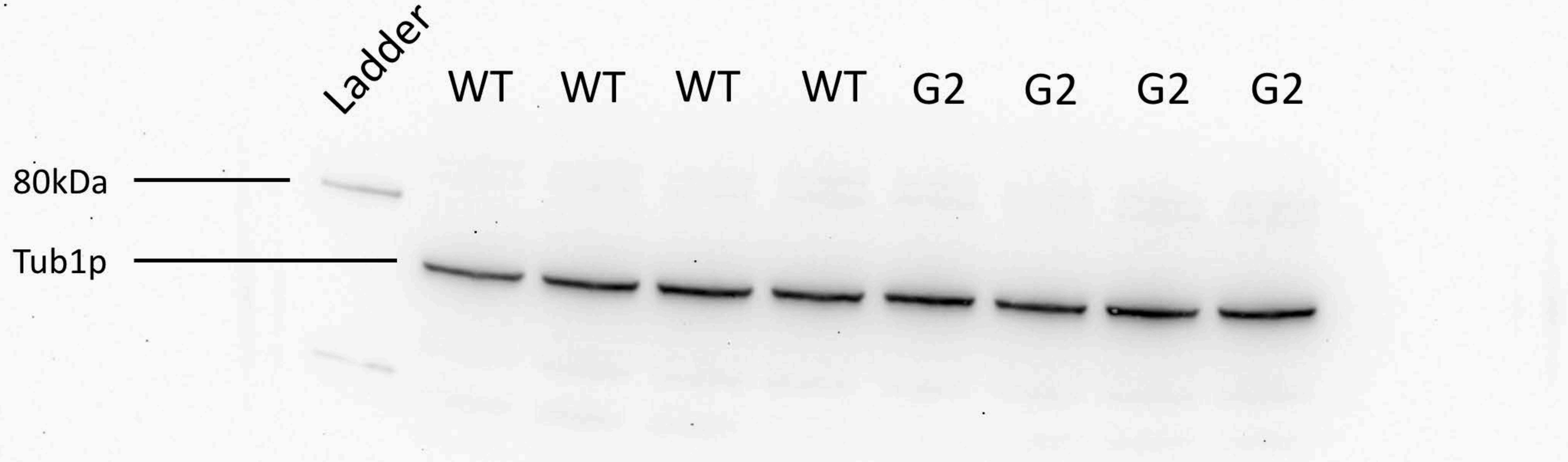

SKN7::G2 Western (set 2)
Primary: Anti-TAP
Secondary: Anti-rabbit HRP-conjugated

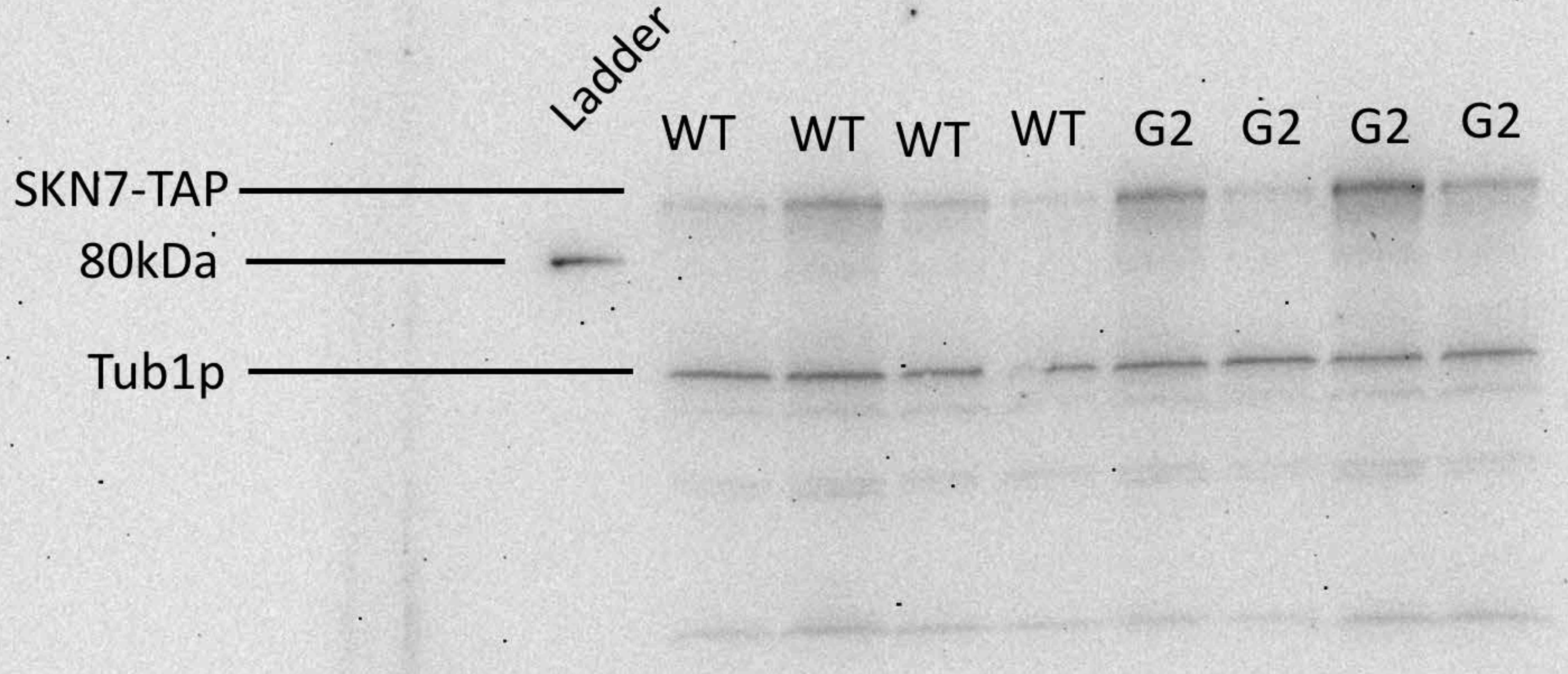

SKN7::G2 Western (set 2)
Primary: Anti-Tub1p
Secondary: Anti-rabbit HRP-conjugated

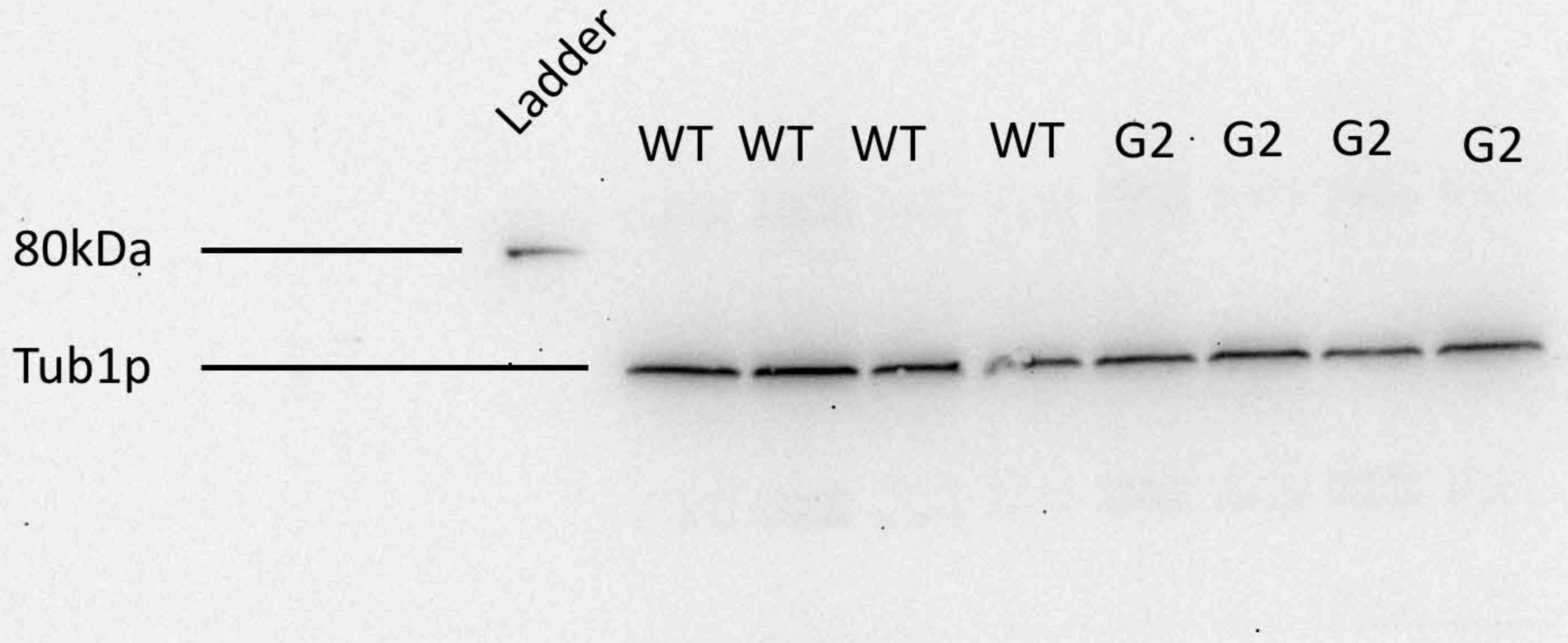

SKN7::A-rich Western (set 1)
Primary: Anti-TAP
Secondary: Anti-rabbit HRP-conjugated

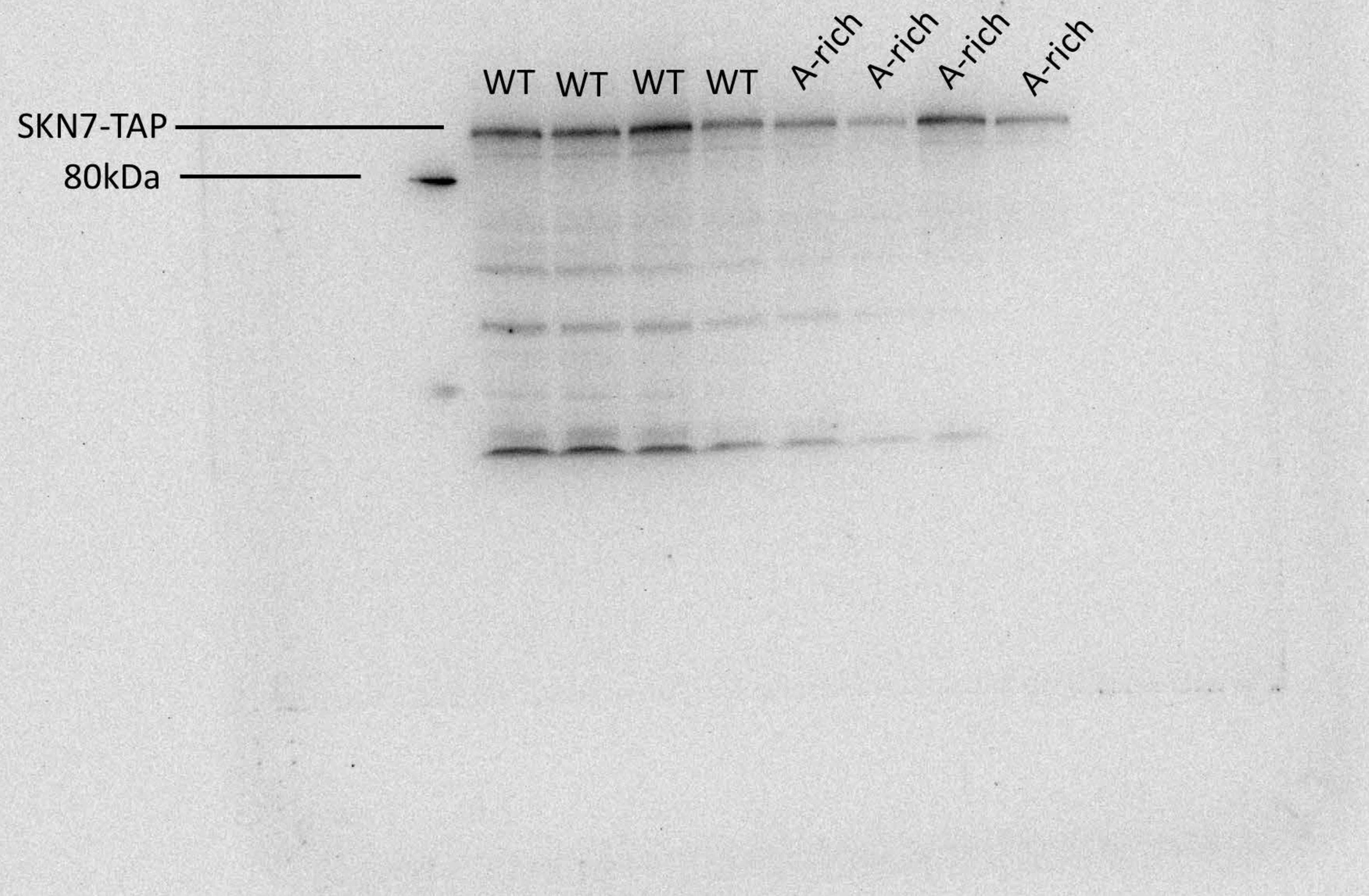

SKN7::A-rich Western (set 1)
Primary: Anti-Tub1p
Secondary: Anti-rabbit HRP-conjugated

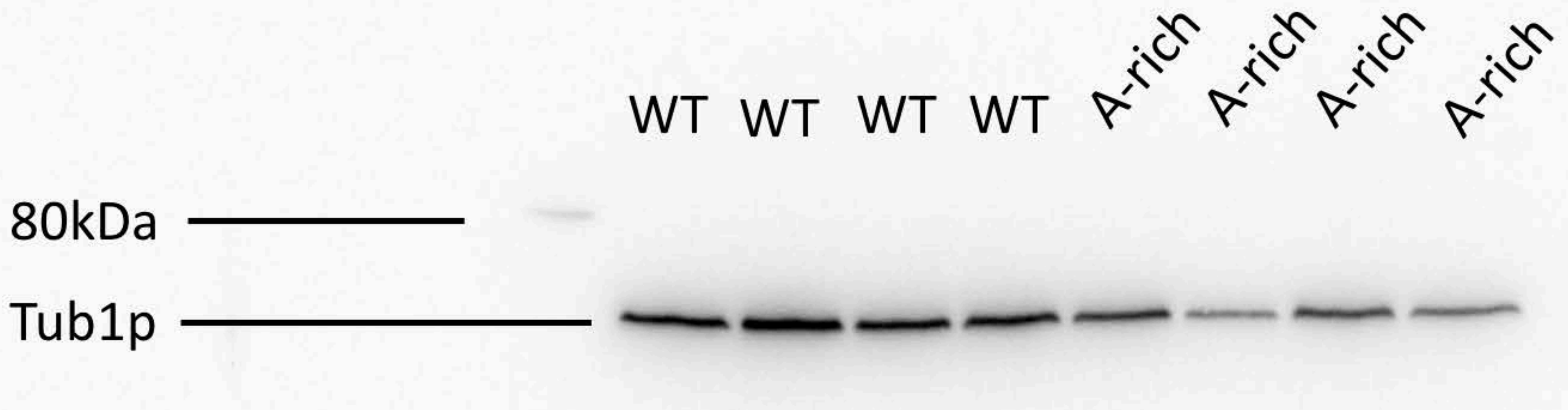

SKN7::A-rich Western (set 2)
Primary: Anti-TAP
Secondary: Anti-rabbit HRP-conjugated

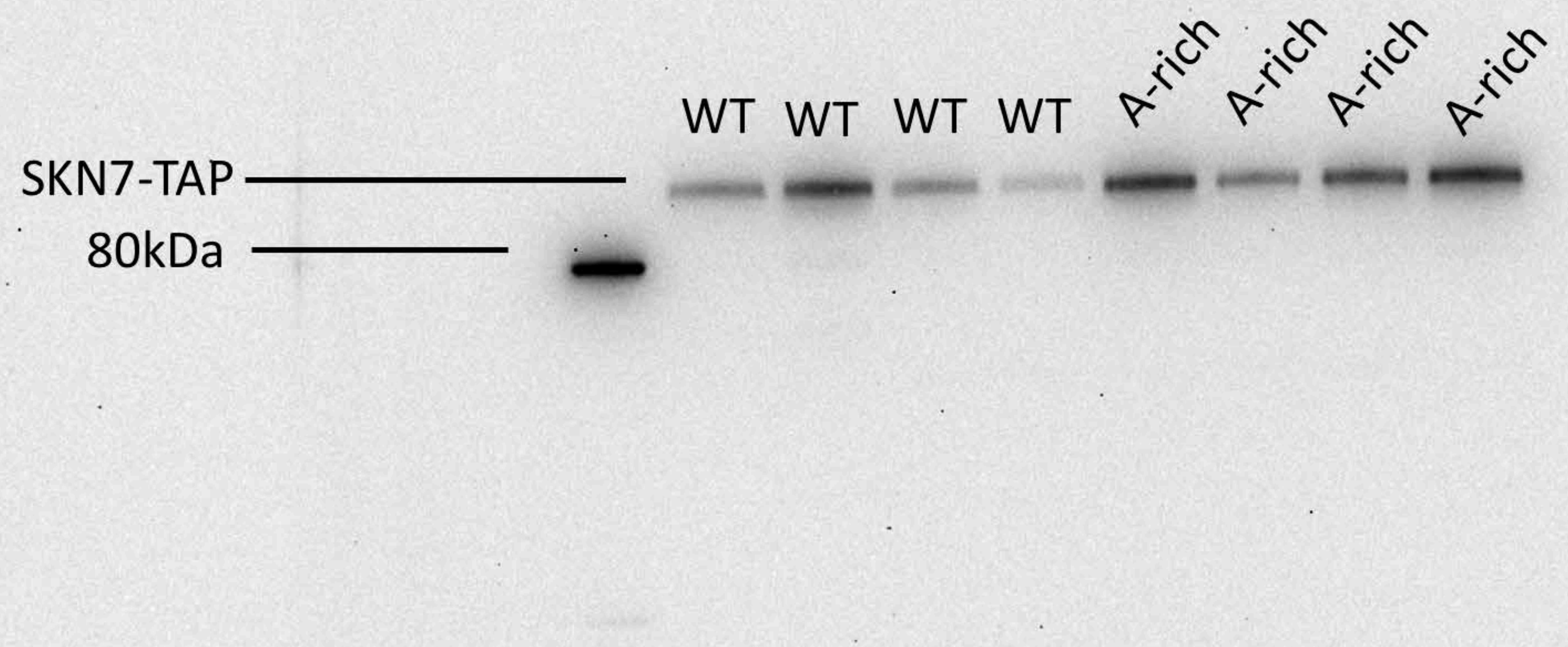

SKN7::A-rich Western (set 2)
Primary: Anti-Tub1p
Secondary: Anti-rabbit HRP-conjugated

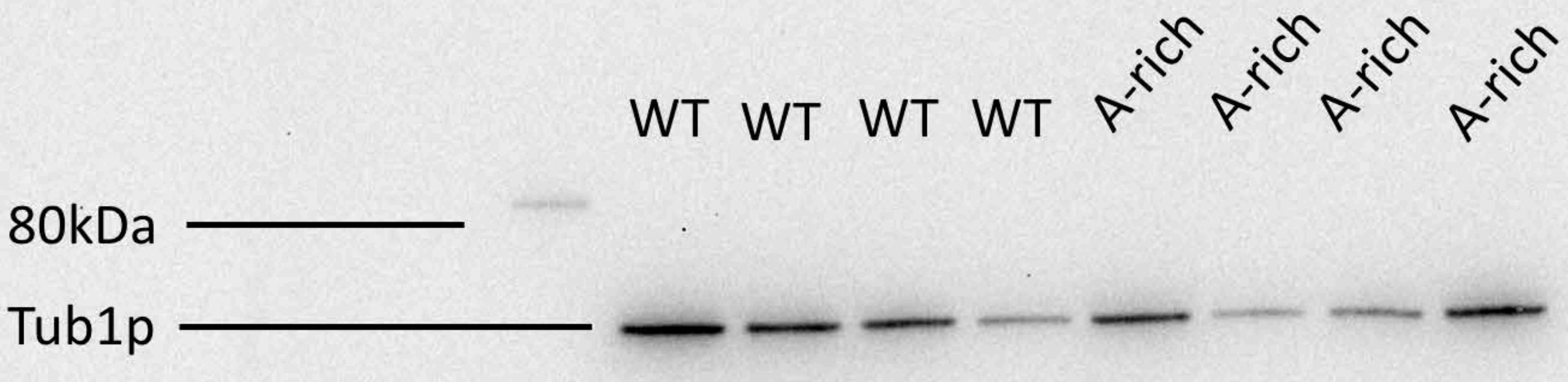

SKN7::GCNi Western (set 1)
Primary: Anti-TAP
Secondary: Anti-rabbit HRP-conjugated

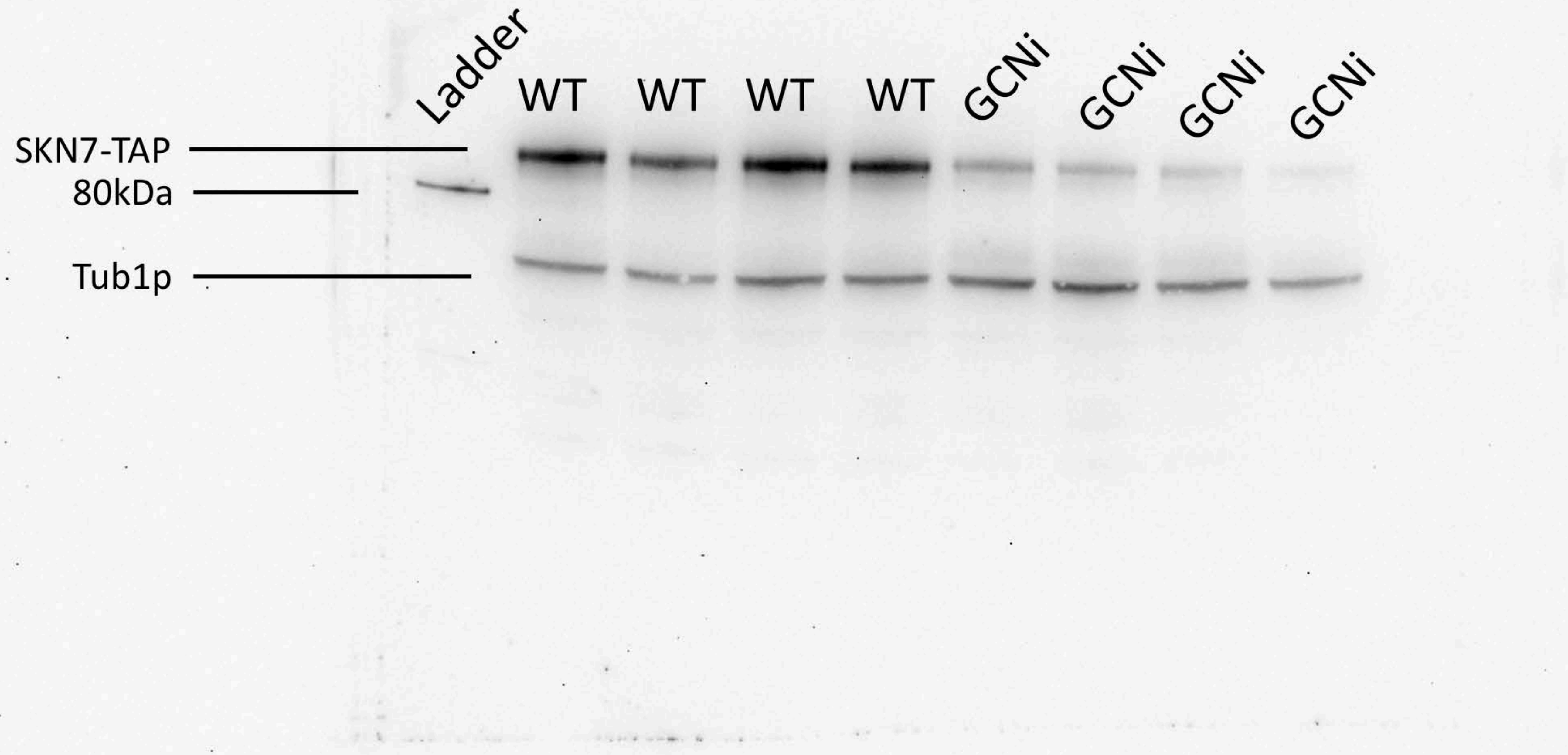

SKN7::GCNi Western (set 1)
Primary: Anti-Tub1p
Secondary: Anti-rabbit HRP-conjugated

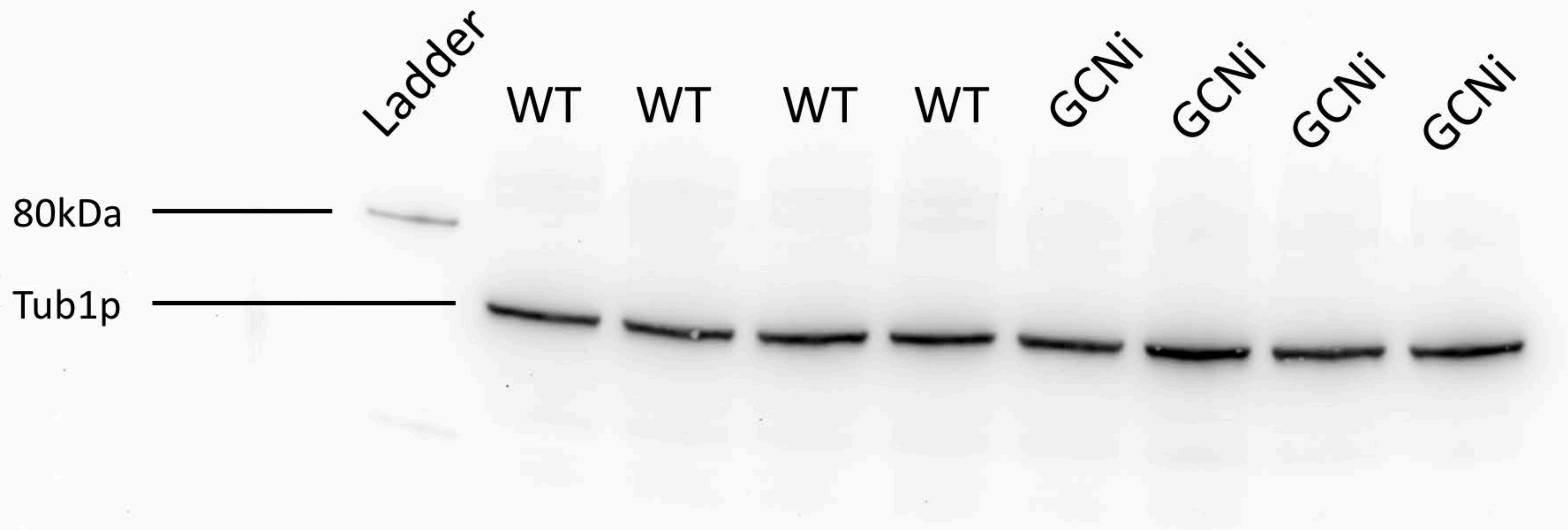

SKN7::GCNi Western (set 2)
Primary: Anti-TAP
Secondary: Anti-rabbit HRP-conjugated

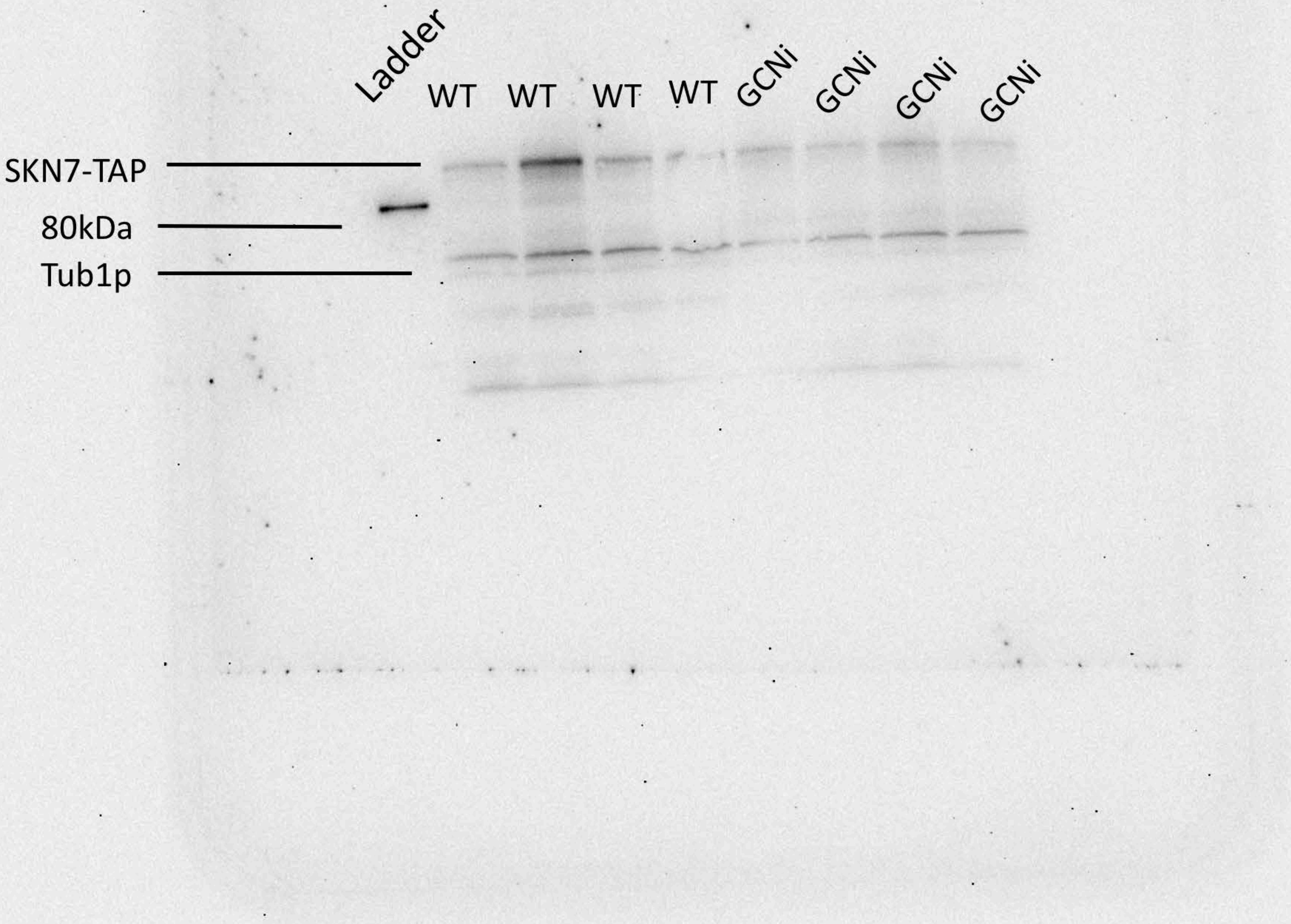

SKN7::GCNi Western (set 2)
Primary: Anti-Tub1p
Secondary: Anti-rabbit HRP-conjugated

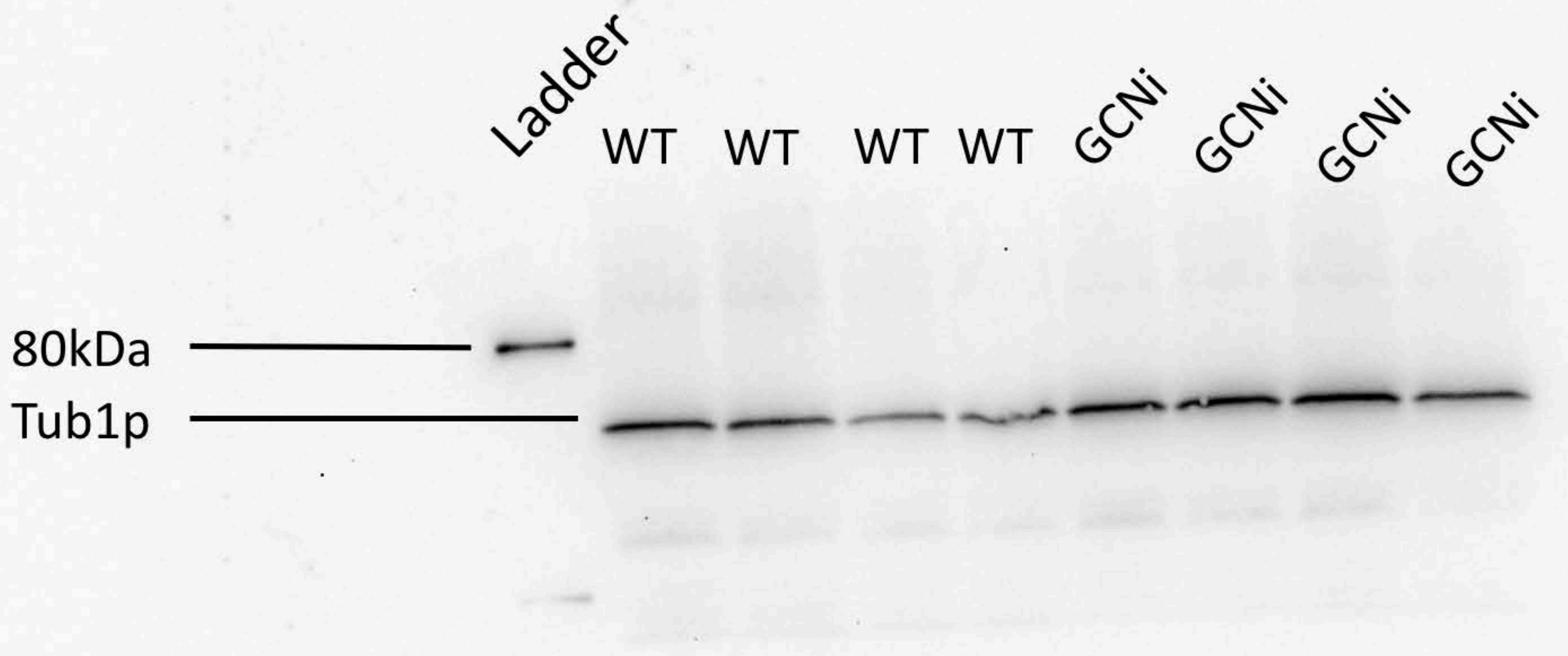

HMT1::GCNpm Western (set 1)
Primary: Anti-TAP
Secondary: Anti-rabbit HRP-conjugated

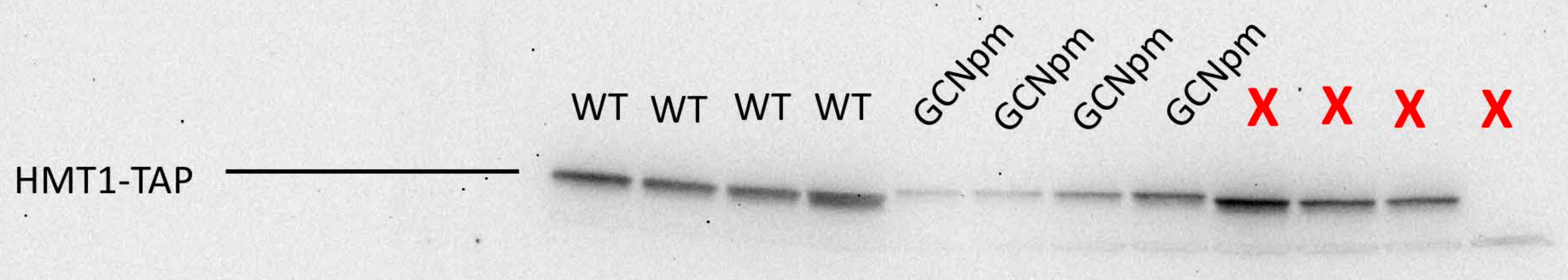

HMT1::GCNpm Western (set 1)
Primary: Anti-Tub1p
Secondary: Anti-rabbit HRP-conjugated

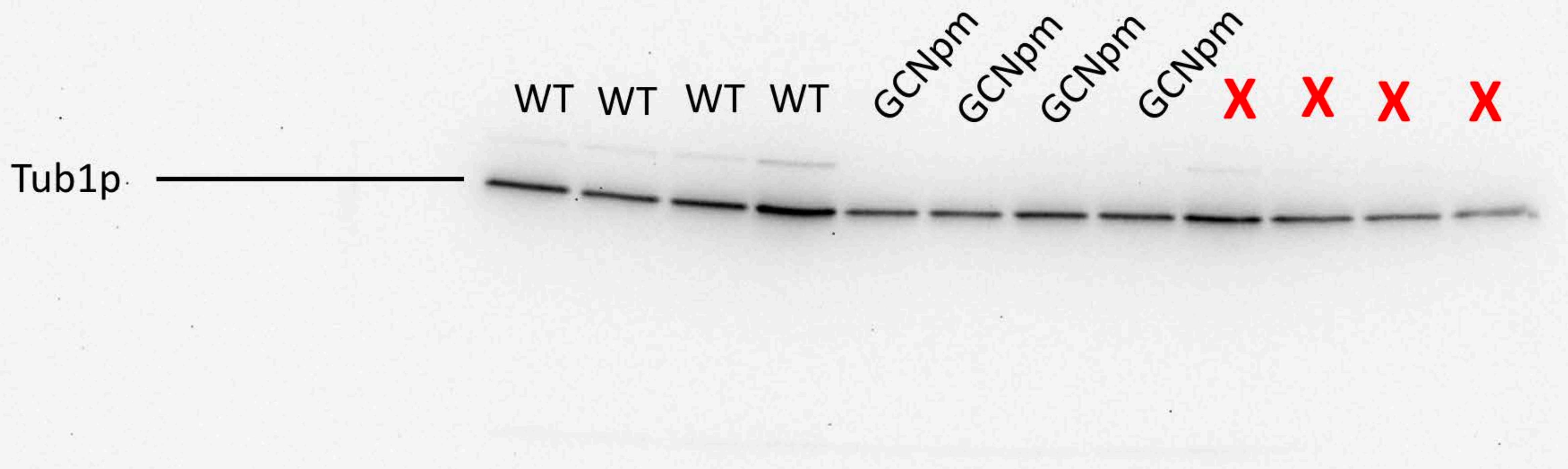

HMT1::GCNpm Western (set 2)
Primary: Anti-TAP
Secondary: Anti-rabbit HRP-conjugated

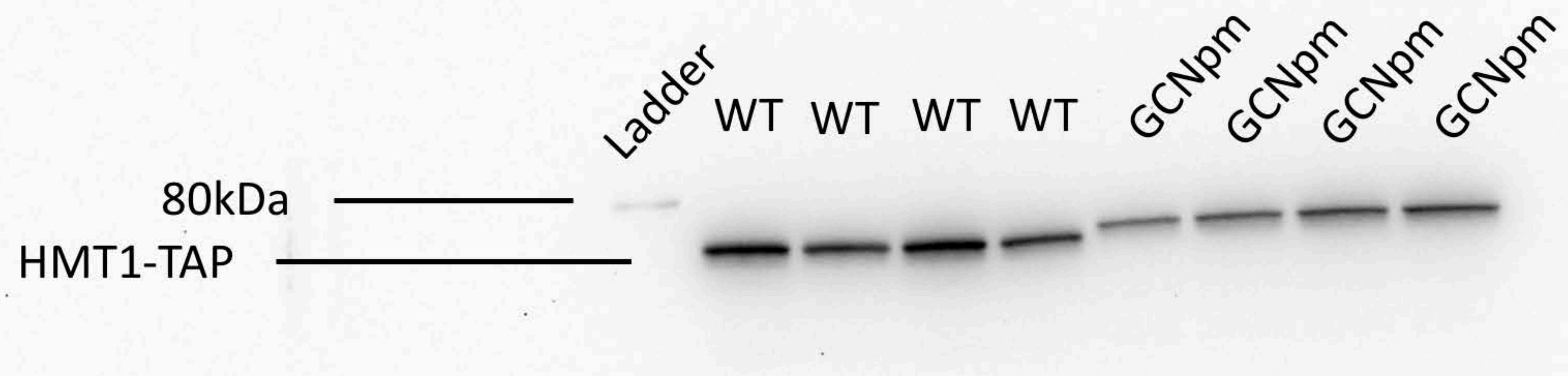

HMT1::GCNpm Western (set 2)
Primary: Anti-Tub1p
Secondary: Anti-rabbit HRP-conjugated

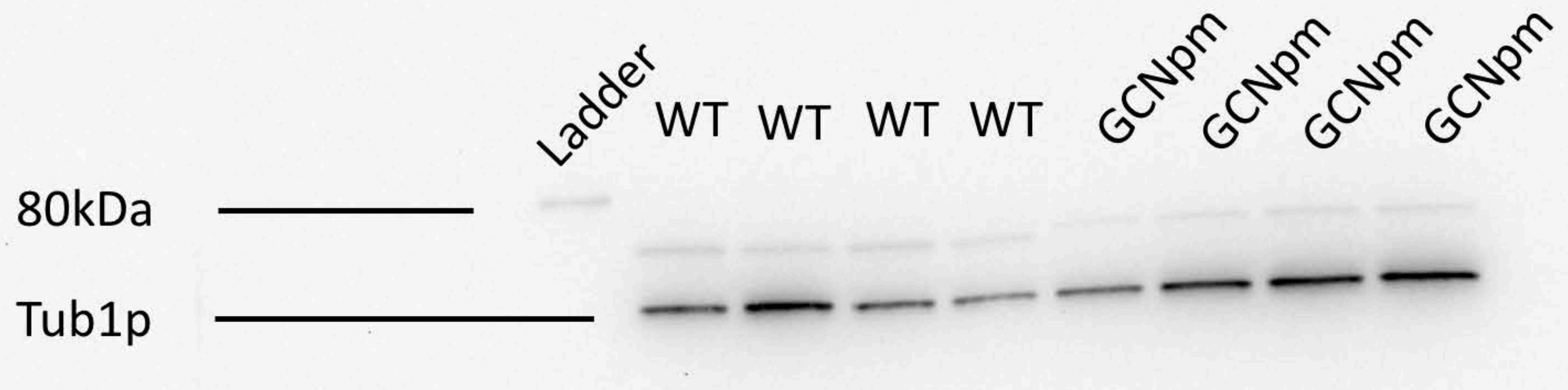

HMT1::G2 Western (set 1)
Primary: Anti-TAP
Secondary: Anti-rabbit HRP-conjugated

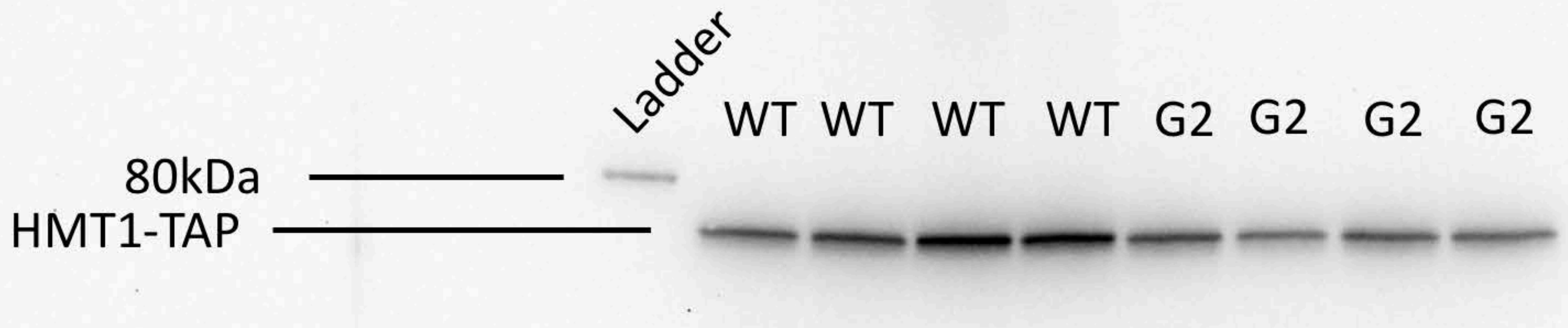

HMT1::G2 Western (set 1)
Primary: Anti-Tub1p
Secondary: Anti-rabbit HRP-conjugated

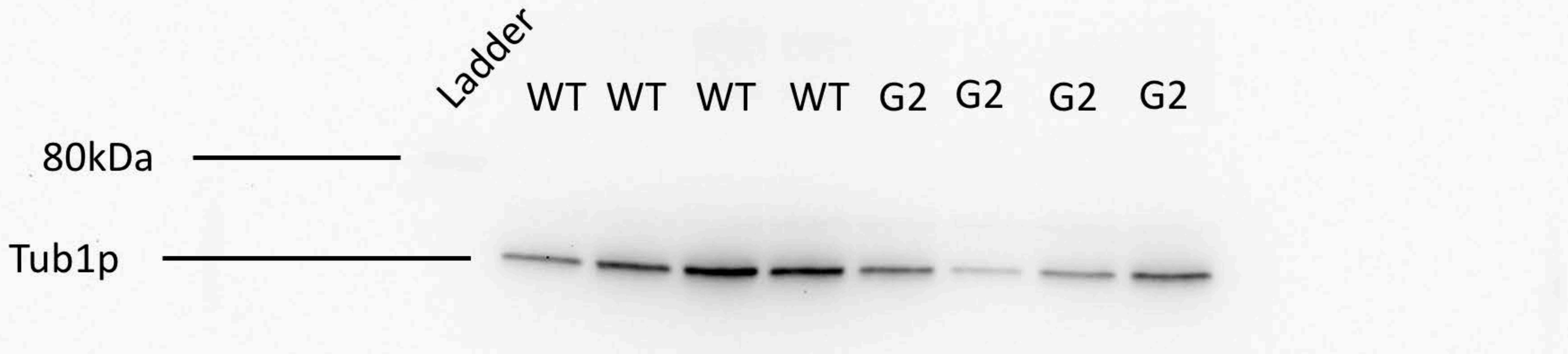

HMT1::C1, HMT1::G2 Western (set 2)
Primary: Anti-TAP
Secondary: Anti-rabbit HRP-conjugated

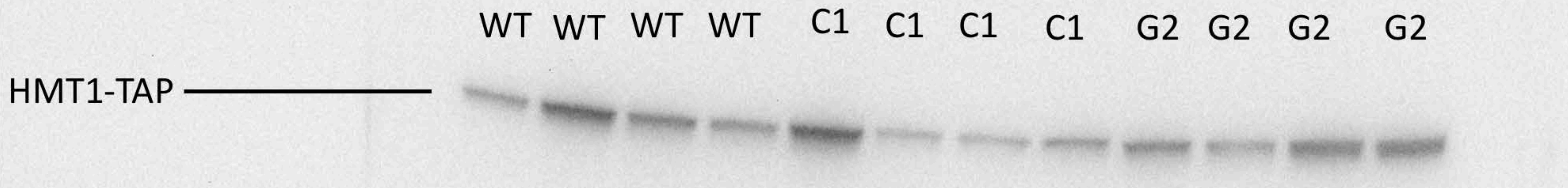

HMT1::C1, HMT1::G2 Western (set 2)
Primary: Anti-Tub1p
Secondary: Anti-rabbit HRP-conjugated

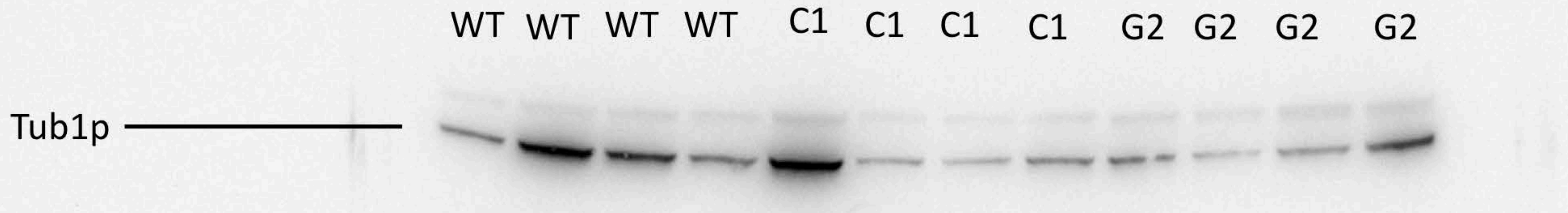

HMT1::C1 Western (set 1)
Primary: Anti-TAP
Secondary: Anti-rabbit HRP-conjugated

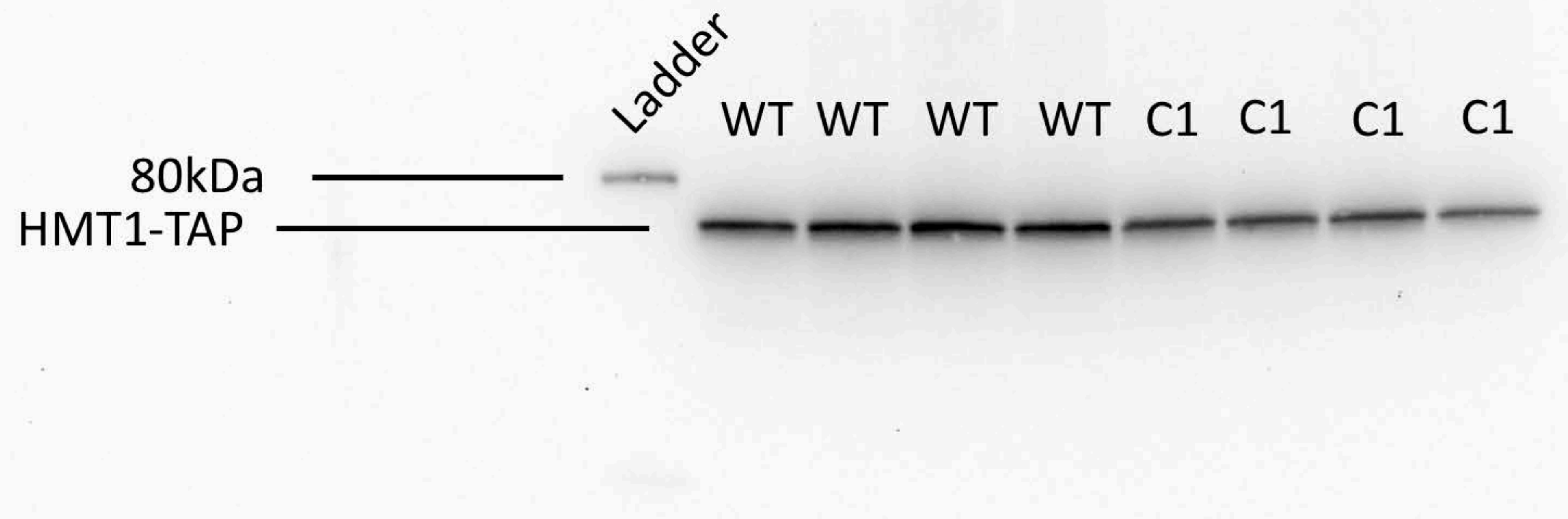

HMT1::C1 Western (set 1)
Primary: Anti-Tub1p
Secondary: Anti-rabbit HRP-conjugated

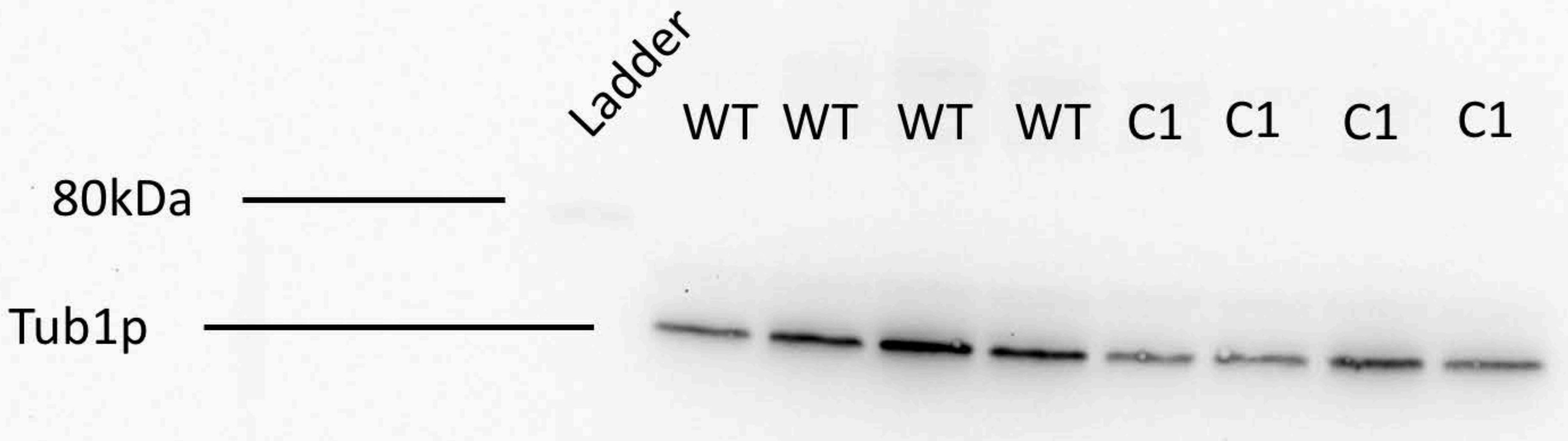

Supplement: S2 Fig — Western images used in quantitation described in Fig 4. These images have exposure times ranging from ten seconds to five minutes. The images within the linear dynamic range of detection with the least background signal were used in densitometric analyses. The images are separated by mutant strain. Each lane represents an independently-grown culture for that strain. TAP and Tub1 (α-tubulin) images are paired for each gel. Note that in some experiments, incomplete stripping after the first probing led to some retention of band signals. Also, with the anti-tubulin incubations, the TAP protein can also be visible because the secondary antibody has an IgG domain that recognizes the Protein A region of the TAP epitope tag; this was most pronounced for the more abundant HMT1 protein. (PDF) [file pone.0233197.s002.pdf]
