# Supplementary material for: GCN sensitive protein translation in yeast
Source: PLoS One. 2020 Sep 18;15(9):e0233197. doi: 10.1371/journal.pone.0233197 (PMC7500604; doi:10.1371/journal.pone.0233197)

Raw Data for Fig 5C: Cycloheximide chase western images used for quantitation

All blots were visualized using the GE Amersham ECL Prime Western Blotting Detection Reagent (Fisher Scientific) and imaged in a Syngene G:Box. SynGene software was used to verify that signals did not exceed the linear dynamic range of detection. Pixel density was quantified for each sample using ImageJ. Prior to quantitation, images were converted to a 16-bit format and background signal was subtracted using a sliding-paraboloid algorithm.
Pixel density was plotted for each lane and the area under the curve was calculated as a proxy measurement for protein abundance. Blots were loaded left to right.

SKN7 WT compared to SKN7::GCNpm

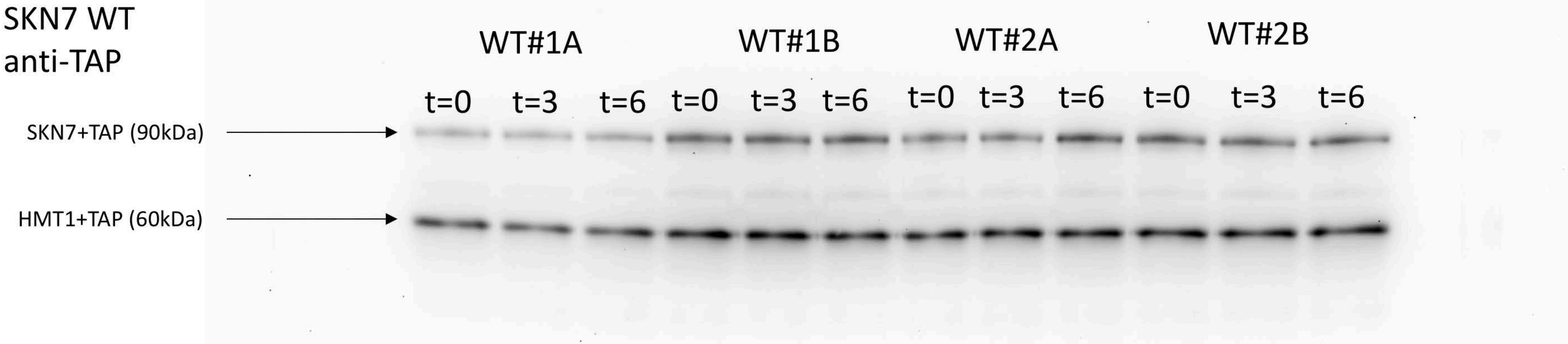

SKN7 WT compared to SKN7::GCNpm

SKN7::GCNpm (G7)
anti-TAP

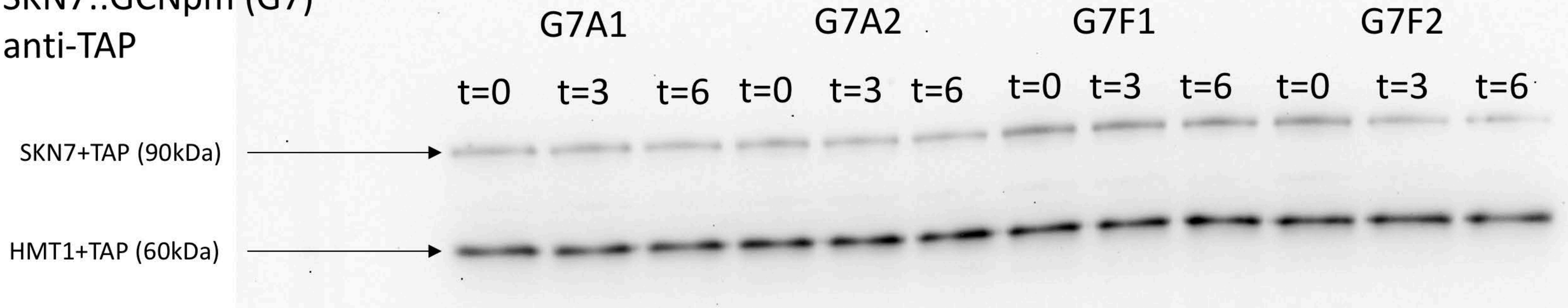

SKN7 WT compared to SKN7::G2

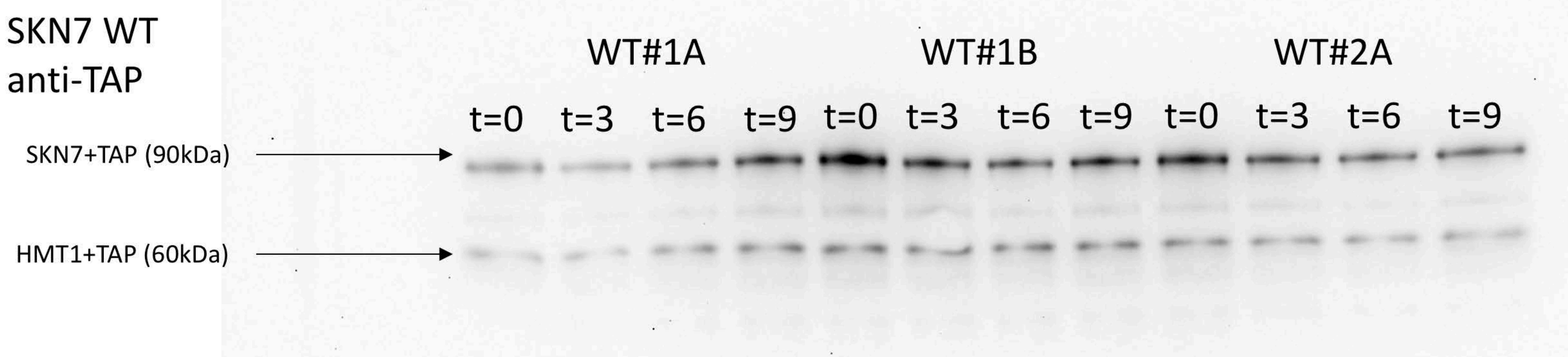

SKN7 WT compared to SKN7::G2

SKN7::G2
anti-TAP

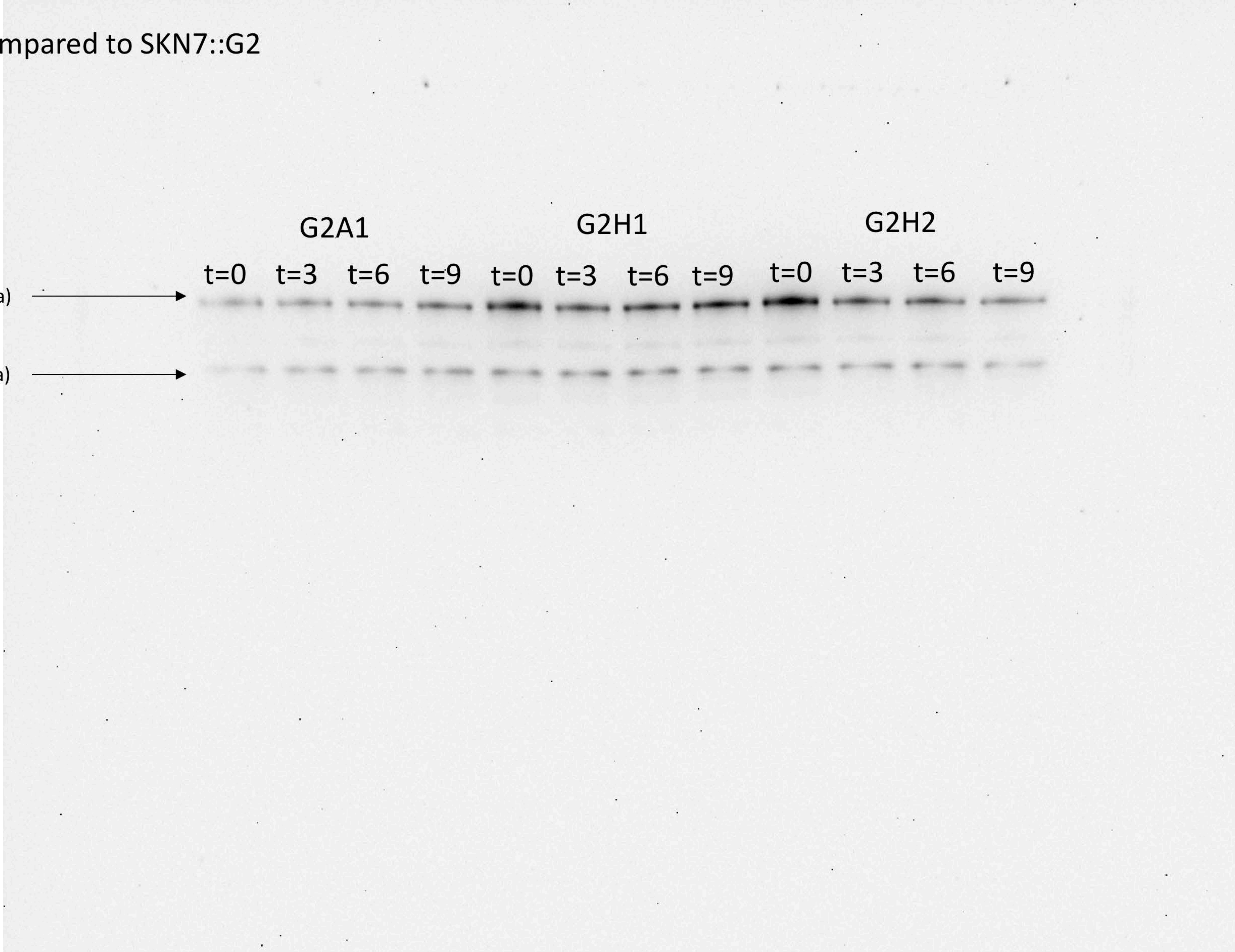

SKN7 WT compared to SKN7::A-rich

SKN7 WT
anti-TAP

SKN7+TAP (90kDa)

HMT1+TAP (60kDa)

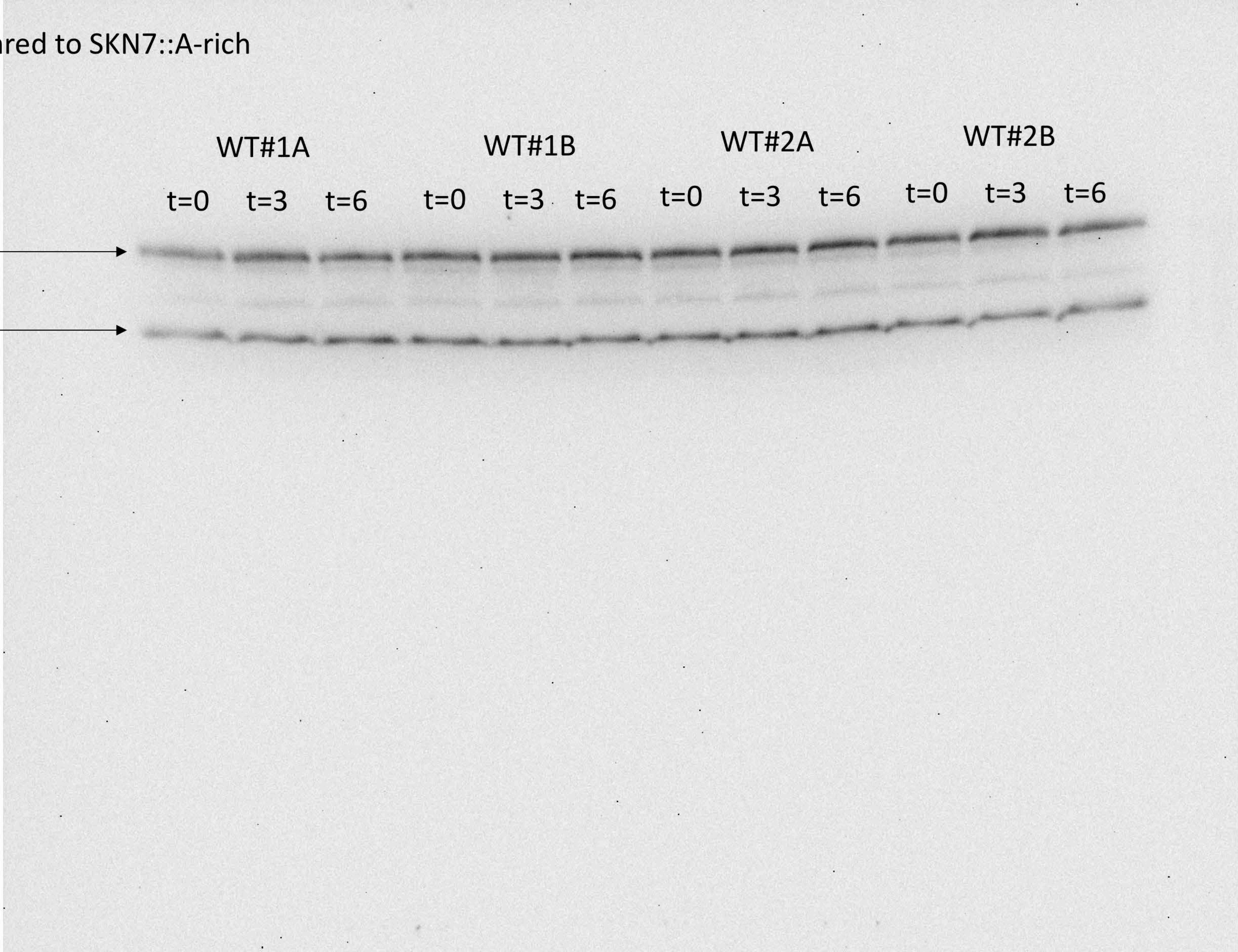

SKN7 WT compared to SKN7::A-rich

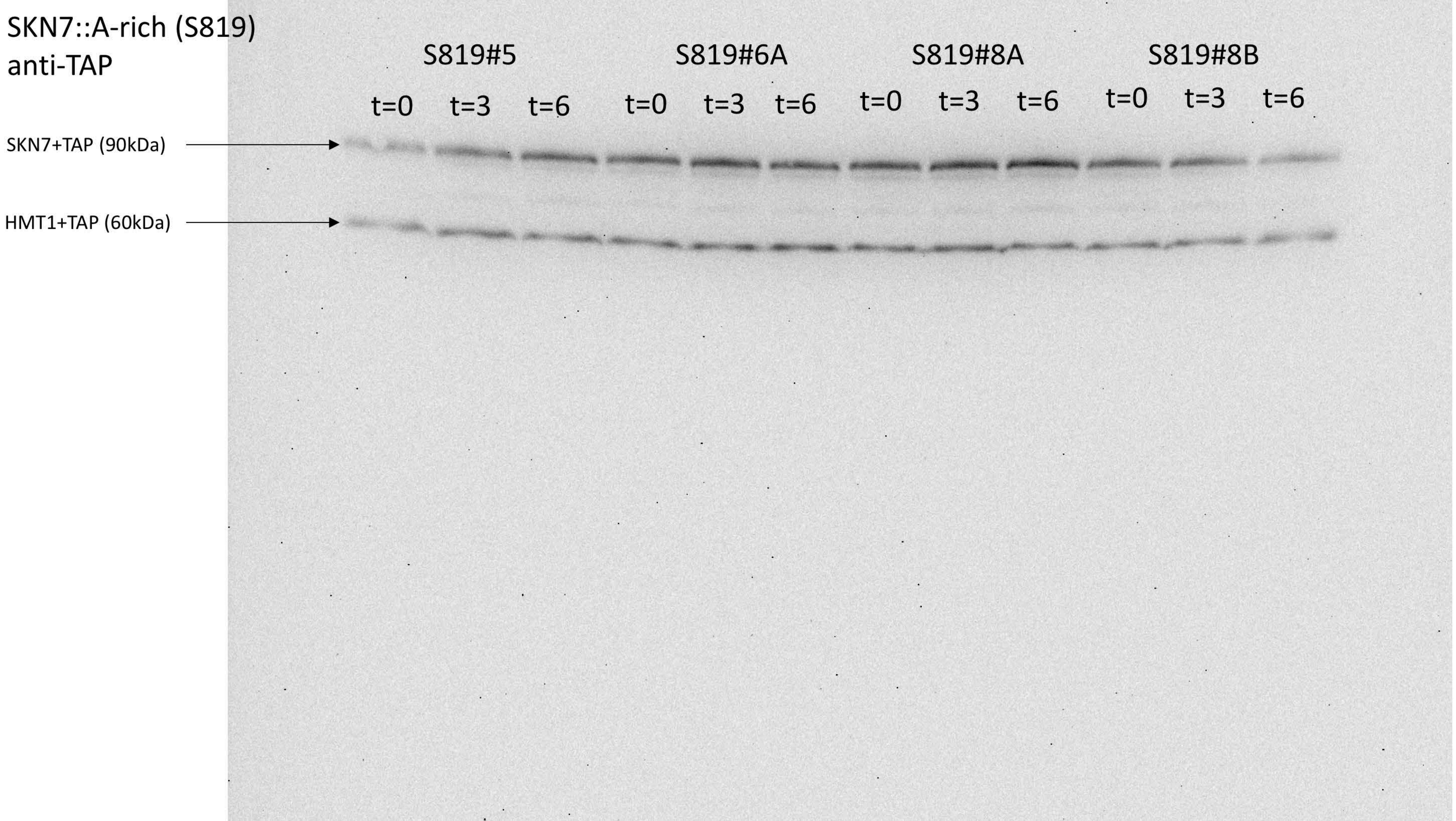

SKN7 WT compared to SKN7::GCNi

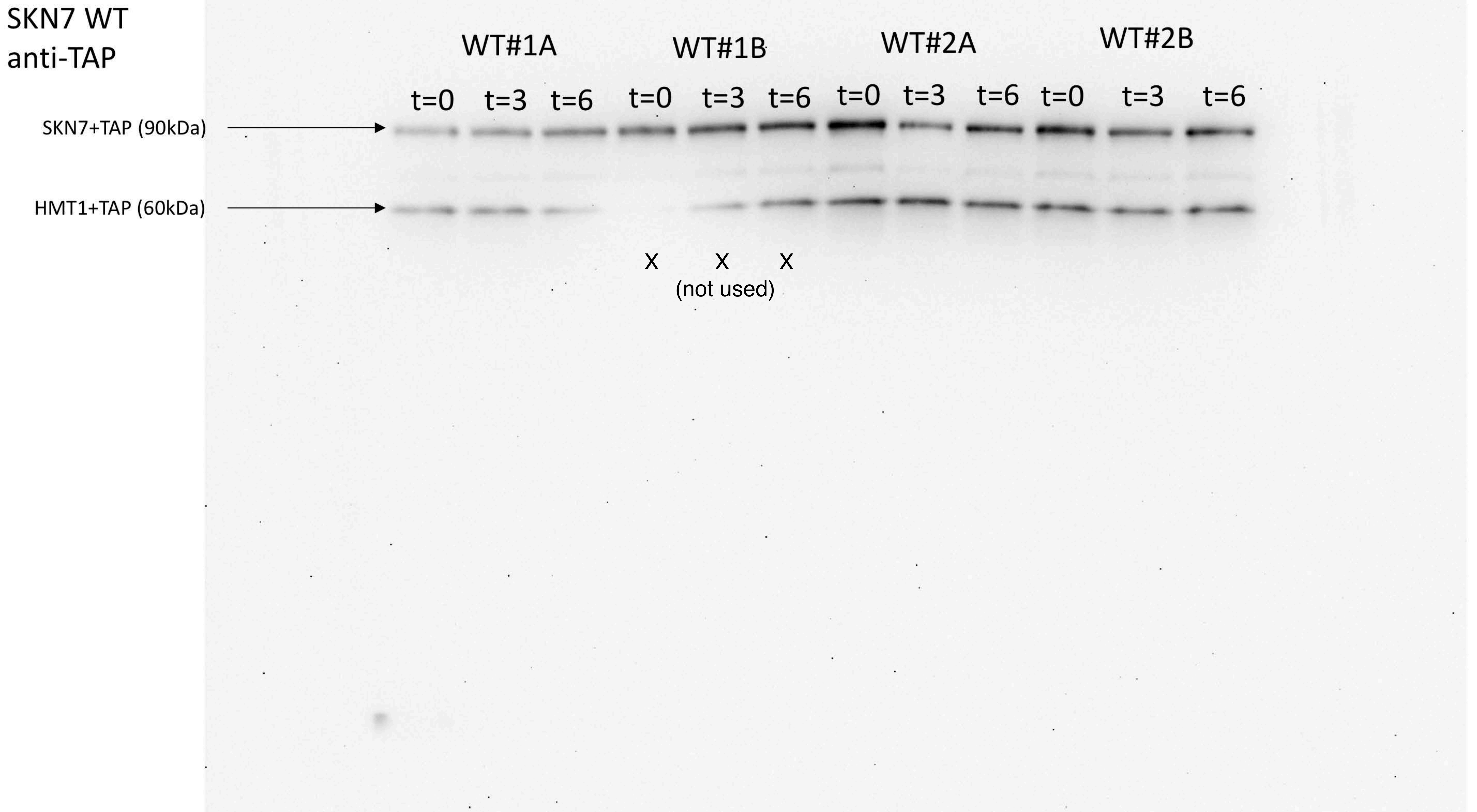

SKN7 WT compared to SKN7::GCNi

SKN7::GCNi (I7)
anti-TAP

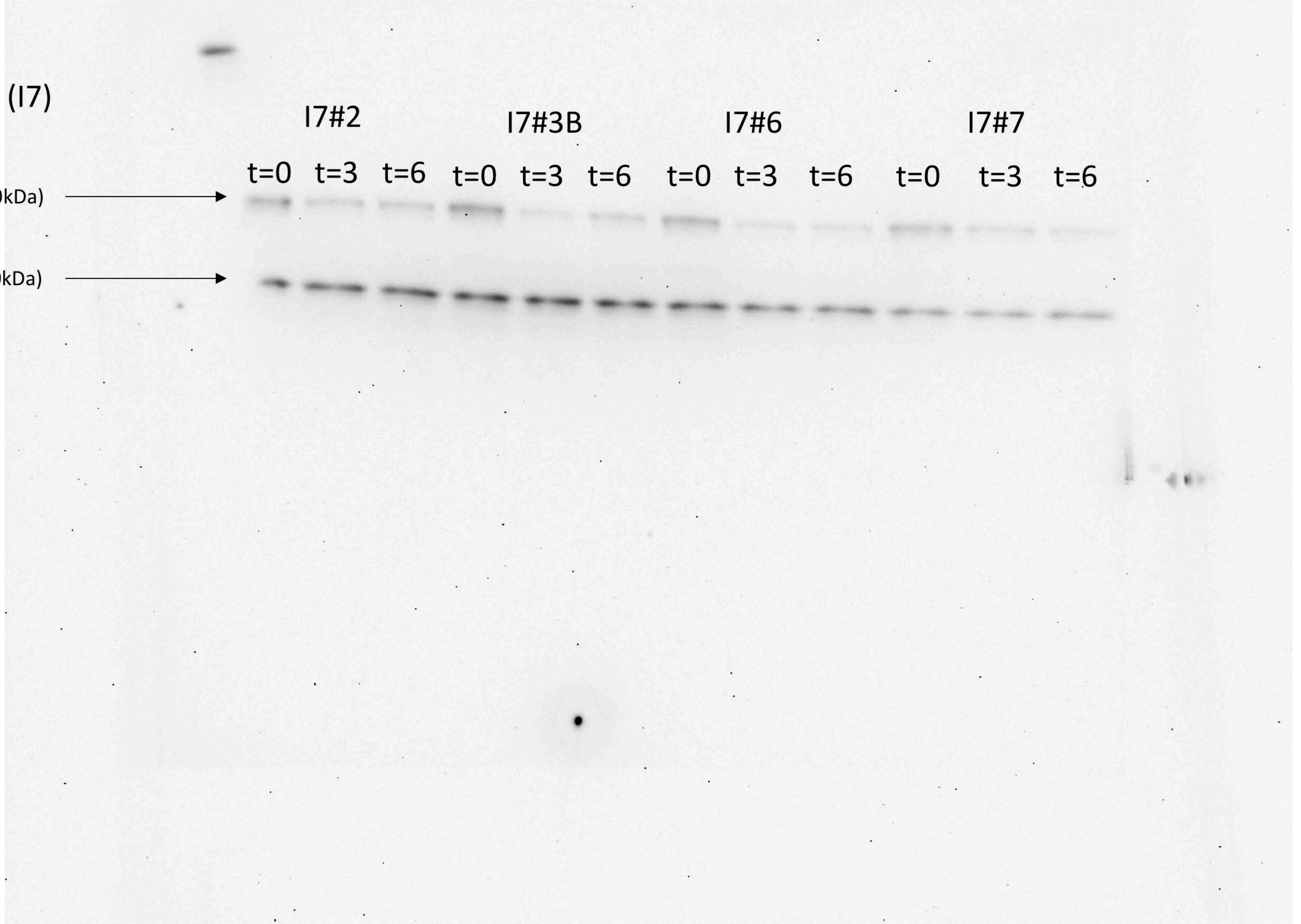

SKN7 WT compared to SKN7::GCNi
(set 2)

SKN7 WT
anti-TAP

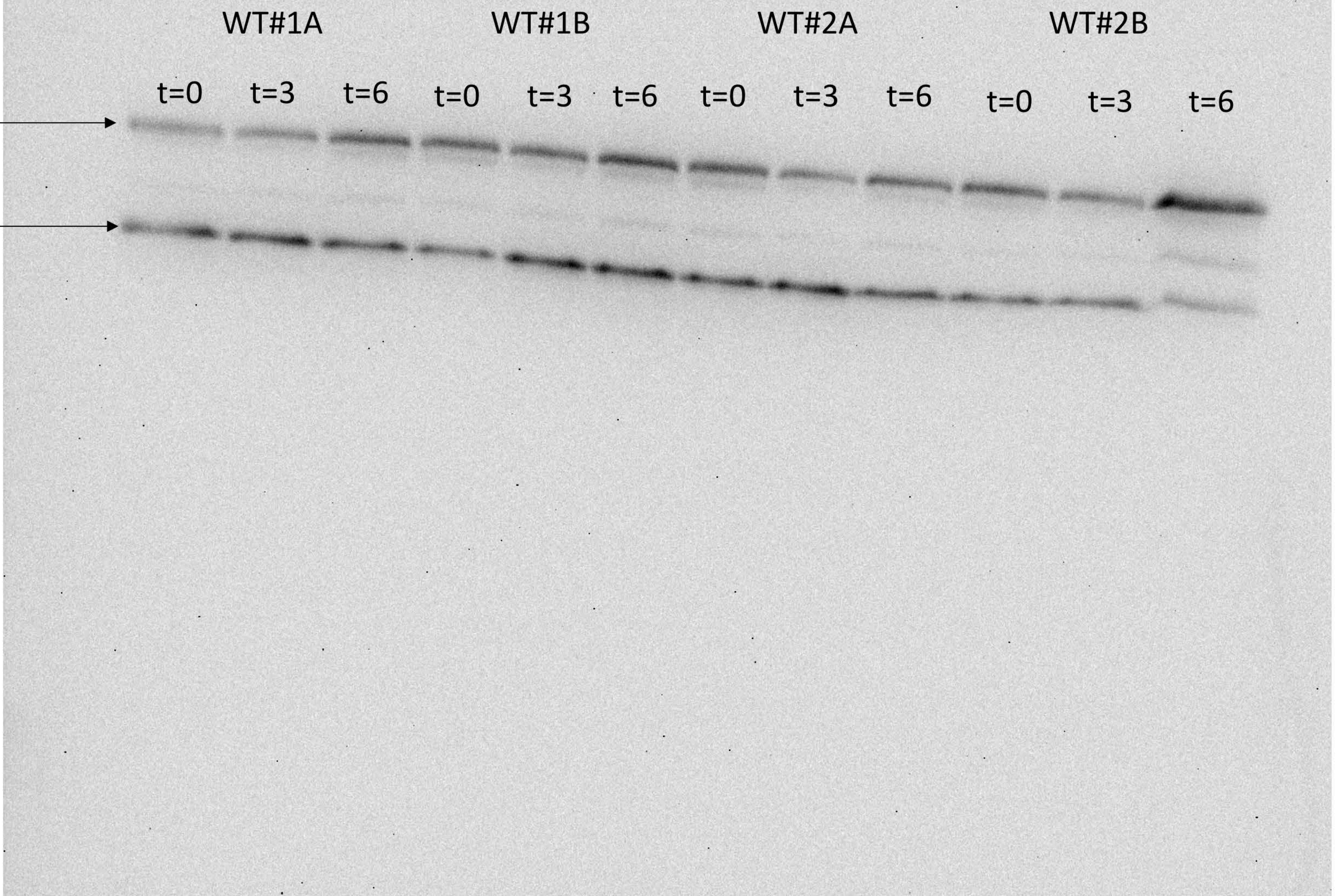

SKN7 WT compared to SKN7::GCNi
(set 2)

SKN7 GCNi (I7)
anti-TAP

SKN7+TAP (90kDa)

HMT1+TAP (60kDa)

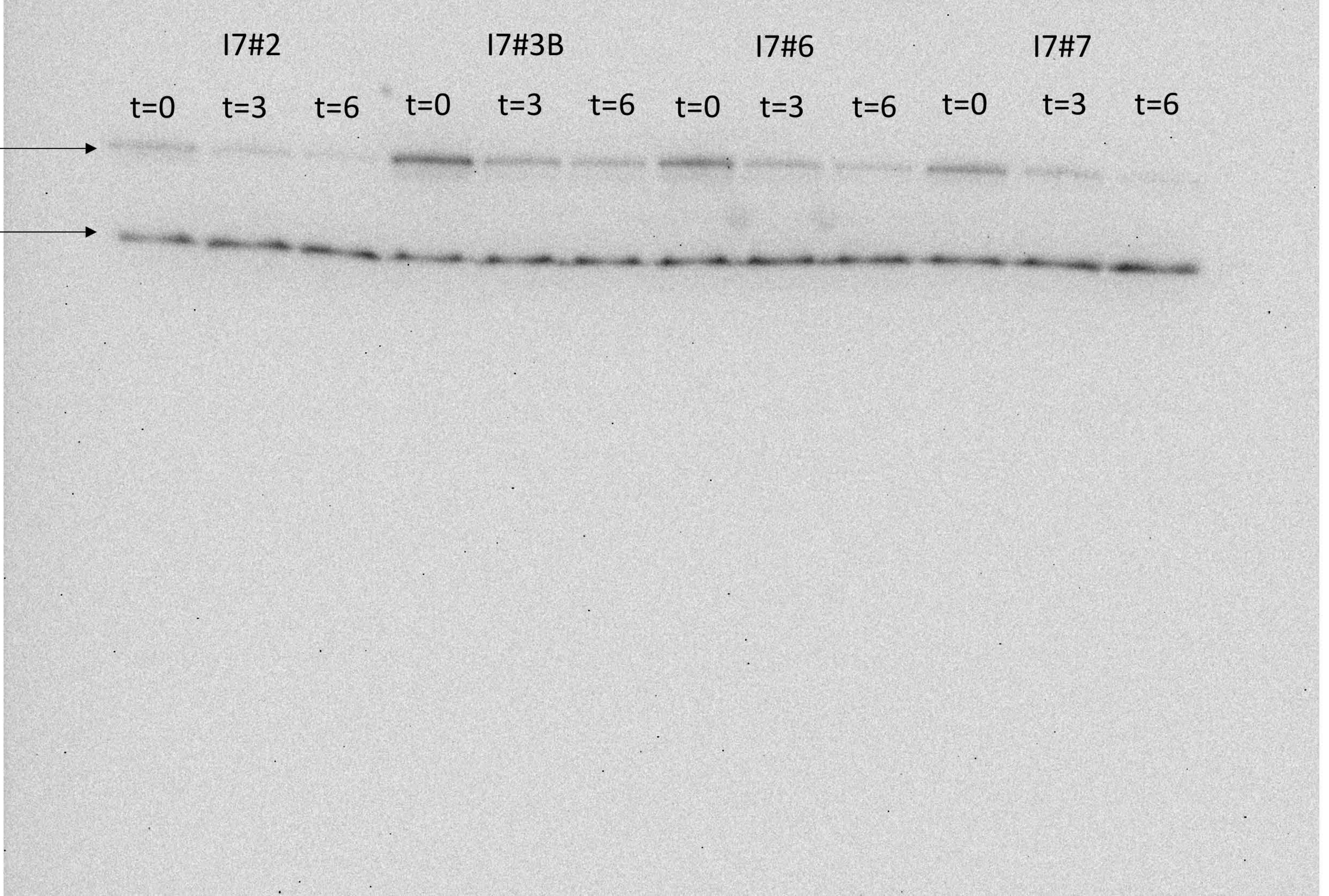

Raw Data for Fig 5D: Cycloheximide chase western images used for quantitation

HMT1 WT compared to HMT1::GCNpm

HMT1 WT
anti-TAP

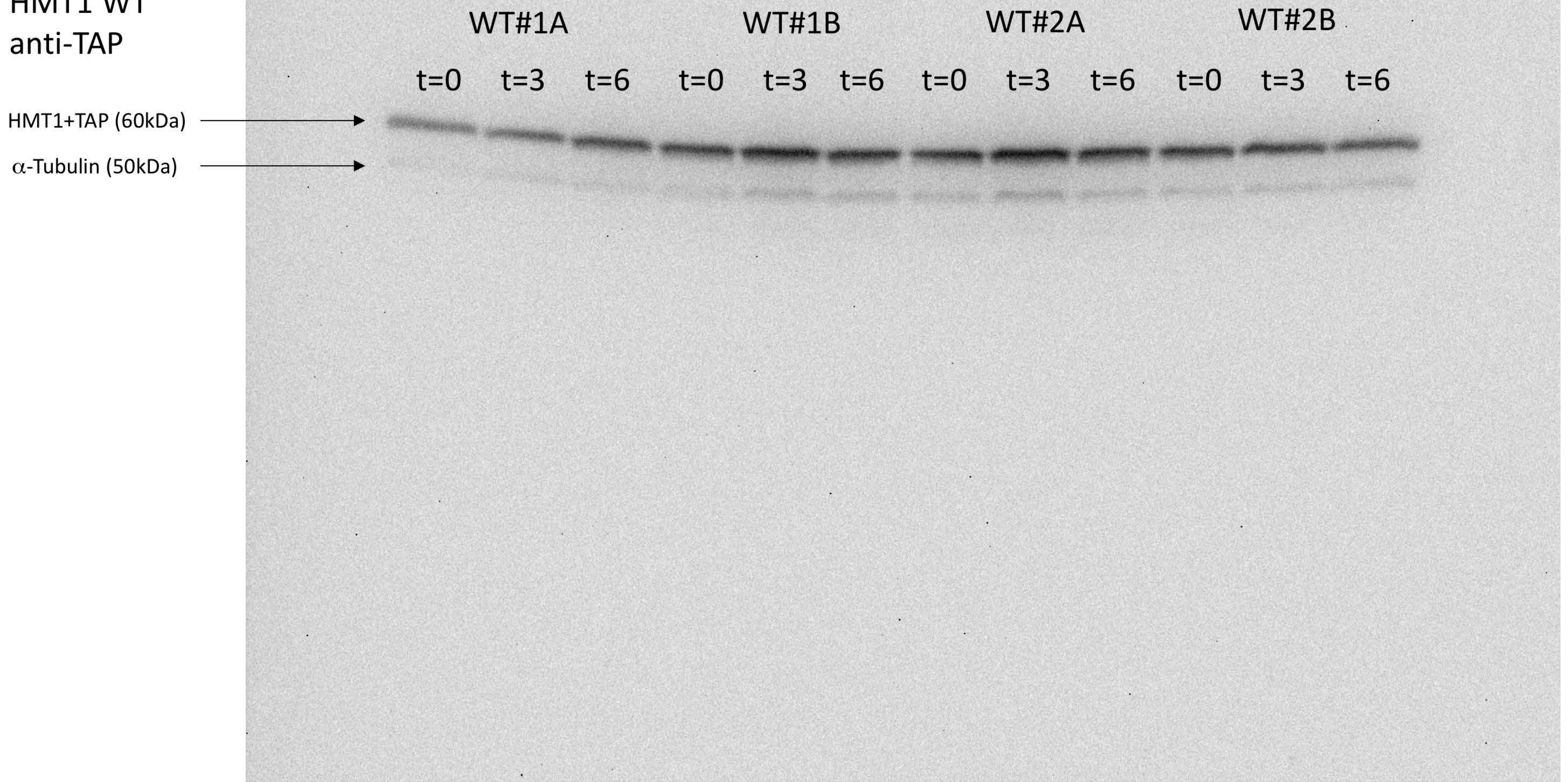

HMT1 WT compared to HMT1::GCNpm

HMT1 WT
anti-Tub

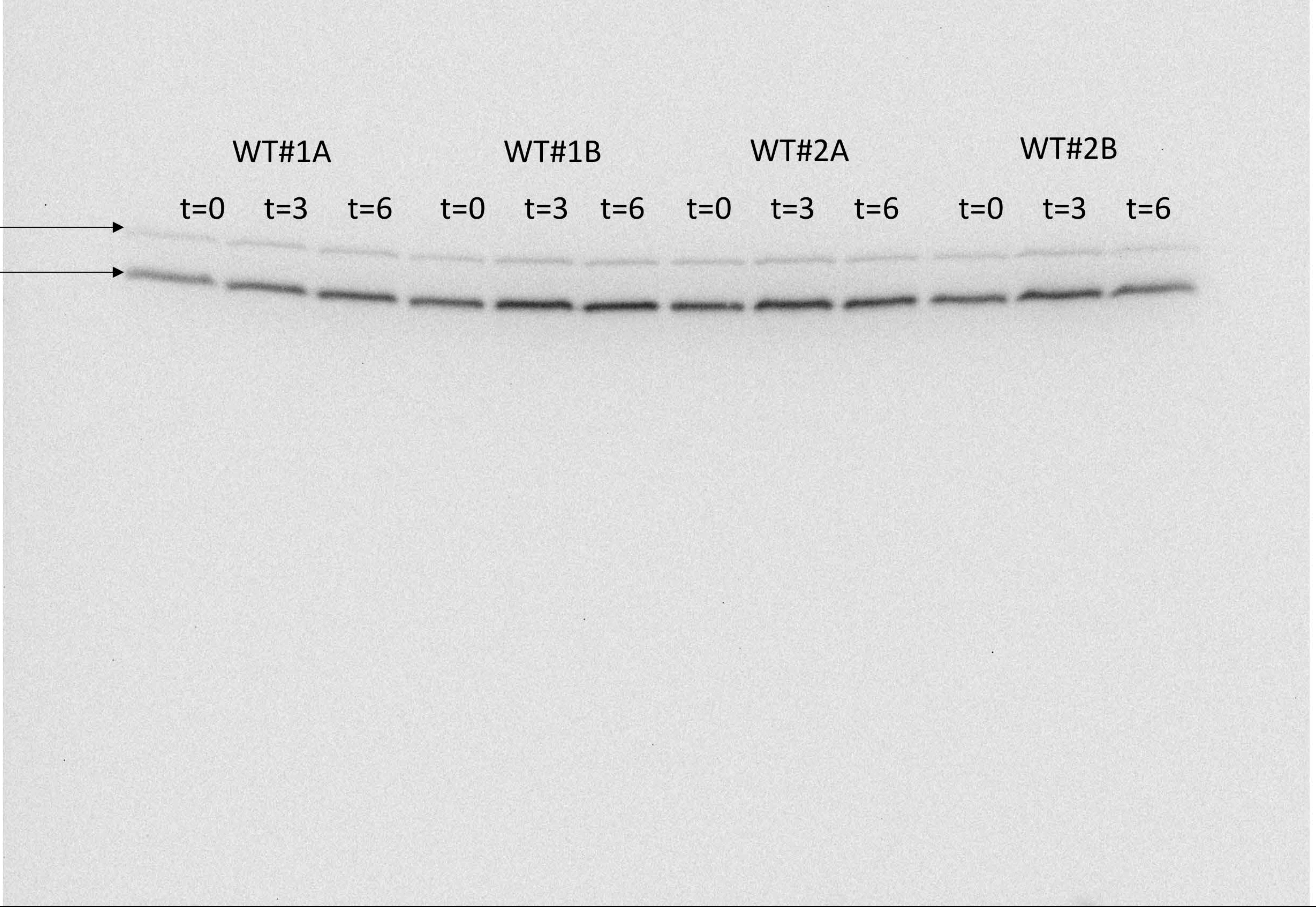

HMT1 WT compared to HMT1::GCNpm

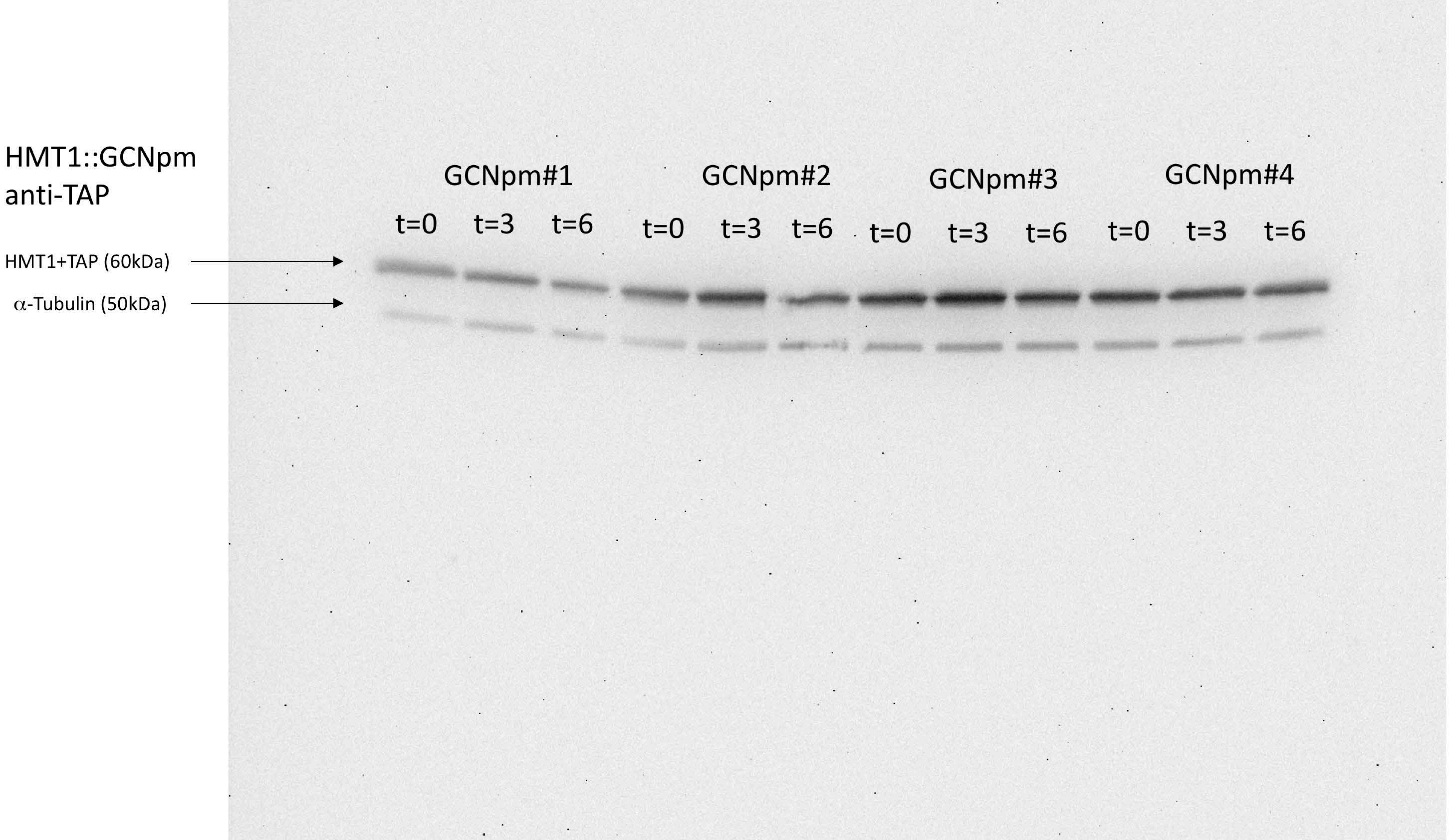

HMT1 WT compared to HMT1::GCNpm

HMT1::GCNpm
anti-Tub

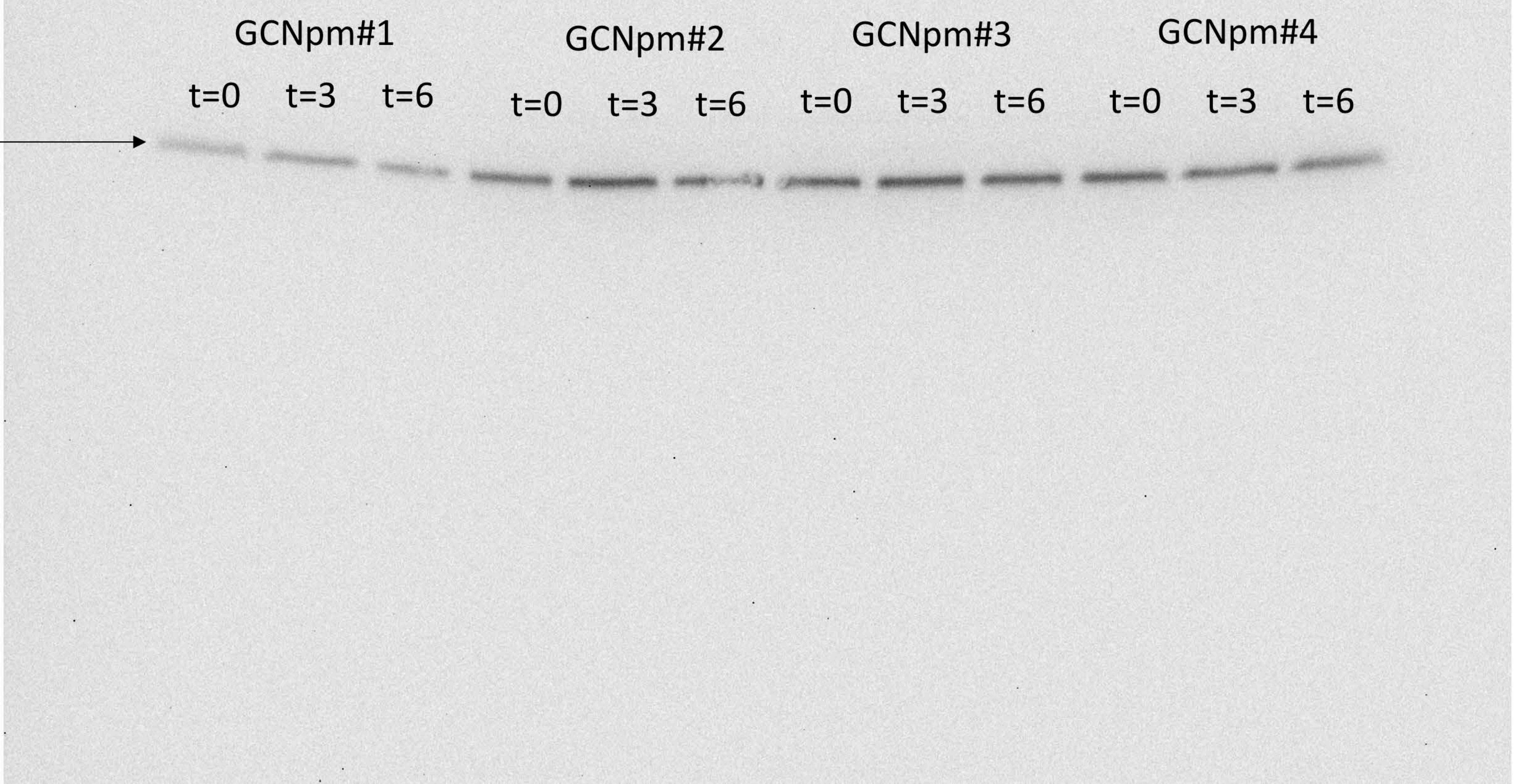

HMT1 WT compared to HMT1::G2

HMT1 WT
anti-TAP

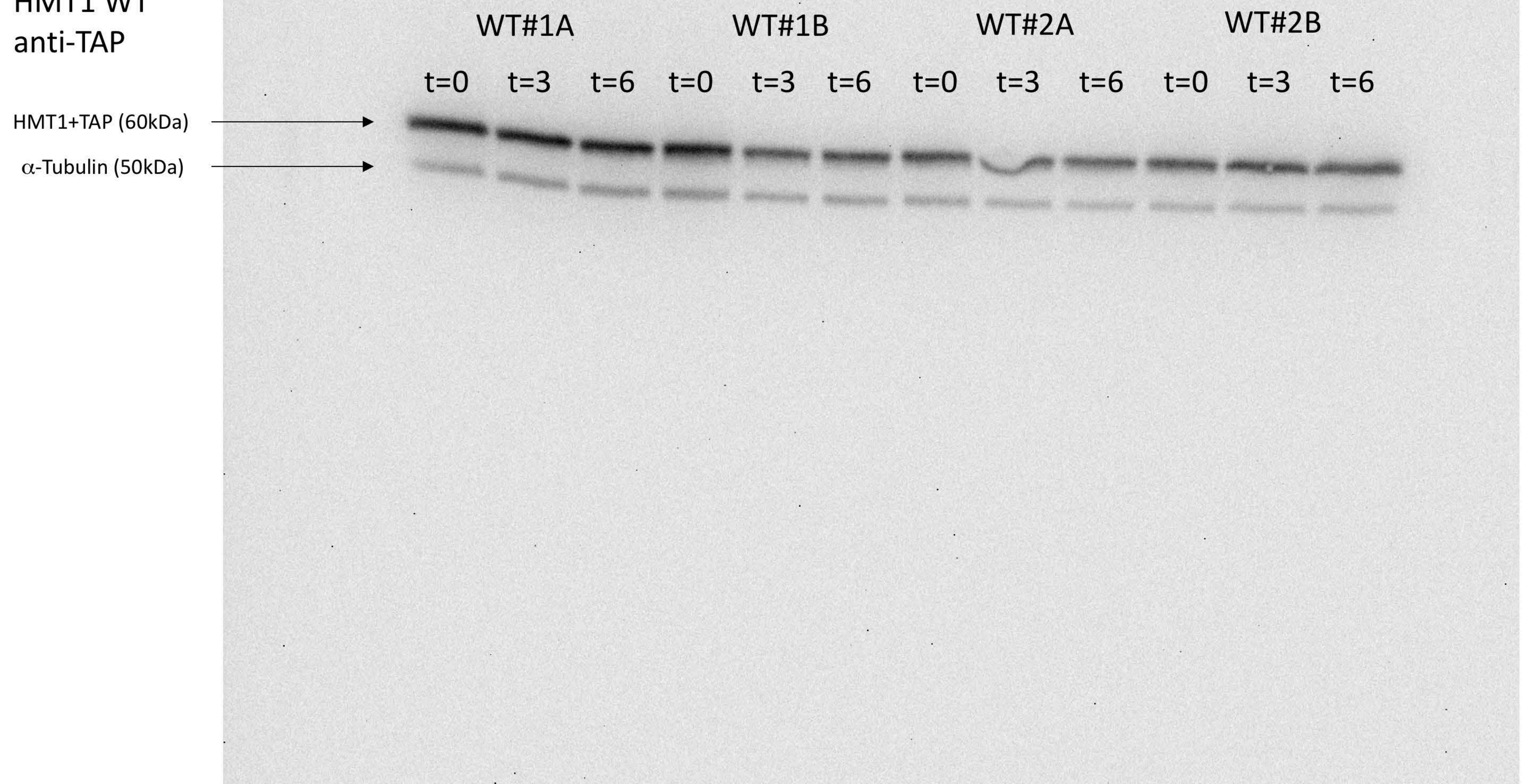

HMT1 WT compared to HMT1::G2

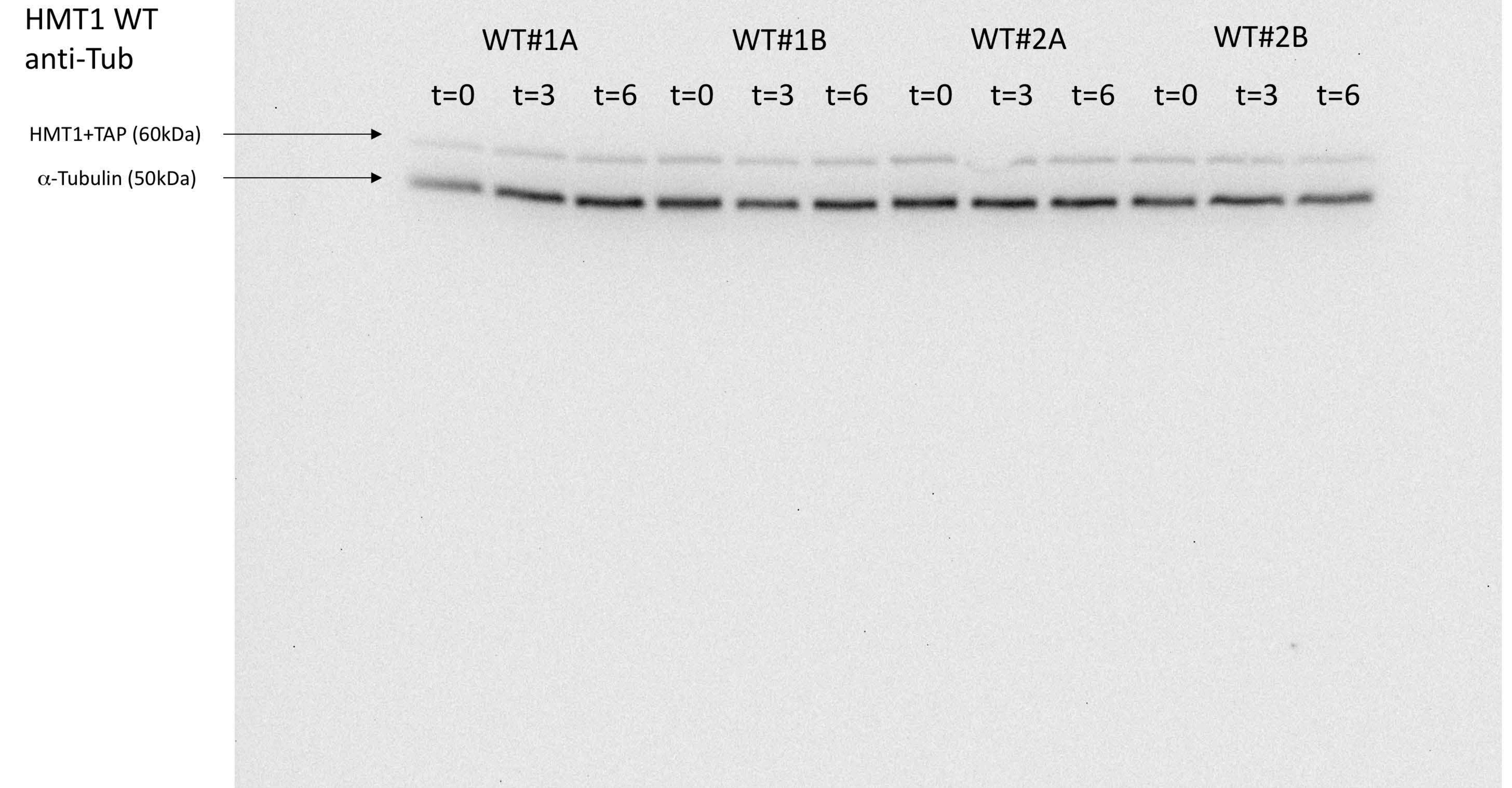

HMT1 WT compared to HMT1::G2

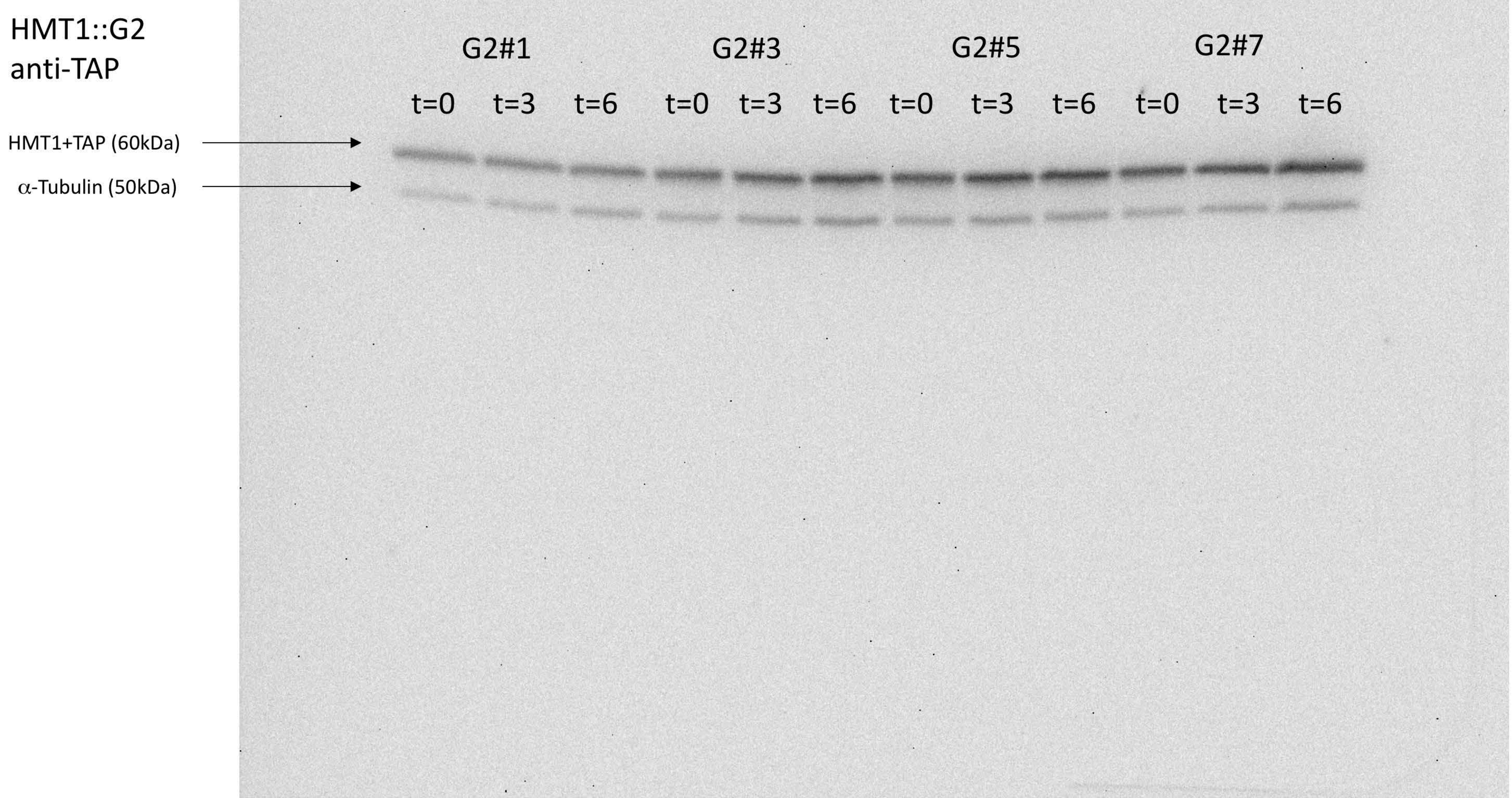

HMT1 WT compared to HMT1::G2

HMT1::G2
anti-Tub

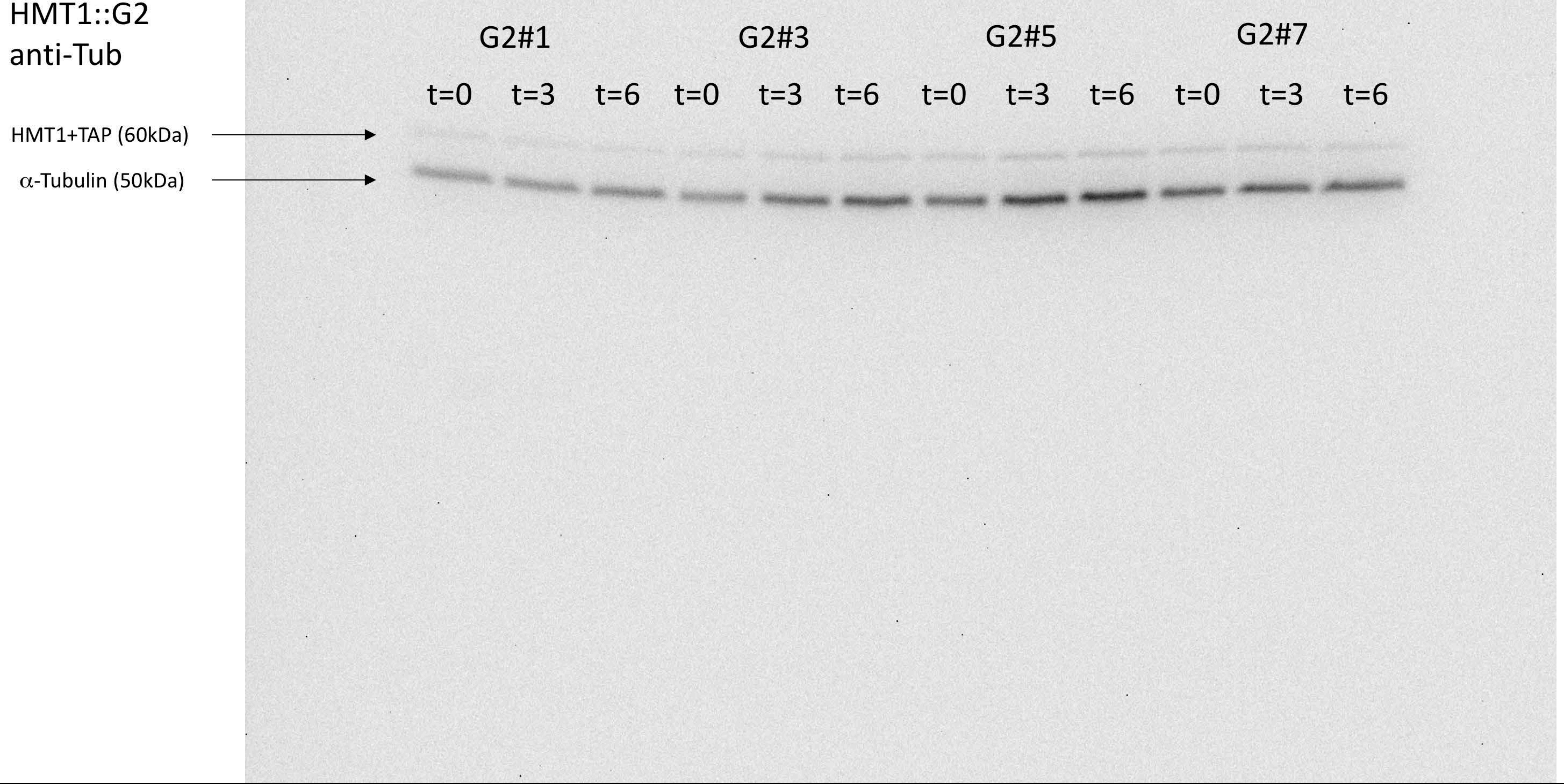

HMT1 WT compared to HMT1::C1

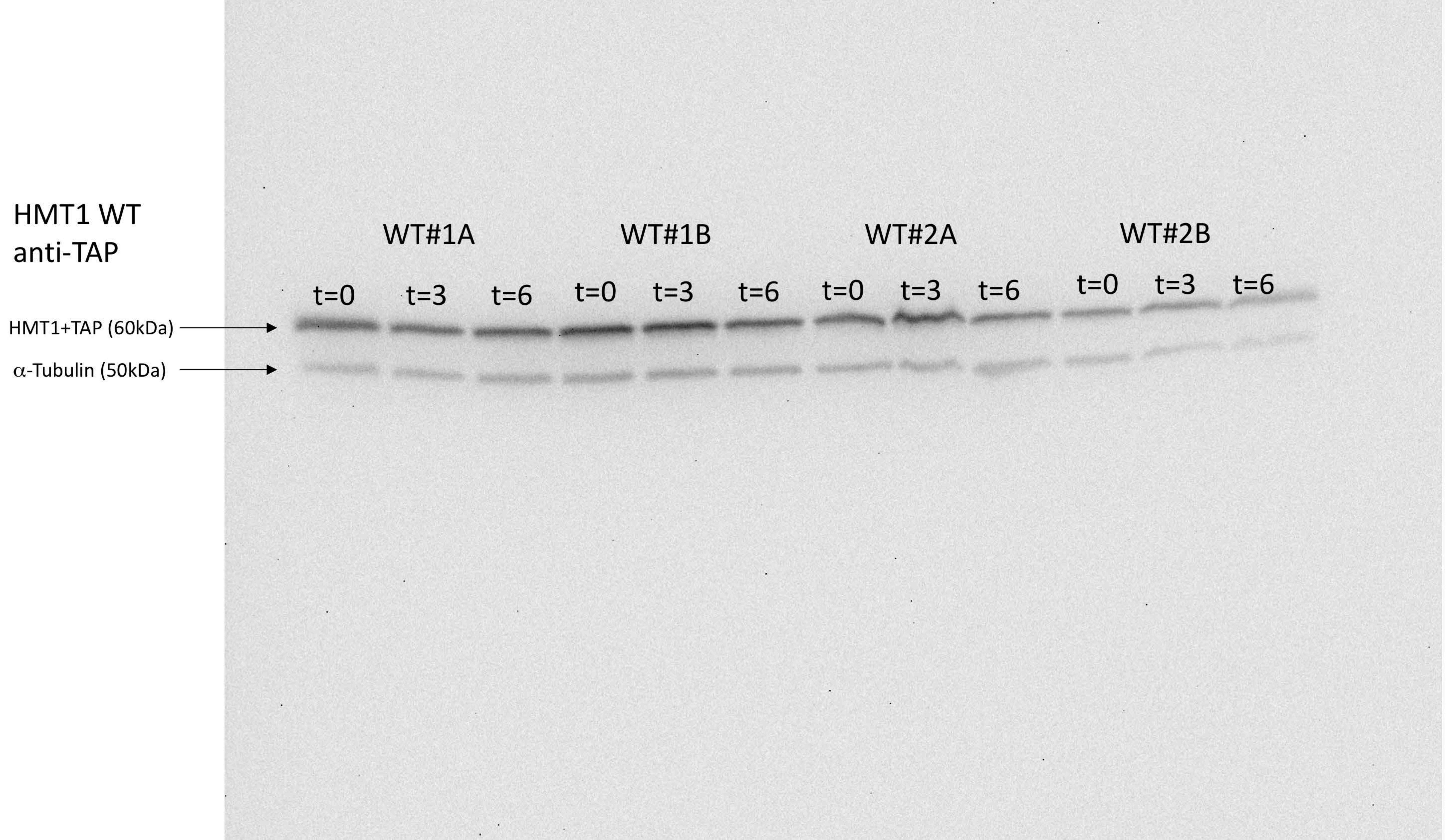

HMT1 WT compared to HMT1::C1

HMT1 WT
anti-Tub

HMT1+TAP (60kDa)
α-Tubulin (50kDa)

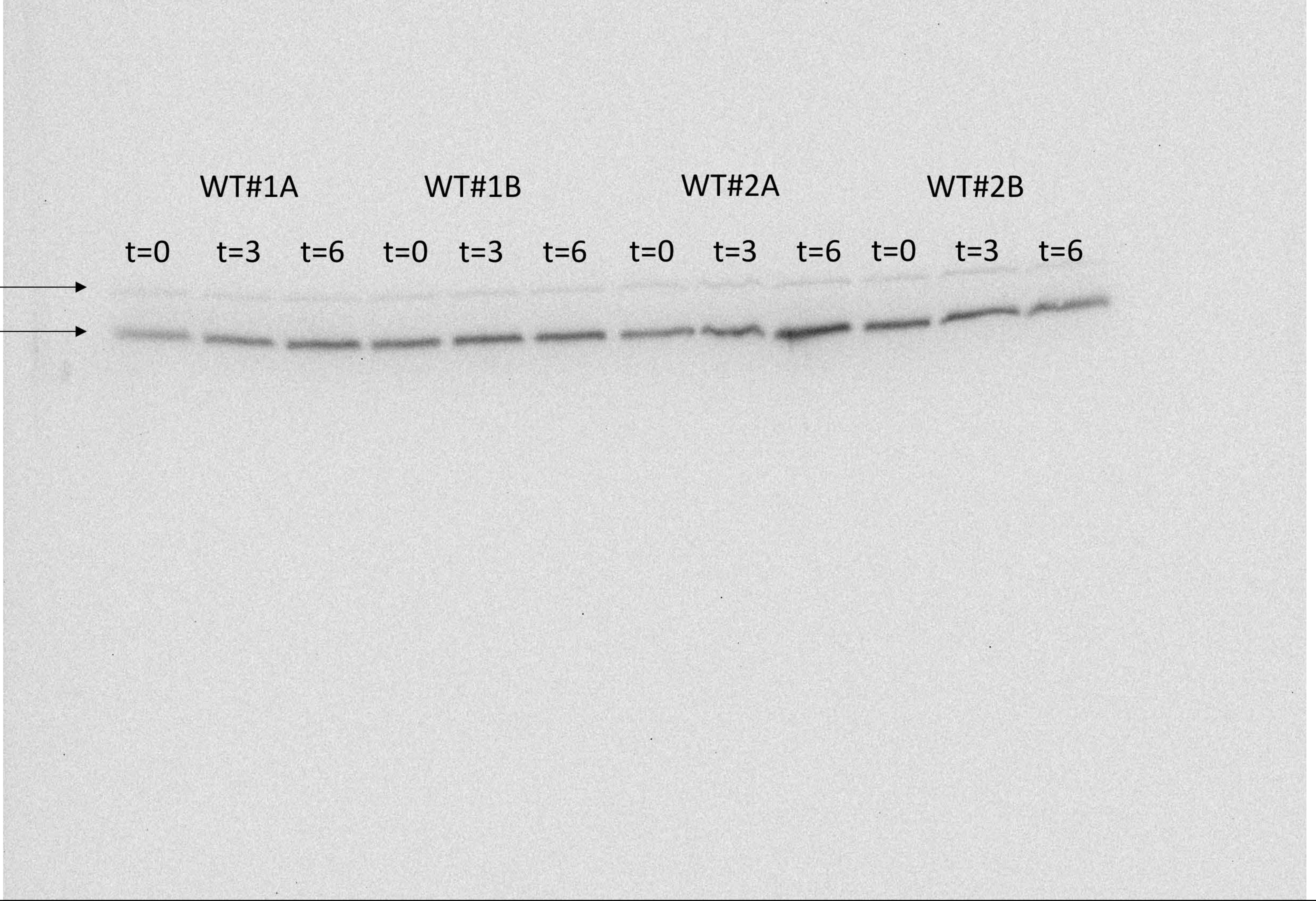

HMT1 WT compared to HMT1::C1

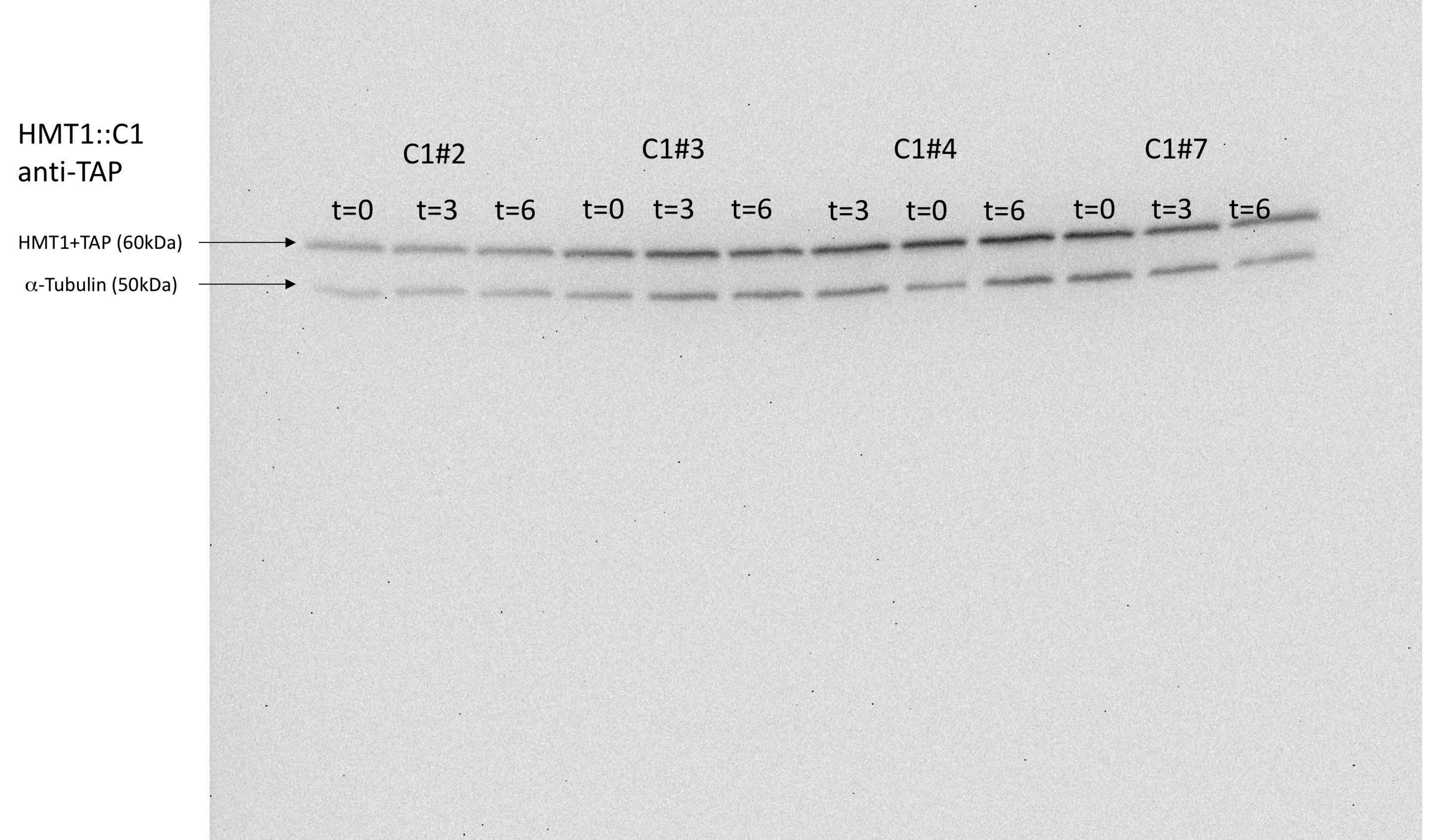

HMT1 WT compared to HMT1::C1

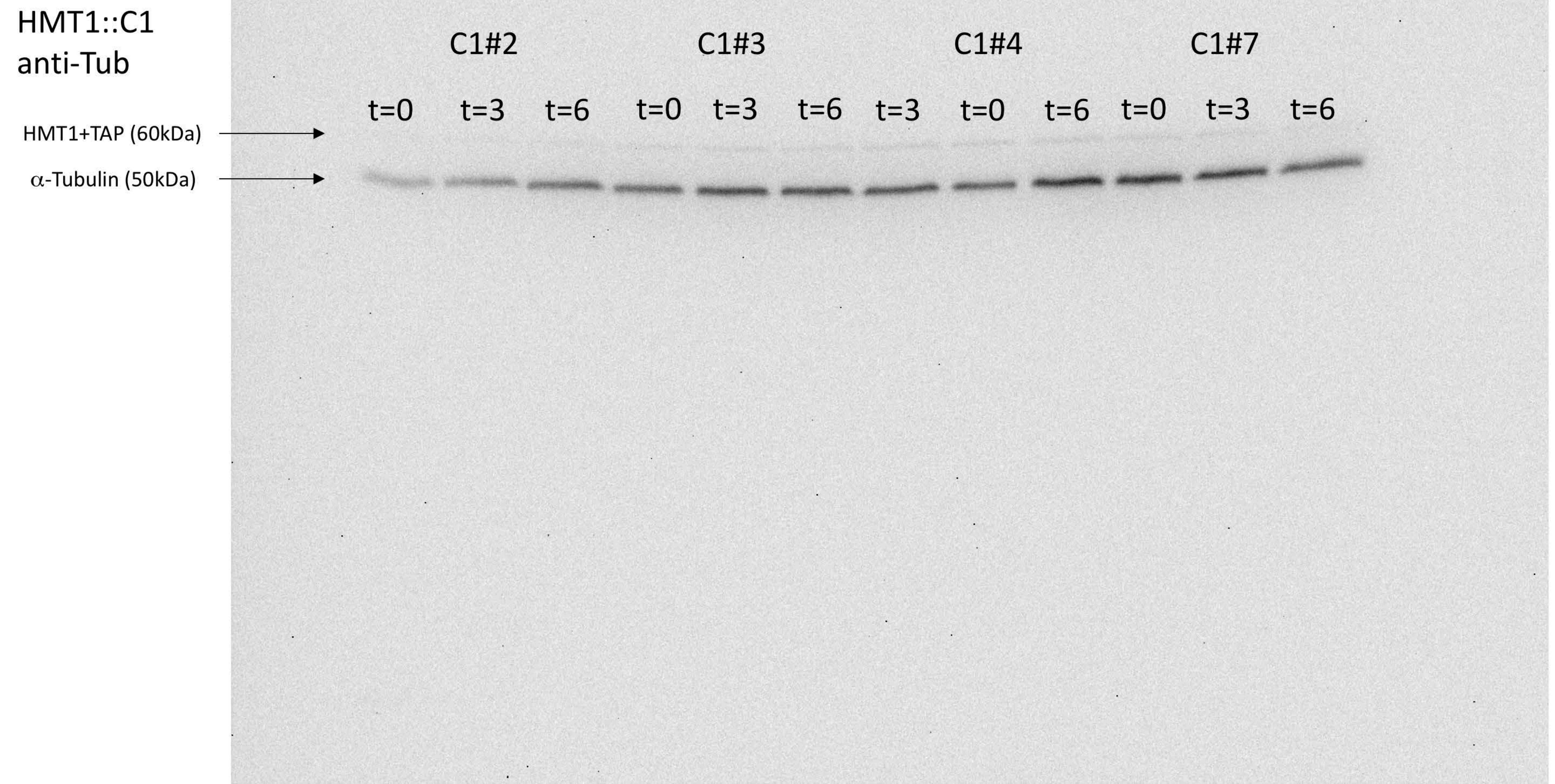

Supplement: S3 Fig — To measure protein stability, protein synthesis was blocked with cycloheximide (CYC) and samples collected at 0, 3 and 6h. Protein levels after 3h were used to compare protein stability (see Fig 5). As a loading control for SKN7-TAP quantitation, samples were spiked with HTM1-TAP (that was not exposed to CYC). As a loading control for HTM1 quantitation, blots were probed for Tub1 (α-tubulin; Tub antibody) protein which has a half life of 20h. Blots were probed with anti-TUB and then anti-TAP. The ramp mutant proteins were each expressed in multiple independent cultures (# labels). (PDF) [file pone.0233197.s003.pdf]
