# Supplementary material for: GCN sensitive protein translation in yeast
Source: PLoS One. 2020 Sep 18;15(9):e0233197. doi: 10.1371/journal.pone.0233197 (PMC7500604; doi:10.1371/journal.pone.0233197)
Supplement: S4 Fig — Predicted RNA secondary structures for SKN7 (A) and HMT1 (B) mutants and wildtype sequence. The lowest energy structures predicted by mFOLD for each strain is represented by a hairpin diagram. The energy of this structure is reported in the fourth column. In the last column, nucleotides -10 to +70 relative to the AUG start codon are presented above dot-bracket representations of all secondary structures reported by mFOLD using default parameters. The dot-bracket representation is followed by the corresponding dG free energy value for that structure. The predicted secondary structures for HMT1::G2 and HMT1::GCNpm have slightly lower dG values than the other predicted structures. (PDF) [file pone.0233197.s004.pdf]

S4 Fig

A

Gene	Mutant	Structure (Lowest dG)	Energy (dG)	Other structures
SKN7	*WT*	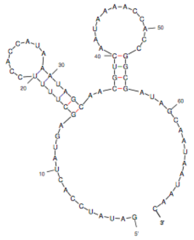	-4.20 kcal/mol	GAUAUCCACU**AUG**AGCUUUUCCACCAUAAAUAGCAACGUCAAUAAAACCACCGGCGAUAGCAAUAAUAAC<br>..............(((.((........)).)))..((((............)))).............. (-4.20)<br>........((((..(((.((........)).)))..((((............)))))))).......... (-2.20)<br>.........((((...........))))....((..((((............))))...))......... (-4.00)<br>..(((...((((..(((.((........)).)))..((((............))))))))..)))..... (-1.30)
SKN7	*G2*	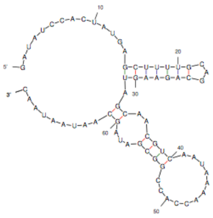	-8.30 kcal/mol	GAUAUCCACU**AUG**AGCUUUUGCAGCAGAAGUAGCAACGUCAAUAAAACCACCGGCGAUAGCAAUAAUAAC<br>..............(((((((...))))))).((..((((............))))...))......... (-8.70)<br>..............(((.((((.((....)).))))((((............))))..)))......... (-6.20)<br>..................((((.((.......))..((((............))))...))))....... (-4.00)<br>..............(((.((((.((.......))...((.......)).....)))).)))......... (-4.15)<br>.........(((..(((.((((.((....)).))))((((............))))..))).)))..... (-5.30)
SKN7	*GCNi*	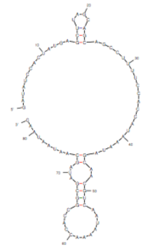	-6.10 kcal/mol	GAUAUCCACU**AUG**AGCUUAGCAGCAGCCUUUUCCACCAUAAAUAGCAACGUCAAUAAAACCACCGGCGAUAGCAAUAAUAAC<br>..............(((....)))....................((..((((............))))...))......... (-6.10)<br>...................((....((..(((.......)))..))..((((............))))...))......... (-1.65)<br>..............(((........((..(((.......)))..))..((((............))))..)))......... (-2.24)<br>........(((......)))..((.((..(((.......)))..))..((((............))))...))......... (-2.10)<br>(((...........(((....))).((..(((.......)))..))...))).............((....))......... (-1.49)
SKN7	*GCNpm*	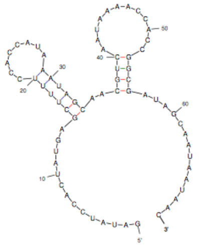	-6.90 kcal/mol	GAUAUCCACU**AUG**AGCGCCUCCACCAUAGCAGCCAACGUCAAUAAAACCACCGGCGAUAGCAAUAAUAAC<br>........(((((...........)))))..((...((((............))))...))......... (-6.90)
SKN7	*A-rich*	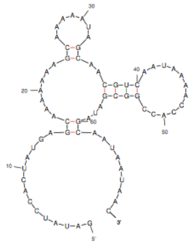	-6.30 kcal/mol	GAUAUCCACU**AUG**AGCAAAAAAAGCAAAAAUAGCAACGUCAAUAAAACCACCGGCGAUAGCAAUAAUAAC<br>..............((.......((.......))..((((............))))...))......... (-1.84)<br>.........(((..((.......((.......))..((((............))))...)).)))..... (-0.94)

B

Gene	Mutant	Structure (Lowest dG)	Energy (dG)	Other structures
HMT1	*WT*	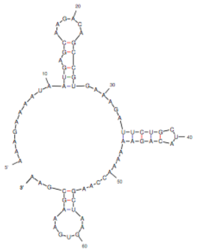	-6.70 kcal/mol	AAAGAAAAUA**AUG**AGCAAGACAGCCGUGAAAGAUUCUGCUACAGAAAAAACCAAGCUAAGUGAAAGCGAA<br>..........(((.((......)))))......(((((...))))).........(((.......)))... (-6.70)<br>.............(((...((....))......(((((...)))))........)))..((....))... (-1.75)<br>.....................(((..((.....(((((...))))).....)).)))..((....))... (-5.90)<br>..((((....(((.((......)))))......)))).................(((.......)))... (-5.90)
HMT1	*G2*	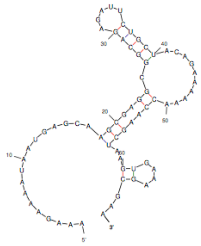	-10.50 kcal/mol	AAAGAAAAUA**AUG**AGCAAGCGAGGCGGCAGAGAUUCUGCUACAGAAAAAACCAAGCUAAGUGAAAGCGAA<br>.................(((..((.(((((.....)))))..........))..)))..((....))... (-10.50)<br>..............((..((..((((((((.....)))))..............)))..))....))... (-10.10)<br>.............(((..((...)).((((.....)))).............. .)))..((....))... (-5.89)
HMT1	*C1*	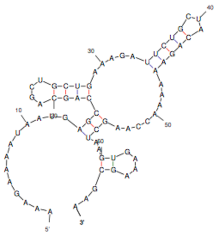	-8.65 kcal/mol	AAAGAAAAUA**AUG**AGCCAGCAGCUGCUGAAAGAUUCUGCUACAGAAAAAACCAAGCUAAGUGAAAGCGAA<br>.............(((((((....)))).....(((((...)))))........)))..((....))... (-8.65)<br>................((((....)))).....(((((...))))).........(((.......)))... (-9.60)
HMT1	*GCNpm*	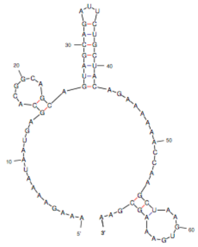	-12.80 kcal/mol	AAAGAAAAUA**AUG**AGCACGGCAGCAGUAGCAGAUUCUGCUACAGAAAAAACCAAGCUAAGUGAAAGCGAA<br>..............((......)).(((((((...)))))))............(((.......)))... (-12.80)<br>.............(((..((.....(((((((...)))))))........))..)))..((....))... (-12.60)

S4 Fig. RNA secondary structure prediction. Predicted RNA secondary structures for *SKN7* (A) and *HMT1* (B) mutants and wildtype sequence. The lowest energy structures predicted by mFOLD for each strain is represented by a hairpin diagram. The energy of this structure is reported in the fourth column. In the last column, nucleotides -10 to +70 relative to the AUG start codon are presented above dot-bracket representations of all secondary structures reported by mFOLD using default parameters. The dot-bracket representation is followed by the corresponding dG free energy value for that structure. The predicted secondary structures for *HMT1::G2* and *HMT1::GCNpm* have slightly lower dG values than the other predicted structures.
