# Supplementary material for: GCN sensitive protein translation in yeast
Source: PLoS One. 2020 Sep 18;15(9):e0233197. doi: 10.1371/journal.pone.0233197 (PMC7500604; doi:10.1371/journal.pone.0233197)
Supplement: S5 Fig — Yeast expression of 35,811 reporter constructs with different nine-mer sequences at codons 6–8 [13]. Reporter expression is divided into three bins, low, intermediate and high. Expression is graphed as described in S6 Fig. The shapes of the graphs suggest that at lower levels of expression, higher densities of G1 and C2 are beneficial. The effect is more pronounced for codons 6 and 7 (B) or codons 7 and 8 (C) compared to codons 6 and 8 (D) suggesting that codon adjacency may increase the effect. The data do not show whether with higher expression levels, G1 and C2 are detrimental to expression, because the high expression bin has over 80% of the reporter sequences and therefore has limited resolution. (PDF) [file pone.0233197.s005.pdf]

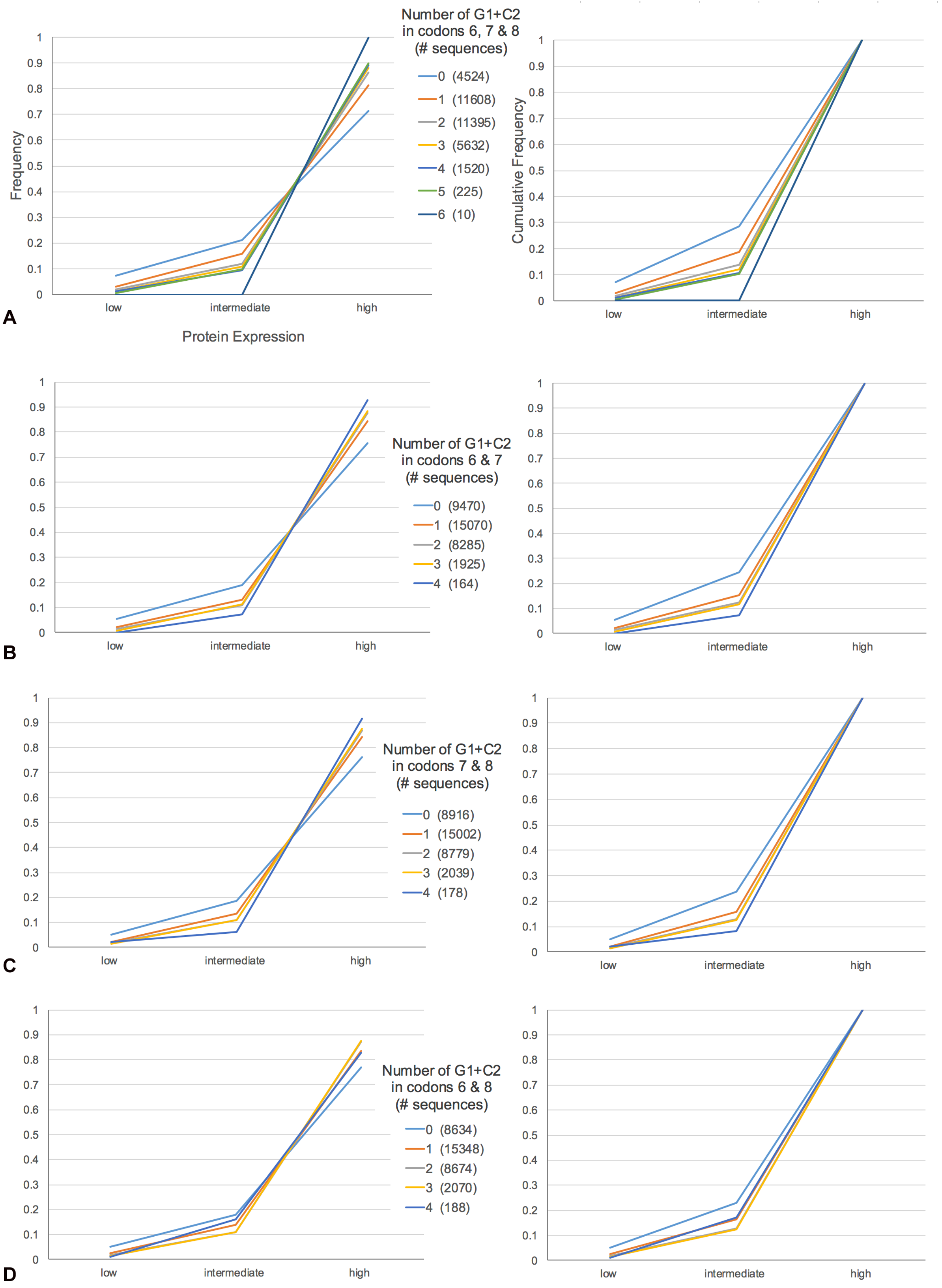

Number of G1+C2 in codons 6, 7 & 8 (# sequences)
0 (4524)
1 (11608)
2 (11395)
3 (5632)
4 (1520)
5 (225)
6 (10)
Frequency
Cumulative Frequency
low
intermediate
high
Protein Expression
A
Number of G1+C2 in codons 6 & 7 (# sequences)
0 (9470)
1 (15070)
2 (8285)
3 (1925)
4 (164)
B
Number of G1+C2 in codons 7 & 8 (# sequences)
0 (8916)
1 (15002)
2 (8779)
3 (2039)
4 (178)
C
Number of G1+C2 in codons 6 & 8 (# sequences)
0 (8634)
1 (15348)
2 (8674)
3 (2070)
4 (188)
D

S5 Fig. Large-scale reporter analysis in yeast. Yeast expression of 35,811 reporter constructs with different nine-mer sequences at codons 6-8 (Gamble *et al*. 2016). Reporter expression is divided into three bins, low, intermediate and high. Expression is graphed as described in S6 Fig. The shapes of the graphs suggest that at lower levels of expression, higher densities of G1 and C2 are beneficial. The effect is more pronounced for codons 6 and 7 (B) or codons 7 and 8 (C) compared to codons 6 and 8 (D) suggesting that codon adjacency may increase the effect. The data do not show whether with higher expression levels, G1 and C2 are detrimental to expression, because the high expression bin has over 80% of the reporter sequences and therefore has limited resolution.
