# Supplementary material for: GCN sensitive protein translation in yeast
Source: PLoS One. 2020 Sep 18;15(9):e0233197. doi: 10.1371/journal.pone.0233197 (PMC7500604; doi:10.1371/journal.pone.0233197)
Supplement: S6 Fig — Bacterial expression of 215,414 reporter constructs with different nine-mer sequences at codons 3–5 [31] allowed assignment of ramp sequences into nine expression ranges (1: 1< = x<1.5; 1.5: 1.5< = x<2;… 4.5: 4.5< = x<5; 5: x = 5). The nine-mers were divided into 5 groups based on the number of G1 and C2 nucleotides in two codons: codons 3 and 4 (A), codons 4 and 5 (B) or codons 3 and 5 (C). The expression histogram (left) and cumulative frequencies (right) for each group were graphed. The shapes of the graphs suggest that at lower levels of expression, higher densities of G1 and C2 are beneficial, but at higher expression levels, G1 and C2 are detrimental to expression. These effects are slightly more pronounced for codons 3 and 4 (A), compared to codons 4 and 5 (B), suggesting that codon 3 may have a little more influence than codon 4. The effects are also slightly more pronounced for codons 3 and 4 (A) compared to codons 3 and 5 (C) suggesting that codon adjacency may increase the effect. (PDF) [file pone.0233197.s006.pdf]

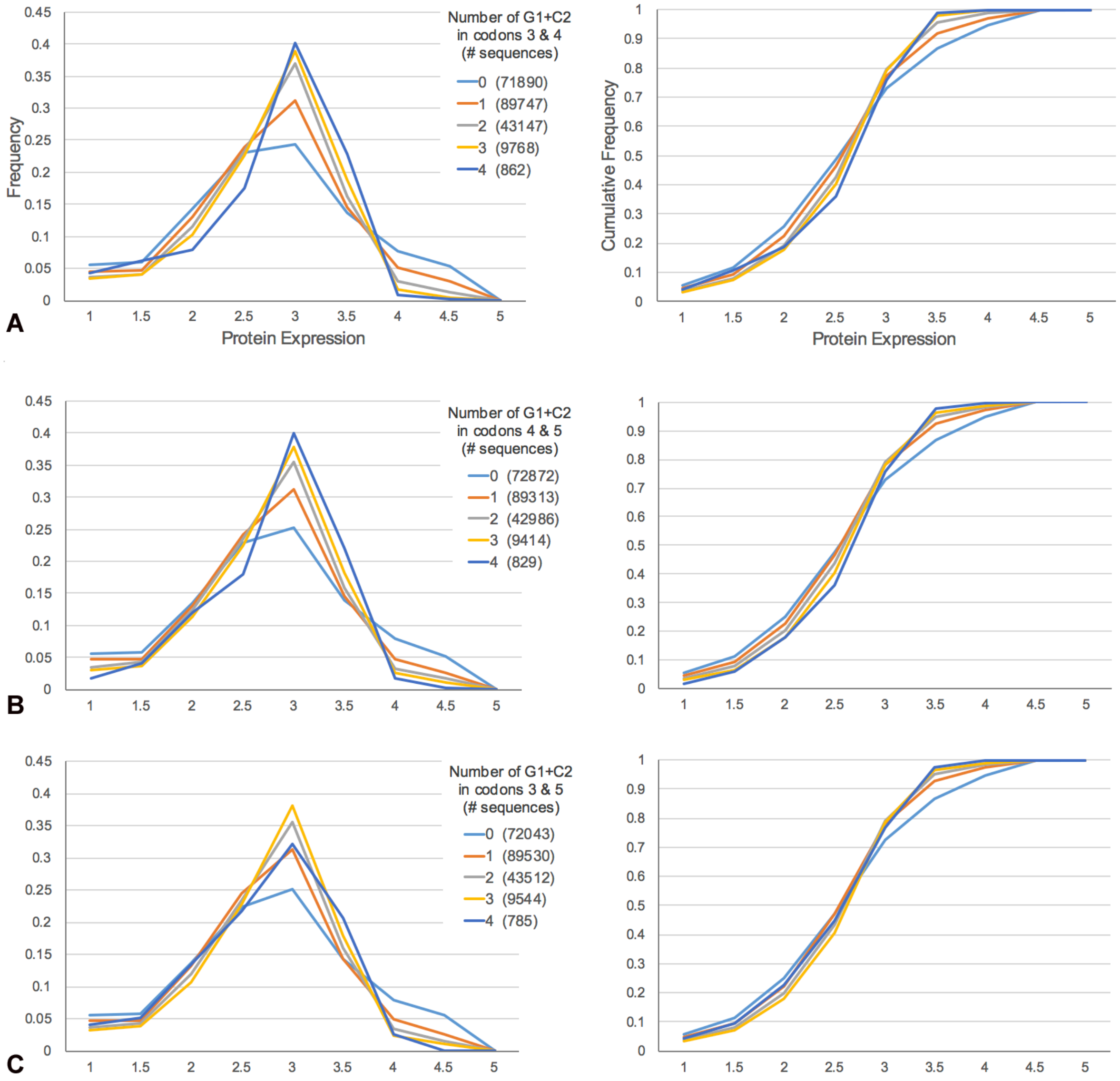

S6 Fig. Large-scale reporter analysis in bacteria. Bacterial expression of 215,414 reporter constructs with different nine-mer sequences at codons 3-5 (Verma *et al*. 2019) allowed assignment of ramp sequences into nine expression ranges (1: 1<=x<1.5; 1.5: 1.5<=x<2; ... 4.5: 4.5<=x<5; 5: x=5). The nine-mers were divided into 5 groups based on the number of G1 and C2 nucleotides in two codons: codons 3 and 4 (A), codons 4 and 5 (B) or codons 3 and 5 (C). The expression histogram (left) and cumulative frequencies (right) for each group were graphed. The shapes of the graphs suggest that at lower levels of expression, higher densities of G1 and C2 are beneficial, but at higher expression levels, G1 and C2 are detrimental to expression. These effects are slightly more pronounced for codons 3 and 4 (A), compared to codons 4 and 5 (B), suggesting that codon 3 may have a little more influence than codon 4. The effects are also slightly more pronounced for codons 3 and 4 (A) compared to codons 3 and 5 (C) suggesting that codon adjacency may increase the effect.
