# Supplementary material for: GCN sensitive protein translation in yeast
Source: PLoS One. 2020 Sep 18;15(9):e0233197. doi: 10.1371/journal.pone.0233197 (PMC7500604; doi:10.1371/journal.pone.0233197)
Supplement: S1 Table — Yeast strains for ramp mutants. Strains used in this study were generated as described in Methods. The table includes the name, strain ID, colony number, and genotype for each yeast strain used in this study. The first thirty nucleotides of the candidate gene sequence is included for each strain, with deviations from wildtype sequence highlighted in red lettering. (PDF) [file pone.0233197.s007.pdf]

S1 Table. Yeast strains for ramp mutants. Yeast strains for ramp mutants. Strains used in this study were generated as described in Methods. The table includes the name, strain ID, colony number, and genotype for each yeast strain used in this study. The first thirty nucleotides of the candidate gene sequence is included for each strain, with deviations from wildtype sequence highlighted in red lettering.

Strain ID	Colony Number	Mutant	Genotype	Sequence
YMW001	1	*SKN7-TAP-URA3*	**MATa** *his3*Δ1 *leu2*Δ0 *met15*Δ0 *SKN7-TAP-URA3*	ATGAGCTTTTCCACCA TAAATAGCAACGTC
YMW002	2	*SKN7-TAP-URA3*	**MATa** *his3*Δ1 *leu2*Δ0 *met15*Δ0 *SKN7-TAP-URA3*	ATGAGCTTTTCCACCA TAAATAGCAACGTC
YMW003	1	*HMT1-TAP-URA3*	**MATa** *his3*Δ1 *leu2*Δ0 *met15*Δ0 *SKN7-TAP-URA3*	ATGAGCAAGACAGCC GTGAAAGATTCTGCT
YMW004	2	*HMT1-TAP-URA3*	**MATa** *his3*Δ1 *leu2*Δ0 *met15*Δ0 *HMT1-TAP-URA3*	ATGAGCAAGACAGCC GTGAAAGATTCTGCT
YMW005	1	*skn7::kanMX6*	**MATa** *his3*Δ1 *leu2*Δ0 *met15*Δ0 *skn7::kanMX6-TAP-URA3*	ATGAGCTTTTCCACCA TAAATAGCAACGTC
YMW006	2	*skn7::kanMX6*	**MATa** *his3*Δ1 *leu2*Δ0 *met15*Δ0 *skn7::kanMX6-TAP-URA3*	ATGAGCTTTTCCACCA TAAATAGCAACGTC
YMW007	1	*skn7::hphMX4*	**MATa** *his3*Δ1 *leu2*Δ0 *met15*Δ0 *skn7::hphMX4-TAP-URA3*	ATGAGCTTTTCCACCA TAAATAGCAACGTC
YMW008	2	*skn7::hphMX4*	**MATa** *his3*Δ1 *leu2*Δ0 *met15*Δ0 *skn7::hphMX4-TAP-URA3*	ATGAGCTTTTCCACCA TAAATAGCAACGTC
YMW009	1	*hmt1::kanMX6*	**MATa** *his3*Δ1 *leu2*Δ0 *met15*Δ0 *hmt1::kanMX6-TAP-URA3*	ATGAGCAAGACAGCC GTGAAAGATTCTGCT
YMW010	2	*hmt1::kanMX6*	**MATa** *his3*Δ1 *leu2*Δ0 *met15*Δ0 *hmt1::kanMX6-TAP-URA3*	ATGAGCAAGACAGCC GTGAAAGATTCTGCT

YMW011	1	*hmt1::hphMX4*	**MATa** *his3Δ1 leu2Δ0 met15Δ0 hmt1::hphMX4-TAP-URA3*	ATGAGCAAGACAGCCGTGAAAGATTCTGCT
YMW012	2	*hmt1::hphMX4*	**MATa** *his3Δ1 leu2Δ0 met15Δ0 hmt1::hphMX4-TAP-URA3*	ATGAGCAAGACAGCCGTGAAAGATTCTGCT
YMW013	H1	*SKN7::G2*	**MATa** *his3Δ1 leu2Δ0 met15Δ0 skn7::G2-TAP-URA3*	ATGAGCTTTTGCAGCAGAAGTAGCAACGTC
YMW014	H3	*SKN7::G2*	**MATa** *his3Δ1 leu2Δ0 met15Δ0 skn7::G2-TAP-URA3*	ATGAGCTTTTGCAGCAGAAGTAGCAACGTC
YMW015	A1	*SKN7::G2*	**MATa** *his3Δ1 leu2Δ0 met15Δ0 skn7::G2-TAP-URA3*	ATGAGCTTTTGCAGCAGAAGTAGCAACGTC
YMW016	A2	*SKN7::G2*	**MATa** *his3Δ1 leu2Δ0 met15Δ0 skn7::G2-TAP-URA3*	ATGAGCTTTTGCAGCAGAAGTAGCAACGTC
YMW017	2	*SKN7::GCNi*	**MATa** *his3Δ1 leu2Δ0 met15Δ0 skn7::GCNi-TAP-URA3*	ATGAGCTTAGCAGCAGCCTTTTCCACCATA
YMW018	3A	*SKN7::GCNi*	**MATa** *his3Δ1 leu2Δ0 met15Δ0 skn7::GCNi-TAP-URA3*	ATGAGCTTAGCAGCAGCCTTTTCCACCATA
YMW019	6	*SKN7::GCNi*	**MATa** *his3Δ1 leu2Δ0 met15Δ0 skn7::GCNi-TAP-URA3*	ATGAGCTTAGCAGCAGCCTTTTCCACCATA
YMW020	7	*SKN7::GCNi*	**MATa** *his3Δ1 leu2Δ0 met15Δ0 skn7::GCNi-TAP-URA3*	ATGAGCTTAGCAGCAGCCTTTTCCACCATA
YMW021	5A	*SKN7::GCNpm*	**MATa** *his3Δ1 leu2Δ0 met15Δ0 skn7::GCNpm-TAP-URA3*	ATGAGCGCCTCCACCATAGCAGCCAACGTC
YMW022	8	*SKN7::GCNpm*	**MATa** *his3Δ1 leu2Δ0 met15Δ0 skn7::GCNpm-TAP-URA3*	ATGAGCGCCTCCACCATAGCAGCCAACGTC
YMW023	A	*SKN7::GCNpm*	**MATa** *his3Δ1 leu2Δ0 met15Δ0*	ATGAGCGCCTCCACCATAGCAGCCAACGTC

			skn7::GCNpm-TAP-URA3	
YMW024	F	*SKN7::GCNpm*	**MATa** *his3Δ1 leu2Δ0 met15Δ0 skn7::GCNpm-TAP-URA3*	ATGAGCGCCTCCACCATAGCAGCCAACGTC
YMW025	5A	*SKN7::A-rich*	**MATa** *his3Δ1 leu2Δ0 met15Δ0 skn7::A-rich-TAP-URA3*	ATGAGCAAAAAAAGCAAAAATAGCAACGTC
YMW026	5B	*SKN7::A-rich*	**MATa** *his3Δ1 leu2Δ0 met15Δ0 skn7::A-rich-TAP-URA3*	ATGAGCAAAAAAAGCAAAAATAGCAACGTC
YMW027	6A	*SKN7::A-rich*	**MATa** *his3Δ1 leu2Δ0 met15Δ0 skn7::A-rich-TAP-URA3*	ATGAGCAAAAAAAGCAAAAATAGCAACGTC
YMW028	8	*SKN7::A-rich*	**MATa** *his3Δ1 leu2Δ0 met15Δ0 skn7::A-rich-TAP-URA3*	ATGAGCAAAAAAAGCAAAAATAGCAACGTC
YMW029	1	*HMT1::G2*	**MATa** *his3Δ1 leu2Δ0 met15Δ0 hmt1::G2-TAP-URA3*	ATGAGCAAGCGAGGCGGCAGAGATTCTGCT
YMW030	2	*HMT1::G2*	**MATa** *his3Δ1 leu2Δ0 met15Δ0 hmt1::G2-TAP-URA3*	ATGAGCAAGCGAGGCGGCAGAGATTCTGCT
YMW031	3	*HMT1::G2*	**MATa** *his3Δ1 leu2Δ0 met15Δ0 hmt1::G2-TAP-URA3*	ATGAGCAAGCGAGGCGGCAGAGATTCTGCT
YMW032	5	*HMT1::G2*	**MATa** *his3Δ1 leu2Δ0 met15Δ0 hmt1::G2-TAP-URA3*	ATGAGCAAGCGAGGCGGCAGAGATTCTGCT
YMW033	1	*HMT1::GCNpm*	**MATa** *his3Δ1 leu2Δ0 met15Δ0 hmt1::GCNpm-TAP-URA3*	ATGAGCACGGCAGCAGTAGCAGATTCTGCT
YMW034	2	*HMT1::GCNpm*	**MATa** *his3Δ1 leu2Δ0 met15Δ0 hmt1::GCNpm-TAP-URA3*	ATGAGCACGGCAGCAGTAGCAGATTCTGCT
YMW035	3	*HMT1::GCNpm*	**MATa** *his3Δ1 leu2Δ0 met15Δ0 hmt1::GCNpm-TAP-URA3*	ATGAGCACGGCAGCAGTAGCAGATTCTGCT

YMW0 36	4	*HMT1::GCNpm*	**MATa** *his3*Δ1 *leu2*Δ0 *met15*Δ0 *hmt1::GCNpm-TAP-URA3*	ATGAGCACGGCAGCA GTAGCAGATTCTGCT
YMW0 37	2	*HMT1::C1*	**MATa** *his3*Δ1 *leu2*Δ0 *met15*Δ0 *hmt1::C1-TAP-URA3*	ATGAGCCAGCAGCTG CTGAAAGATTCTGCT
YMW0 38	3	*HMT1::C1*	**MATa** *his3*Δ1 *leu2*Δ0 *met15*Δ0 *hmt1::C1-TAP-URA3*	ATGAGCCAGCAGCTG CTGAAAGATTCTGCT
YMW0 39	4	*HMT1::C1*	**MATa** *his3*Δ1 *leu2*Δ0 *met15*Δ0 *hmt1::C1-TAP-URA3*	ATGAGCCAGCAGCTG CTGAAAGATTCTGCT
YMW0 40	7	*HMT1::C1*	**MATa** *his3*Δ1 *leu2*Δ0 *met15*Δ0 *hmt1::C1-TAP-URA3*	ATGAGCCAGCAGCTG CTGAAAGATTCTGCT
