# Supplementary material for: GCN sensitive protein translation in yeast
Source: PLoS One. 2020 Sep 18;15(9):e0233197. doi: 10.1371/journal.pone.0233197 (PMC7500604; doi:10.1371/journal.pone.0233197)
Supplement: S2 Table — (PDF) [file pone.0233197.s008.pdf]

S2 Table. Primers for ramp mutants.

Uracil (URA3) Insert for Histidine (HIS3MX6) Replacement

Primer Name	Description	Sequence (5'-3')
ADH_URA_Forward	Forward primer that anneals to *URA3* (18bp homology) to replace *HIS3MX6* in the TAP construct.	TATTGACCACACCTCTAC CGGCAGATCCGCTAGGG ATAACAGGGTAATATTTA CCTTTTTTGCGAGGC
URA_SKN7_Reverse	Reverse primer that anneals within *URA3* (22bp homology) to replace *HIS3MX6* in the TAP construct. (48bp homology to *SKN7* on 5' end)	ATACAGAATGTCCTCTGC TAACTTAGACGCAAGGCT ATTTGTAAAATTATCATTA CGACCGAGATTCCCG
URA_HMT1_Reverse	Reverse primer that anneals within *URA3* (22bp homology) to replace *HIS3MX6* in the TAP construct. (48bp homology to *HMT1* on 5' end)	TGTTTATTTGCTTTTCAAA TTTTTTTCTTTCTCCAGCA AACAAAAGTCATCATTAC GACCGAGATTCCCG

URA3 Insertion Verification

Primer Name	Description	Sequence (5'-3')
ADH_Verif_Forward	Forward primer that anneals to the ADH terminator that is part of the TAP tag construct.	GTCAGGTTGCTTTCTCAG G
URA_Verif_Reverse	Reverse primer that anneals within *URA3* (19bp homology).	CTTGGTGGTACGAACATC C

Kanamycin/Hygromycin Cassette Insertion

Primer Name	Description	Sequence (5'-3')
SKN7_Kan_Forward	Forward primer to make *Kan* insertion at bases 11-22 of *SKN7* for first step of CRISPR-Cas9. (18bp homology to *Kan,* 45 bp homology to *SKN7* on the 5' end)	TCCTATTTTTTCGTTGCTT ACTTTTGATATCCACTAT GAGCTTTTCGTACGCTGC AGGTCGAC
SKN7_Kan_Reverse	Reverse primer to make *Kan* insertion at bases 11-22 of *SKN7* for first step of CRISPR-Cas9. (18bp homology to *Kan,* 45 bp homology to *SKN7* on the 5' end)	CGGTGGTGTTATTATTGC TATCGCCGGTGGTTTTAT TGACGTTGCATCGATGAA TTCGAGCTCG
HMT1_Kan_Forward	Forward primer to make *Kan* insertion at bases 8-22 of *HMT1* for first step of CRISPR-Cas9. (18bp homology to *Kan,* 45 bp homology to *HMT1* on the 5' end)	AAAAGAGTTAGAACCGAC AAATTCATCCAAAGAAAA TAATGAGCACGTACGCT GCAGGTCGAC

HMT1_Kan_Reverse	Reverse primer to make Kan insertion at bases 8-22 of *HMT1* for first step of CRISPR-Cas9. (18bp homology to Kan, 45 bp homology to *HMT1* on the 5' end)	GCTGTTCGCTTTCACTTAGCTTGGTTTTTTCTGTAGCAGAATCTTATCGATGAATTCGAGCTCG

Healing Fragments

**Primer Name**	**Description**	**Sequence (5'-3')**
SKN7 Primers		
SKN7_healing_ forward	*SKN7::G2*. Forward primer to make a healing fragment that contains G2 substitutions at four nucleotides in the *SKN7* gene (44 bp of homology on 5' end before mutation region)	CCTATTTTTTCGTTGCTTACTTTTGATATCCACTATGAGCTTTTGCAGCAGAAGTAGCAA
SKN7_healing_ reverse	*SKN7::G2*. Reverse primer to make a healing fragment that contains G2 substitutions at four nucleotides in the *SKN7* gene (44 bp of homology on 5' end with *SKN7* after mutation)	GGTGGTGTTATTATTGCTATCGCCGGTGGTTTTATTGACGTTGCTACTTCTGCTGCAAAA
SKN7_G7_Forward	*SKN7::GCNpm*. Forward primer to make a healing fragment that contains nucleotide substitutions starting at position 7 of ORF.	TTCCTATTTTTTCGTTGCTTACTTTTGATATCCACTATGAGCGCCTCCACCATAGCAGCC
SKN7_G7_Reverse	*SKN7::GCNpm*. Reverse primer.	GGTGGTGTTATTATTGCTATCGCCGGTGGTTTTATTGACGTTGGCTGCTATGGTGGAGGC
SKN_S819_heal_Forward	*SKN7::A-rich*. Forward primer to make a healing fragment that contains nucleotide substitutions starting at position 7 of ORF.	CCTATTTTTTCGTTGCTTACTTTTGATATCCACTATGAGCAAAAAAAGCAAAAATAGCAA
SKN_S819_heal_Reverse	*SKN7::A-rich*. Reverse primer.	GTGTTATTATTGCTATCGCCGGTGGTTTTATTGACGTTGCTATTTTTGCTTTTTTTGCTC
SKN7_I7_Forward	*SKN7::GCNi*. Forward primer to make a healing fragment that inserts a four-codon cassette after position 7. (35bp of homology with reverse primer, 40bp homology with *SKN7)*	CCTATTTTTTCGTTGCTTACTTTTGATATCCACTATGAGCTTAGCAGCAGCCTTTTCCACCATAAATAGC
SKN7_I7_Reverse	*SKN7::GCNi*. Reverse primer to make a healing fragment that inserts a four-codon cassette after position 7. (35bp of homology with reverse primer, 40bp homology with *SKN7)*	GTGTTATTATTGCTATCGCCGGTGGTTTTATTGACGTTGCTATTTATGGTGGAAAAGGCTGCTGCTAAGCTCA

HMT1 Primers		
HMT_M1_Forward	*HMT1::GCNpm*. Forward primer to make a healing fragment that contains nucleotide substitutions starting at position 8 of ORF.	AAAGAGTTAGAACCGACA AATTCATCCAAAGAAAATA ATGAGCACGGCAGCAGTA GCAGA
HMT1_M1_Reverse	*HMT1::GCNpm*. Reverse primer.	TGTTCGCTTTCACTTAGCT TGGTTTTTTCTGTAGCAG AATCTGCTACTGCTGCCG TGCTC
HMT1_G2_Forward	*HTM1::G2*. Forward primer to make a healing fragment that contains nucleotide substitutions starting at position 10 of ORF.	AAAAGAGTTAGAACCGAC AAATTCATCCAAAGAAAAT AATGAGCAAGCGAGGCG GCAGAG
HMT1_G2_Reverse	*HTM1::G2*. Reverse primer.	TGTTCGCTTTCACTTAGCT TGGTTTTTTCTGTAGCAG AATCTCTGCCGCCTCGCT TGCTC
HMT1_C2_Forward	*HMT1::C1*. Forward primer to make a healing fragment that contains nucleotide substitutions starting at position 7 of ORF.	AAAAGAGTTAGAACCGAC AAATTCATCCAAAGAAAAT AATGAGCCAGCAGCTGCT GAAAG
HMT1_C2_Reverse	*HMT1::C1*. Reverse primer.	GCTGTTCGCTTTCACTTA GCTTGGTTTTTTCTGTAG CAGAATCTTTCAGCAGCT GCTGGC

Verification of Successful Homologous Recombination

**Primer Name**	**Description**	**Sequence (5'-3')**
SKN7ann_verif_ forward	Forward verification primer that anneals 173 bases upstream of the mutation region.	GCTGCTTTTGTTTGGGTC
SKN7ann_verif_ reverse	Reverse verification primer that anneals 72 bases downstream of the mutation region.	CTCTGCAACAAGTCCATT C
SKN7ann_verif_ forward2	Forward verification primer that anneals 270 bases upstream of the mutation region.	TGAAAGTGCTTCCAGGAT CTG
SKN7ann_verif_ reverse2	Reverse verification primer that anneals 111 bases downstream of the mutation region.	GTTGTTTGGCTGCATCG
SKN7ann_seq_ forward	Sequencing primer that anneals 102 bases upstream of the mutation region.	AAAAAGCAACGAAATTGC TG
HMT1_verif_forward	Forward primer that anneals 145 bases prior to the mutation region.	AAATTTGGAAATGGCGG G

HMT1_verif_reverse	Reverse primer that anneals to *HMT1*, 72 bp downstream of the mutation region.	AAATTTGGAAATGGCGG G
HMT1_seq_forward	Sequencing forward primer that anneals within the verification fragments 145 bases before the mutation region.	TAGTTGAAGAGATGAGCT GC
