# Supplementary material for: GCN sensitive protein translation in yeast
Source: PLoS One. 2020 Sep 18;15(9):e0233197. doi: 10.1371/journal.pone.0233197 (PMC7500604; doi:10.1371/journal.pone.0233197)
Supplement: S3 Table — Normalized Translation Efficiency. Codon-specific normalized translation efficiency (nTE) scores for each mutant in SKN7 (A) and HMT1 (B). Green scores represent positive deviations from wildtype sequence, and red scores represent negative deviations from wildtype sequence. Normalized Translation Efficiency (nTE) scores provided a measure of codon bias (cAI) and tRNA availability (tAI). (PDF) [file pone.0233197.s009.pdf]

S3 Table. Normalized Translation Efficiency. Normalized Translation Efficiency. Codon-specific normalized translation efficiency (nTE) scores for each mutant in SKN7 (A) and HMT1 (B). Green scores represent positive deviations from wildtype sequence, and red scores represent negative deviations from wildtype sequence. Normalized Translation Efficiency (nTE) scores provided a measure of codon bias (cAI) and tRNA availability (tAI).

A	SKN7 Genotype				
Codon	*WT*	*G2*	*GCNi*	*GCNpm*	*A-rich*
1	0.261	0.261	0.261	0.261	0.261
2	0.119	0.119	0.119	0.119	0.119
3	0.096	0.096	0.147	0.31	0.094
4	0.292	0.513	0.171	0.292	0.094
5	0.318	0.119	0.171	0.318	0.119
6	0.069	0.265	0.31	0.069	0.094
7	0.072	0.035	0.096	0.171	0.072
8	0.119	0.119	0.292	0.31	0.119
9	0.216	0.216	0.318	0.216	0.216
10	0.421	0.421	0.069	0.421	0.421
11	0.072	0.072	0.072	0.072	0.072

B	HMT1 Genotype			
Codon	*WT*	*GCNpm*	*G2*	*C1*
1	0.261	0.261	0.261	0.261
2	0.119	0.119	0.119	0.119
3	0.263	0.166	0.263	0.181
4	0.131	0.171	0.00013	0.181
5	0.31	0.171	0.889	0.052
6	0.134	0.097	0.889	0.052
7	0.094	0.171	0.265	0.094
8	0.101	0.101	0.101	0.101
9	0.248	0.248	0.248	0.248
10	0.247	0.247	0.247	0.247
11	0.131	0.131	0.131	0.131
