# Supplementary material for: GCN sensitive protein translation in yeast
Source: PLoS One. 2020 Sep 18;15(9):e0233197. doi: 10.1371/journal.pone.0233197 (PMC7500604; doi:10.1371/journal.pone.0233197)
Supplement: S4 Table — BLOSUM scores for amino acid substitutions for point mutants of SKN7 and HMT1. Red letters represent amino acid deviations from wildtype protein sequence. BLOSUM62 scores are sums of each value obtained from the matrix for each amino acid substitution. (PDF) [file pone.0233197.s010.pdf]

S4 Table. BLOSUM assessment. BLOSUM scores for amino acid substitutions for point mutants of SKN7 and HMT1. Red letters represent amino acid deviations from wildtype protein sequence. BLOSUM62 scores are sums of each value obtained from the matrix for each amino acid substitution.

Ramp Mutant	Protein Sequence	BLOSUM62 Score (Relative to WT)
SKN7 WT	MSFSTINSNV	0
SKN7::GCNpm	MS**A**STI**AA**NV	-3
SKN7::G2	MSF**CSRS**SNV	-2
SKN7::A-rich	MS**KKSK**NSNV	-5
HMT1 WT	MSKTAVKDSA	0
HMT1::GCNpm	MS**TA**AV**A**DSA	-2
HMT1::G2	MSK**RGGR**DSA	-2
HMT1::C1	MS**QQLL**KDSA	0
