# Supplementary material for: GCN sensitive protein translation in yeast
Source: PLoS One. 2020 Sep 18;15(9):e0233197. doi: 10.1371/journal.pone.0233197 (PMC7500604; doi:10.1371/journal.pone.0233197)
Supplement: S5 Table — Comparison of anticodons with modifications. (PDF) [file pone.0233197.s011.pdf]

S5 Table. Ramp mutant anti-codons. Comparison of anticodons with modifications.

ORF ramp [1]	tRNA nt 34 modifications [2]	ΔI34 [3]
GAA GUU GCU anticodons<br>Phe Asn Ser<br>AUG AGC UUU UCC ACC AUA AAU AGC AAC *SKN7 WT* codons	Phe Gm34	
AUG AGC GCC UCC ACC AUA GCA GCC AAC ***SKN7::GCNpm***<br>Ala Ala Ala<br>IGC IGC IGC anticodons	Ala I34	+3
IGA IGU IAU GUU<br>Ser Thr Ile Asn<br>AUG AGC UUU UCC ACC AUA AAU AGC AAC *SKN7 WT*	Phe Gm34<br>Ser, Thr, Ile I34	
AUG AGC UUU UGC AGC AGA AGU AGC AAC ***SKN7::G2***<br>Cys Ser Arg Ser<br>GCA GCU UCU GCU	Arg mcm5U34	-3
GAA IGA IGU IAU<br>Phe Ser Thr Ile<br>AUG AGC UUU UCC ACC AUA AAU AGC AAC *SKN7 WT*	Phe Gm34<br>Ser, Thr, Ile I34	
AUG AGC AAA AAA AGC AAA AAU AGC AAC ***SKN7::A-rich***<br>Lys Lys Ser Lys<br>UUU UUU GCU UUU	Lys mcm5s2U34	-3
CUU UAC<br>UUU IGU IGC CAC UUU<br>Lys Thr Ala Val Lys<br>AUG AGC AAG ACA GCC GUG AAA GAU *HMT1 WT*	Lys mcm5s2U34<br>Val ncm5U (alt)<br>Thr, Ala I34	
AUG AGC ACG GCA GCA GUA GCA GAU ***HMT1::GCNpm***<br>Thr Ala Ala Val Ala<br>IGU IGC IGC IAC IGC<br>UAC	Val ncm5U34<br>Thr, Ala, Val I34	+3
UAC<br>IGU IGC CAC UUU<br>Thr Ala Val Lys<br>AUG AGC AAG ACA GCC GUG AAA GAU *HMT1 WT*	Lys mcm5s2U34<br>Val ncm5U (alt)<br>Thr, Ala I34	
AUG AGC AAG CGA GGC GGC AGA GAU ***HMT1::G2***<br>Arg Gly Gly Arg<br>IGC GCC GCC UCU	Arg mcm5U34<br>Arg I34	-1
CUU UAC<br>UUU IGU IGC CAC<br>Lys Thr Ala Val<br>AUG AGC AAG ACA GCC GUG AAA GAU *HMT1 WT*	Lys mcm5s2U34<br>Val ncm5U (alt)<br>Thr, Ala I34	
AUG AGC CAG CAG CUG CUG AAA GAU ***HMT1::C1***<br>Gln Gln Leu Leu<br>CUG CUG UAG UAG		-2

[1] Codons with ramp point mutations (red) are illustrated.
[2] tRNA nucleotide 34 modifications that are stress-regulated are illustrated in bold in column 1 and annotated in the column 2.
[3] This column shows the change in the number of Inosine 34 (I34) tRNAs in the mutated codons compared to WT.
