# Supplementary material for: GCN sensitive protein translation in yeast
Source: PLoS One. 2020 Sep 18;15(9):e0233197. doi: 10.1371/journal.pone.0233197 (PMC7500604; doi:10.1371/journal.pone.0233197)
Supplement: S2 File — Spectra for data in S1 File. (PDF) [file pone.0233197.s013.pdf]

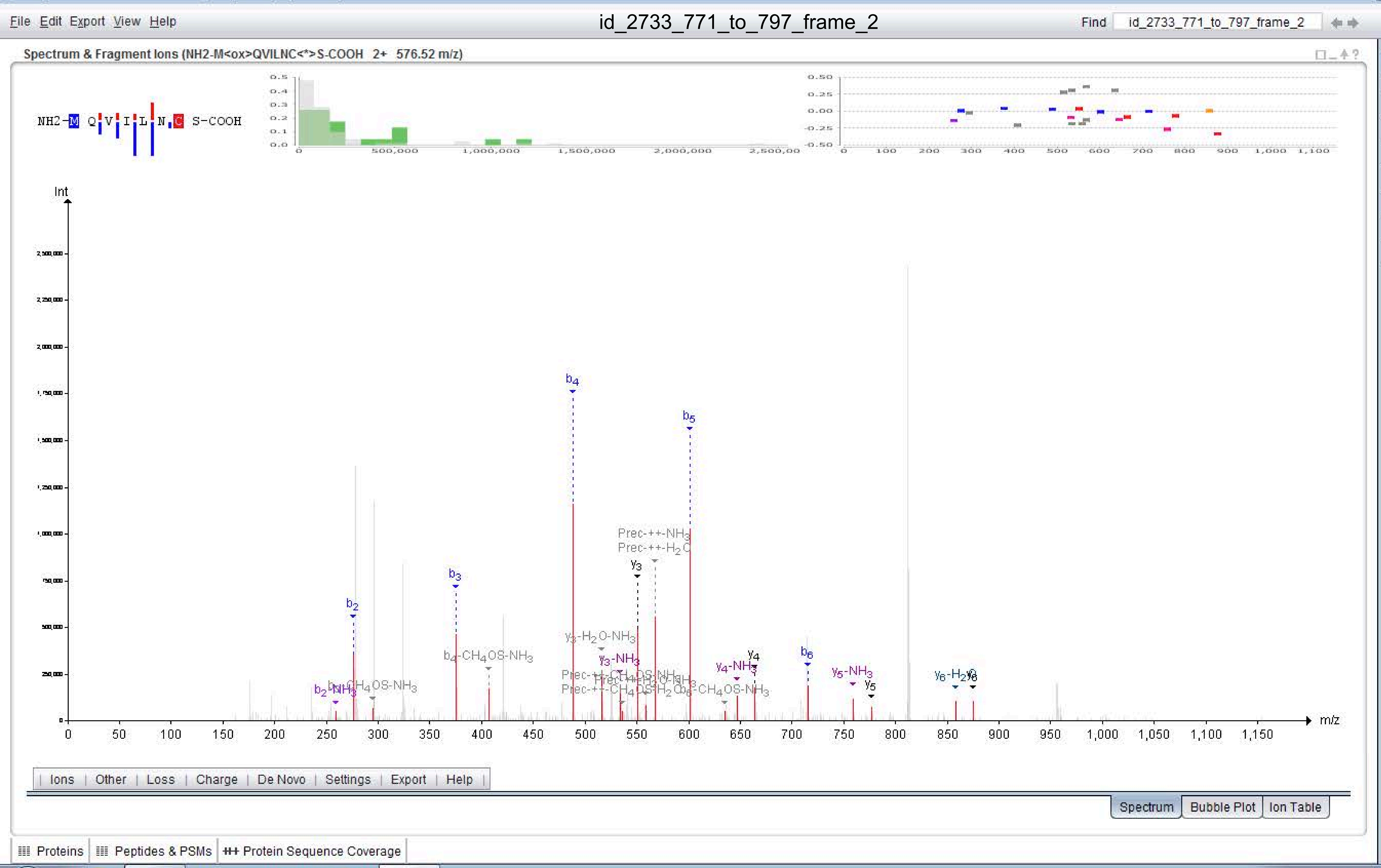
File Edit Export View Help
id_2733_771_to_797_frame_2
Find id_2733_771_to_797_frame_2
(1 of 1)
Spectrum & Fragment Ions (NH2-M<ox>QVILNC<*>S-COOH 2+ 576.52 m/z)
NH2-M Q V I L N C S-COOH
Int
m/z
b4
b5
b3
b2
y3
Prec-++-NH3
Prec-++-H2O
b4-CH4OS-NH3
y3-H2O-NH3
y3-NH3
y4-NH3
y4
b6
y5-NH3
y5
y6-H2O
y6
b2-NH3
Ions
Other
Loss
Charge
De Novo
Settings
Export
Help
Spectrum
Bubble Plot
Ion Table
Proteins
Peptides & PSMs
Protein Sequence Coverage
Overview
Spectrum IDs
Fractions
Modifications
3D Structures
Annotation
GO Analysis
Validation
QC Plots

S1

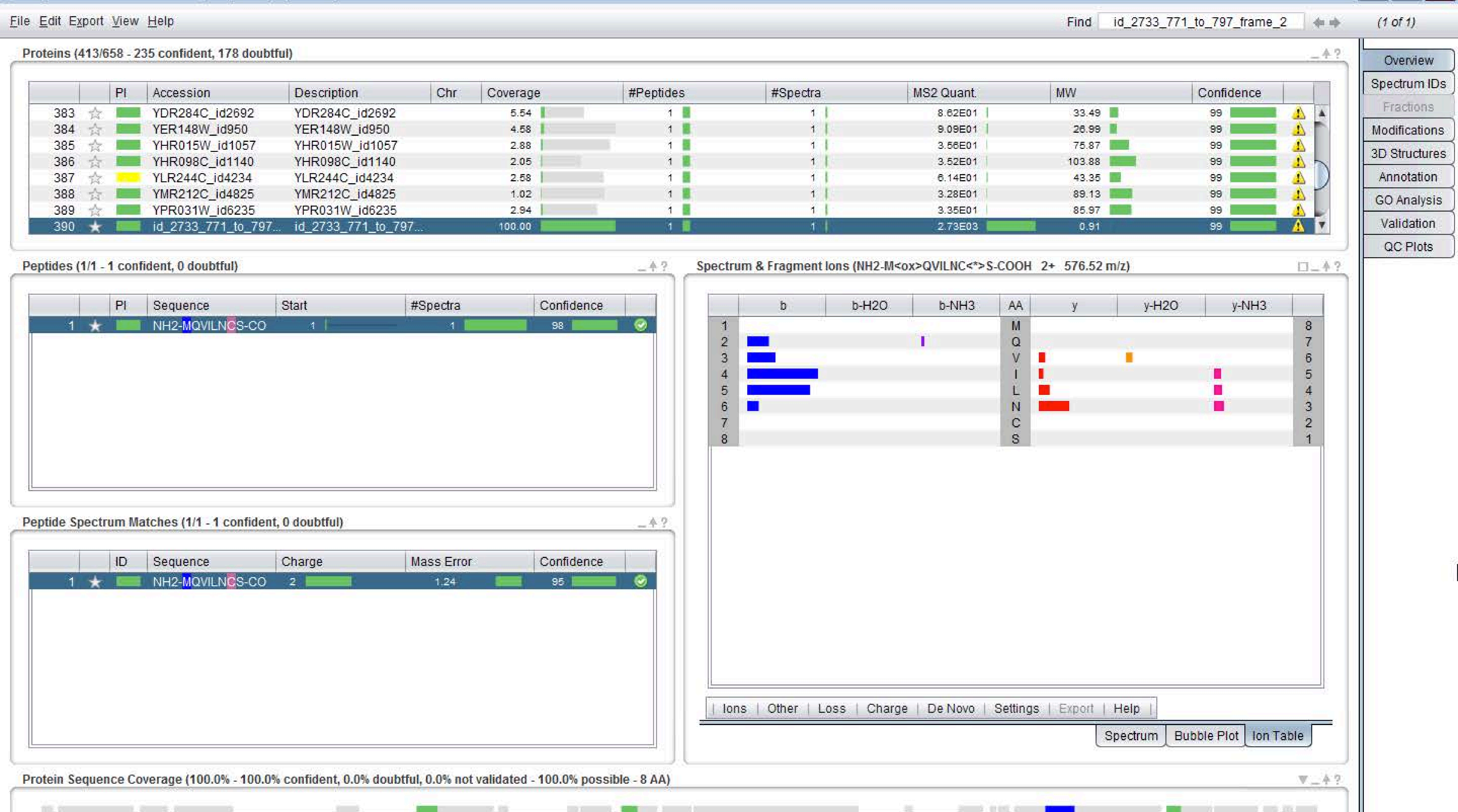
File Edit Export View Help
Find id_2733_771_to_797_frame_2
(1 of 1)
Proteins (413/658 - 235 confident, 178 doubtful)
PI Accession Description Chr Coverage #Peptides #Spectra MS2 Quant. MW Confidence
383 YDR284C_id2692 YDR284C_id2692 5.54 1 1 8.62E01 33.49 99
384 YER148W_id950 YER148W_id950 4.58 1 1 9.09E01 26.99 99
385 YHR015W_id1057 YHR015W_id1057 2.88 1 1 3.56E01 75.87 99
386 YHR098C_id1140 YHR098C_id1140 2.05 1 1 3.52E01 103.88 99
387 YLR244C_id4234 YLR244C_id4234 2.58 1 1 6.14E01 43.35 99
388 YMR212C_id4825 YMR212C_id4825 1.02 1 1 3.28E01 89.13 99
389 YPR031W_id6235 YPR031W_id6235 2.94 1 1 3.35E01 85.97 99
390 id_2733_771_to_797... id_2733_771_to_797... 100.00 1 1 2.73E03 0.91 99
Overview
Spectrum IDs
Fractions
Modifications
3D Structures
Annotation
GO Analysis
Validation
QC Plots
Peptides (1/1 - 1 confident, 0 doubtful)
PI Sequence Start #Spectra Confidence
1 NH2-MQVILNCS-CO 1 1 98
Spectrum & Fragment Ions (NH2-M<ox>QVILNC<*>S-COOH 2+ 576.52 m/z)
b b-H2O b-NH3 AA y y-H2O y-NH3
1 M 8
2 Q 7
3 V 6
4 I 5
5 L 4
6 N 3
7 C 2
8 S 1
Peptide Spectrum Matches (1/1 - 1 confident, 0 doubtful)
ID Sequence Charge Mass Error Confidence
1 NH2-MQVILNCS-CO 2 1.24 95
Ions Other Loss Charge De Novo Settings Export Help
Spectrum Bubble Plot Ion Table
Protein Sequence Coverage (100.0% - 100.0% confident, 0.0% doubtful, 0.0% not validated - 100.0% possible - 8 AA)

S2

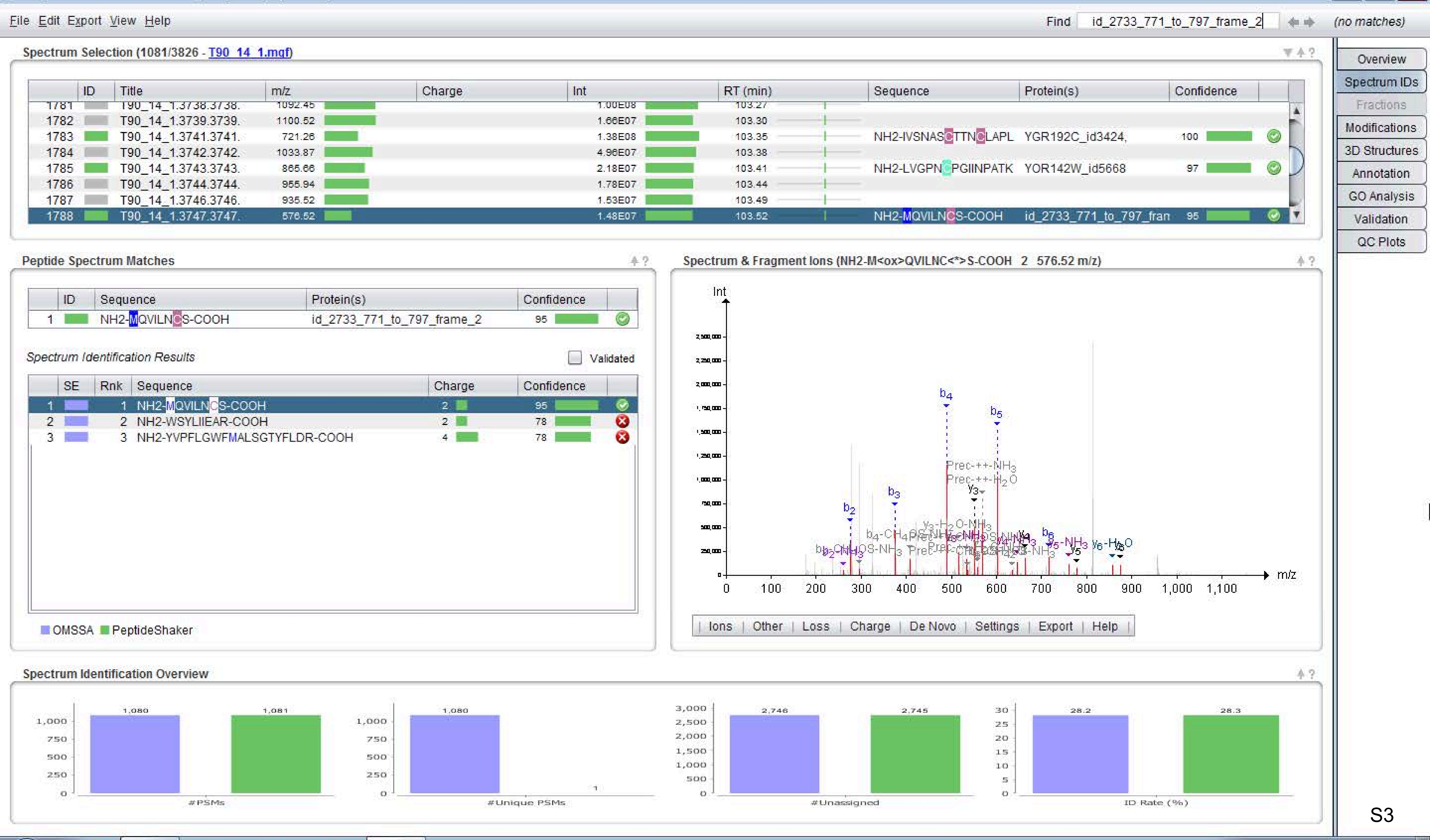
File Edit Export View Help
Find id_2733_771_to_797_frame_2
(no matches)
Spectrum Selection (1081/3826 - T90_14_1.mgf)
ID Title m/z Charge Int RT (min) Sequence Protein(s) Confidence
1782 T90_14_1.3739.3739. 1100.52 1.66E07 103.30
1783 T90_14_1.3741.3741. 721.26 1.38E08 103.35 NH2-IVSNASCTTNCLAPL YGR192C_id3424, 100
1784 T90_14_1.3742.3742. 1033.87 4.96E07 103.38
1785 T90_14_1.3743.3743. 865.66 2.18E07 103.41 NH2-LVGPNCPGIINPATK YOR142W_id5668 97
1786 T90_14_1.3744.3744. 955.94 1.78E07 103.44
1787 T90_14_1.3746.3746. 935.52 1.53E07 103.49
1788 T90_14_1.3747.3747. 576.52 1.48E07 103.52 NH2-MQVILNCS-COOH id_2733_771_to_797_fran 95
Overview
Spectrum IDs
Fractions
Modifications
3D Structures
Annotation
GO Analysis
Validation
QC Plots
Peptide Spectrum Matches
ID Sequence Protein(s) Confidence
1 NH2-MQVILNCS-COOH id_2733_771_to_797_frame_2 95
Spectrum Identification Results
Validated
SE Rnk Sequence Charge Confidence
1 1 NH2-MQVILNCS-COOH 2 95
2 2 NH2-WSYLIIEAR-COOH 2 78
3 3 NH2-YVPFLGWFMALSGTYFLDR-COOH 4 78
OMSSA PeptideShaker
Spectrum & Fragment Ions (NH2-M<ox>QVILNC<*>S-COOH 2 576.52 m/z)
Int
m/z
Ions Other Loss Charge De Novo Settings Export Help
Spectrum Identification Overview
#PSMs
#Unique PSMs
#Unassigned
ID Rate (%)

S3

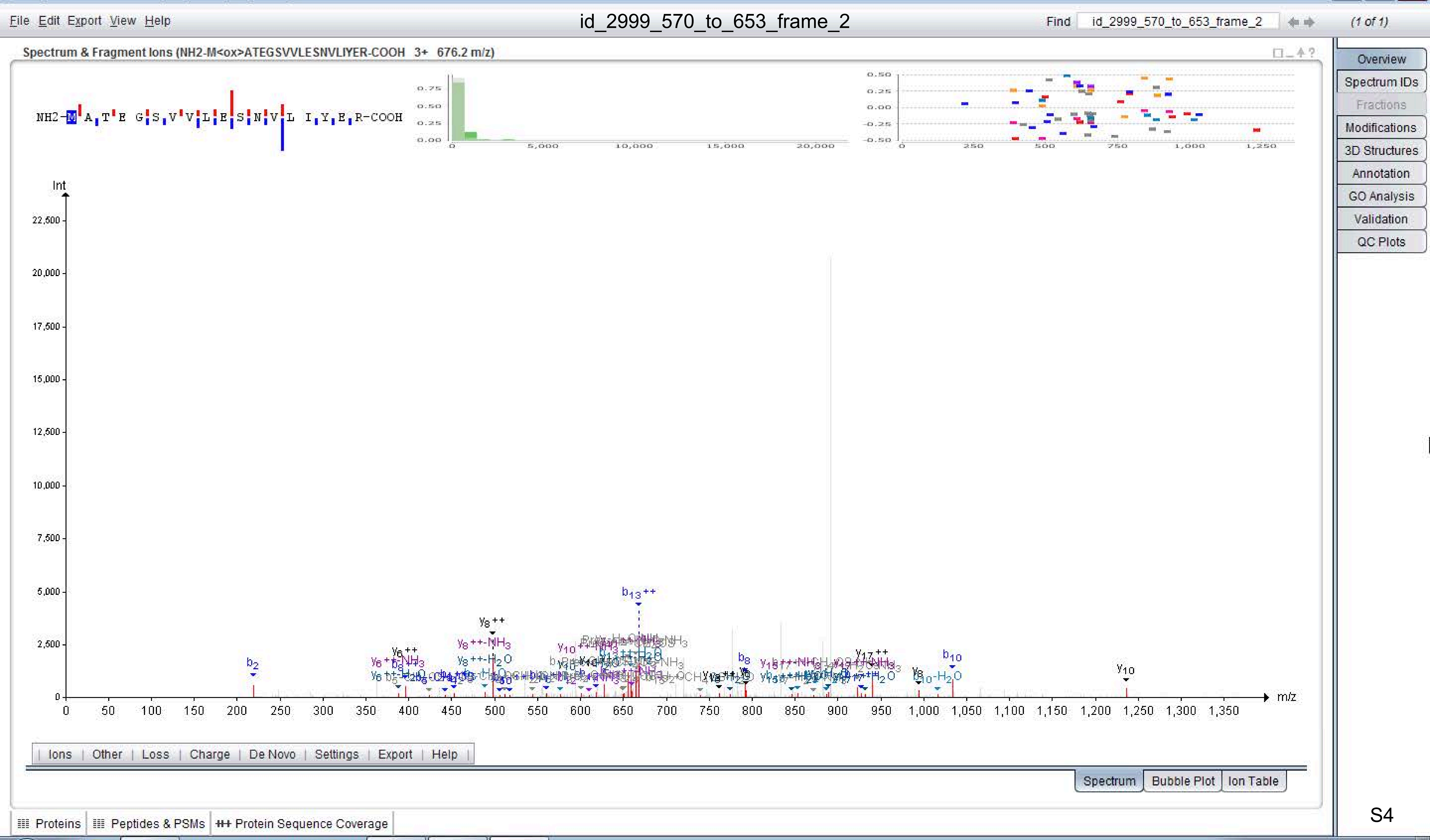
File Edit Export View Help
id_2999_570_to_653_frame_2
Find id_2999_570_to_653_frame_2
(1 of 1)
Spectrum & Fragment Ions (NH2-M<ox>ATEGSVVLESNVLIYER-COOH 3+ 676.2 m/z)
NH2-M A T E G S V V L E S N V L I Y E R-COOH
Overview
Spectrum IDs
Fractions
Modifications
3D Structures
Annotation
GO Analysis
Validation
QC Plots
Int
m/z
b13++
y8++
y8++-NH3
y8++-H2O
y6++
b2
b8
b10
b10-H2O
y10
y17++
Ions
Other
Loss
Charge
De Novo
Settings
Export
Help
Spectrum
Bubble Plot
Ion Table
Proteins
Peptides & PSMs
Protein Sequence Coverage

S4

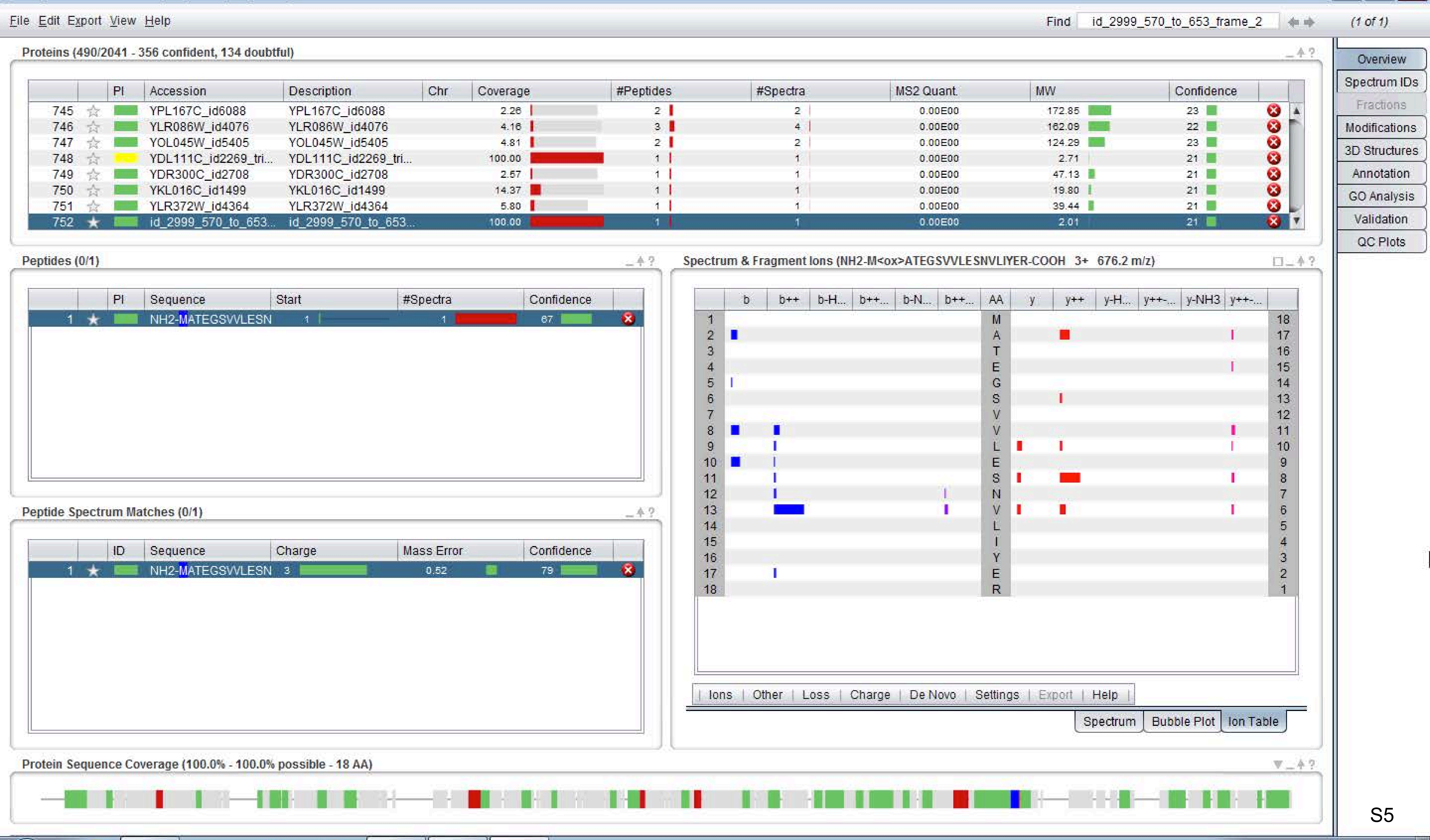
File Edit Export View Help
Find id_2999_570_to_653_frame_2
(1 of 1)
Proteins (490/2041 - 356 confident, 134 doubtful)
PI Accession Description Chr Coverage #Peptides #Spectra MS2 Quant. MW Confidence
745 YPL167C_id6088 YPL167C_id6088 2.26 2 2 0.00E00 172.85 23
746 YLR086W_id4076 YLR086W_id4076 4.16 3 4 0.00E00 162.09 22
747 YOL045W_id5405 YOL045W_id5405 4.81 2 2 0.00E00 124.29 23
748 YDL111C_id2269_tri... YDL111C_id2269_tri... 100.00 1 1 0.00E00 2.71 21
749 YDR300C_id2708 YDR300C_id2708 2.57 1 1 0.00E00 47.13 21
750 YKL016C_id1499 YKL016C_id1499 14.37 1 1 0.00E00 19.80 21
751 YLR372W_id4364 YLR372W_id4364 5.80 1 1 0.00E00 39.44 21
752 id_2999_570_to_653... id_2999_570_to_653... 100.00 1 1 0.00E00 2.01 21
Overview
Spectrum IDs
Fractions
Modifications
3D Structures
Annotation
GO Analysis
Validation
QC Plots
Peptides (0/1)
PI Sequence Start #Spectra Confidence
1 NH2-MATEGSVVLESN 1 1 67
Spectrum & Fragment Ions (NH2-M<ox>ATEGSVVLESNVLIYER-COOH 3+ 676.2 m/z)
b b++ b-H... b++... b-N... b++... AA y y++ y-H... y++-... y-NH3 y++-...
M A T E G S V V L E S N V L I Y E R
Peptide Spectrum Matches (0/1)
ID Sequence Charge Mass Error Confidence
1 NH2-MATEGSVVLESN 3 0.52 79
Ions Other Loss Charge De Novo Settings Export Help
Spectrum Bubble Plot Ion Table
Protein Sequence Coverage (100.0% - 100.0% possible - 18 AA)

S5

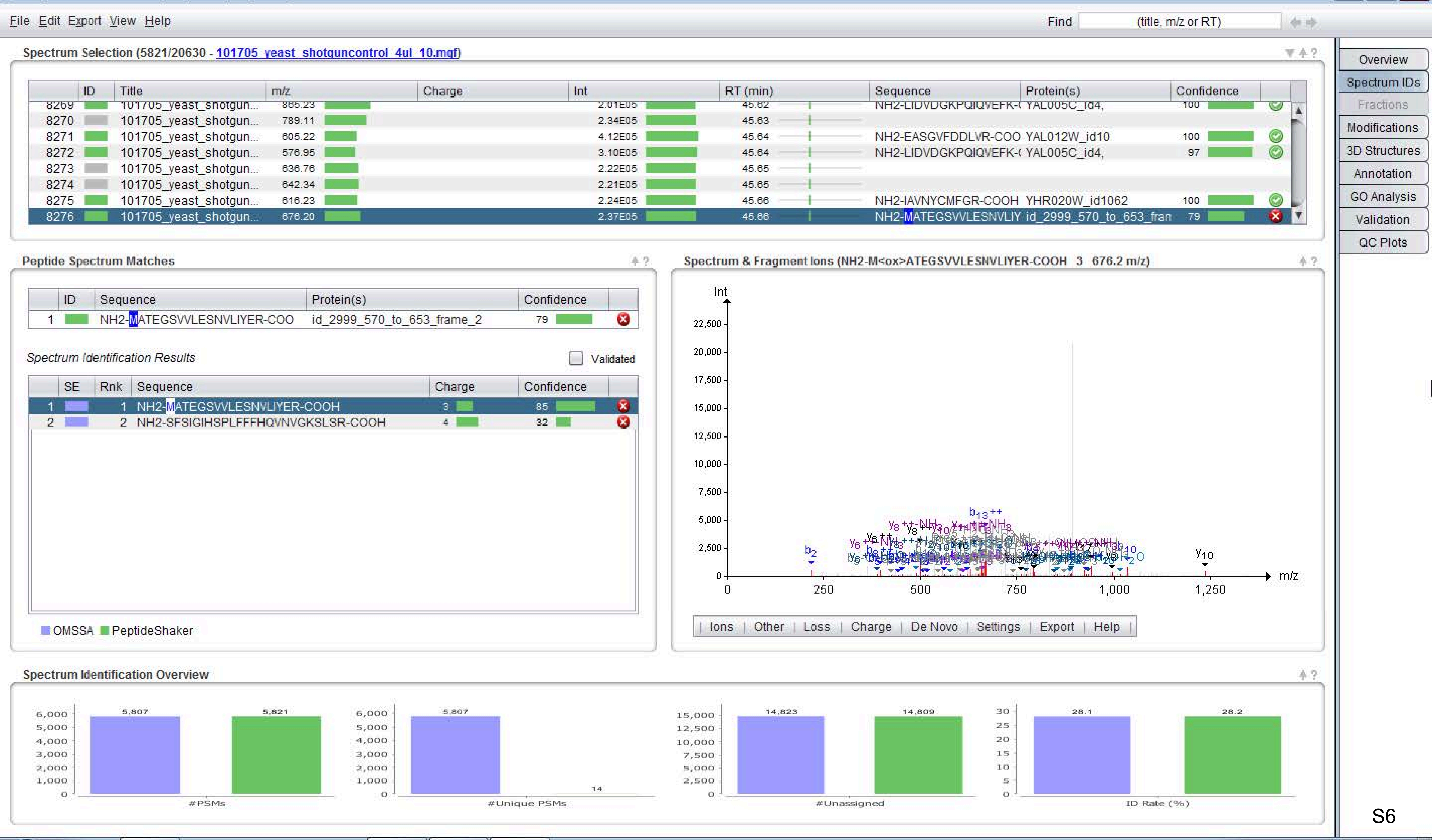
File Edit Export View Help
Find (title, m/z or RT)
Spectrum Selection (5821/20630 - 101705_yeast_shotguncontrol_4ul_10.mgf)
ID Title m/z Charge Int RT (min) Sequence Protein(s) Confidence
8270 101705_yeast_shotgun... 789.11 2.34E05 45.63
8271 101705_yeast_shotgun... 605.22 4.12E05 45.64 NH2-EASGVFDDLVR-COO YAL012W_id10 100
8272 101705_yeast_shotgun... 576.95 3.10E05 45.64 NH2-LIDVDGKPQIQVEFK-(YAL005C_id4, 97
8273 101705_yeast_shotgun... 636.76 2.22E05 45.65
8274 101705_yeast_shotgun... 642.34 2.21E05 45.65
8275 101705_yeast_shotgun... 616.23 2.24E05 45.66 NH2-IAVNYCMFGR-COOH YHR020W_id1062 100
8276 101705_yeast_shotgun... 676.20 2.37E05 45.66 NH2-MATEGSVVLESNVLIY id_2999_570_to_653_fran 79
Overview
Spectrum IDs
Fractions
Modifications
3D Structures
Annotation
GO Analysis
Validation
QC Plots
Peptide Spectrum Matches
ID Sequence Protein(s) Confidence
1 NH2-MATEGSVVLESNVLIYER-COO id_2999_570_to_653_frame_2 79
Spectrum Identification Results
Validated
SE Rnk Sequence Charge Confidence
1 1 NH2-MATEGSVVLESNVLIYER-COOH 3 85
2 2 NH2-SFSIGIHSPLFFFHQVNVGKSLSR-COOH 4 32
OMSSA PeptideShaker
Spectrum & Fragment Ions (NH2-M<ox>ATEGSVVLESNVLIYER-COOH 3 676.2 m/z)
Int
m/z
Ions Other Loss Charge De Novo Settings Export Help
Spectrum Identification Overview
5,807 5,821
#PSMs
5,807 14
#Unique PSMs
14,823 14,809
#Unassigned
28.1 28.2
ID Rate (%)

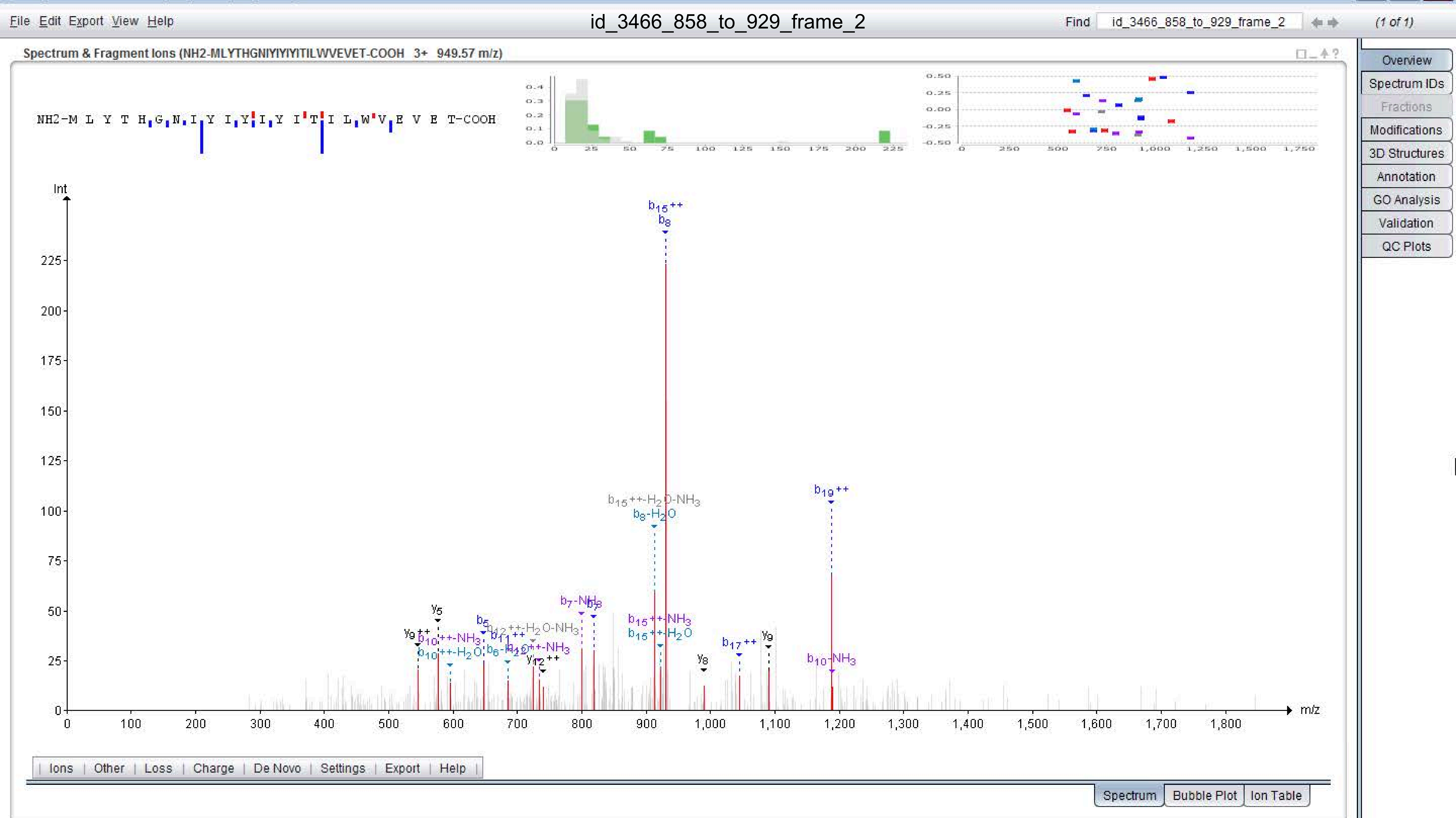
File Edit Export View Help
id_3466_858_to_929_frame_2
Find id_3466_858_to_929_frame_2
(1 of 1)
Spectrum & Fragment Ions (NH2-MLYTHGNIYIYIYITILWVEVET-COOH 3+ 949.57 m/z)
NH2-M L Y T H G N I Y I Y I Y I T I L W V E V E T-COOH
Overview
Spectrum IDs
Fractions
Modifications
3D Structures
Annotation
GO Analysis
Validation
QC Plots
Int
m/z
b15++
b8
b19++
b15++-H2O-NH3
b8-H2O
b7-NH3
b7
y5
b5
y9++
b10++-NH3
b10++-H2O
b11++
b6-H2O
b12++-NH3
y12++
b15++-NH3
b15++-H2O
b17++
y9
y8
b10-NH3
Ions | Other | Loss | Charge | De Novo | Settings | Export | Help
Spectrum
Bubble Plot
Ion Table
Proteins
Peptides & PSMs
Protein Sequence Coverage

S7

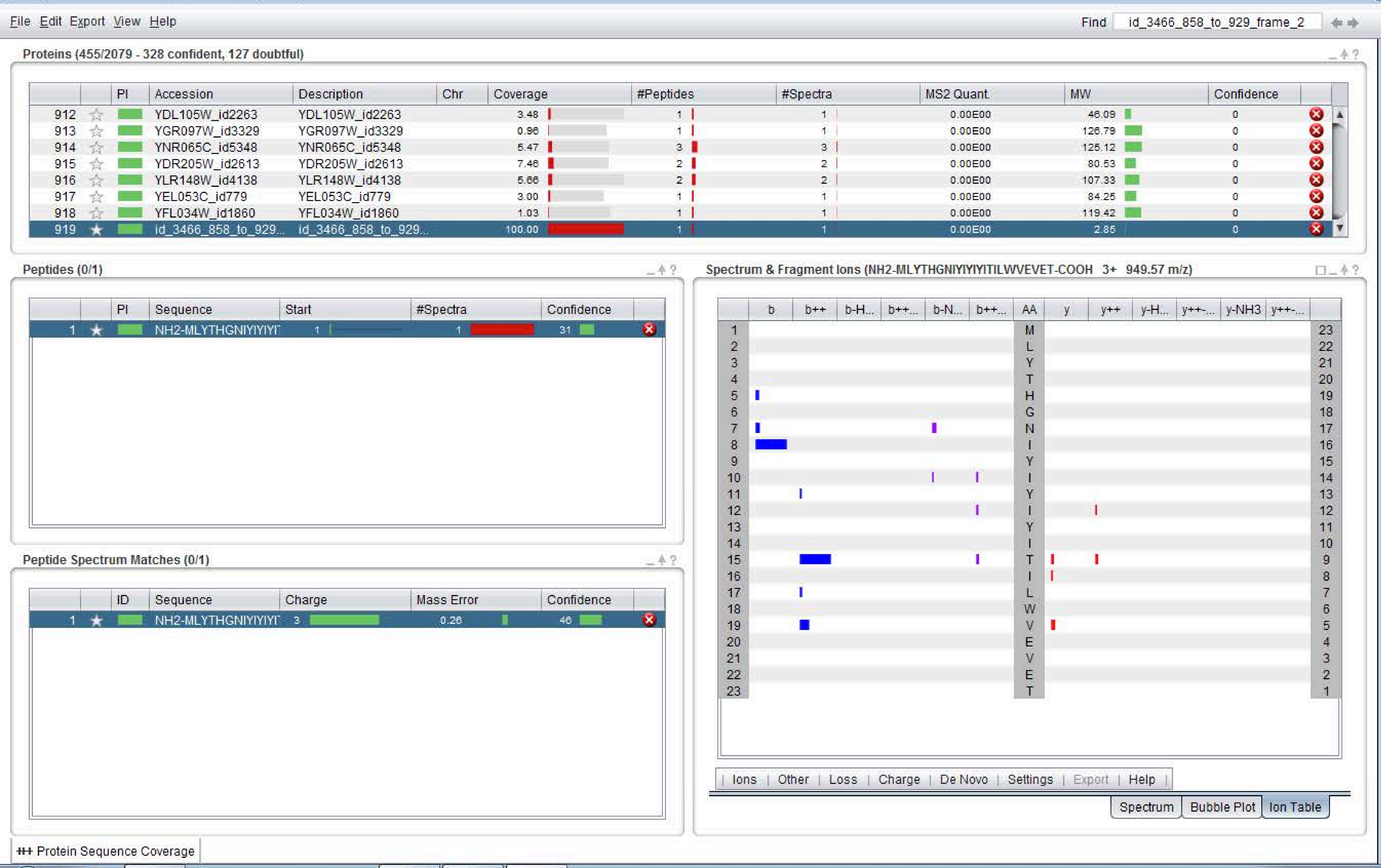
File Edit Export View Help
Find id_3466_858_to_929_frame_2
(1 of 1)
Proteins (455/2079 - 328 confident, 127 doubtful)
PI Accession Description Chr Coverage #Peptides #Spectra MS2 Quant. MW Confidence
912 YDL105W_id2263 YDL105W_id2263 3.48 1 1 0.00E00 46.09 0
913 YGR097W_id3329 YGR097W_id3329 0.96 1 1 0.00E00 126.79 0
914 YNR065C_id5348 YNR065C_id5348 5.47 3 3 0.00E00 125.12 0
915 YDR205W_id2613 YDR205W_id2613 7.46 2 2 0.00E00 80.53 0
916 YLR148W_id4138 YLR148W_id4138 5.66 2 2 0.00E00 107.33 0
917 YEL053C_id779 YEL053C_id779 3.00 1 1 0.00E00 84.25 0
918 YFL034W_id1860 YFL034W_id1860 1.03 1 1 0.00E00 119.42 0
919 id_3466_858_to_929... id_3466_858_to_929... 100.00 1 1 0.00E00 2.85 0
Overview
Spectrum IDs
Fractions
Modifications
3D Structures
Annotation
GO Analysis
Validation
QC Plots
Peptides (0/1)
PI Sequence Start #Spectra Confidence
1 NH2-MLYTHGNIYIYIYI 1 1 31
Spectrum & Fragment Ions (NH2-MLYTHGNIYIYIYITILWVEVET-COOH 3+ 949.57 m/z)
b b++ b-H... b++... b-N... b++... AA y y++ y-H... y++-... y-NH3 y++-...
1 M 23
2 L 22
3 Y 21
4 T 20
5 H 19
6 G 18
7 N 17
8 I 16
9 Y 15
10 I 14
11 Y 13
12 I 12
13 Y 11
14 I 10
15 T 9
16 I 8
17 L 7
18 W 6
19 V 5
20 E 4
21 V 3
22 E 2
23 T 1
Peptide Spectrum Matches (0/1)
ID Sequence Charge Mass Error Confidence
1 NH2-MLYTHGNIYIYIYI 3 0.26 46
Ions Other Loss Charge De Novo Settings Export Help
Spectrum Bubble Plot Ion Table
Protein Sequence Coverage

S8

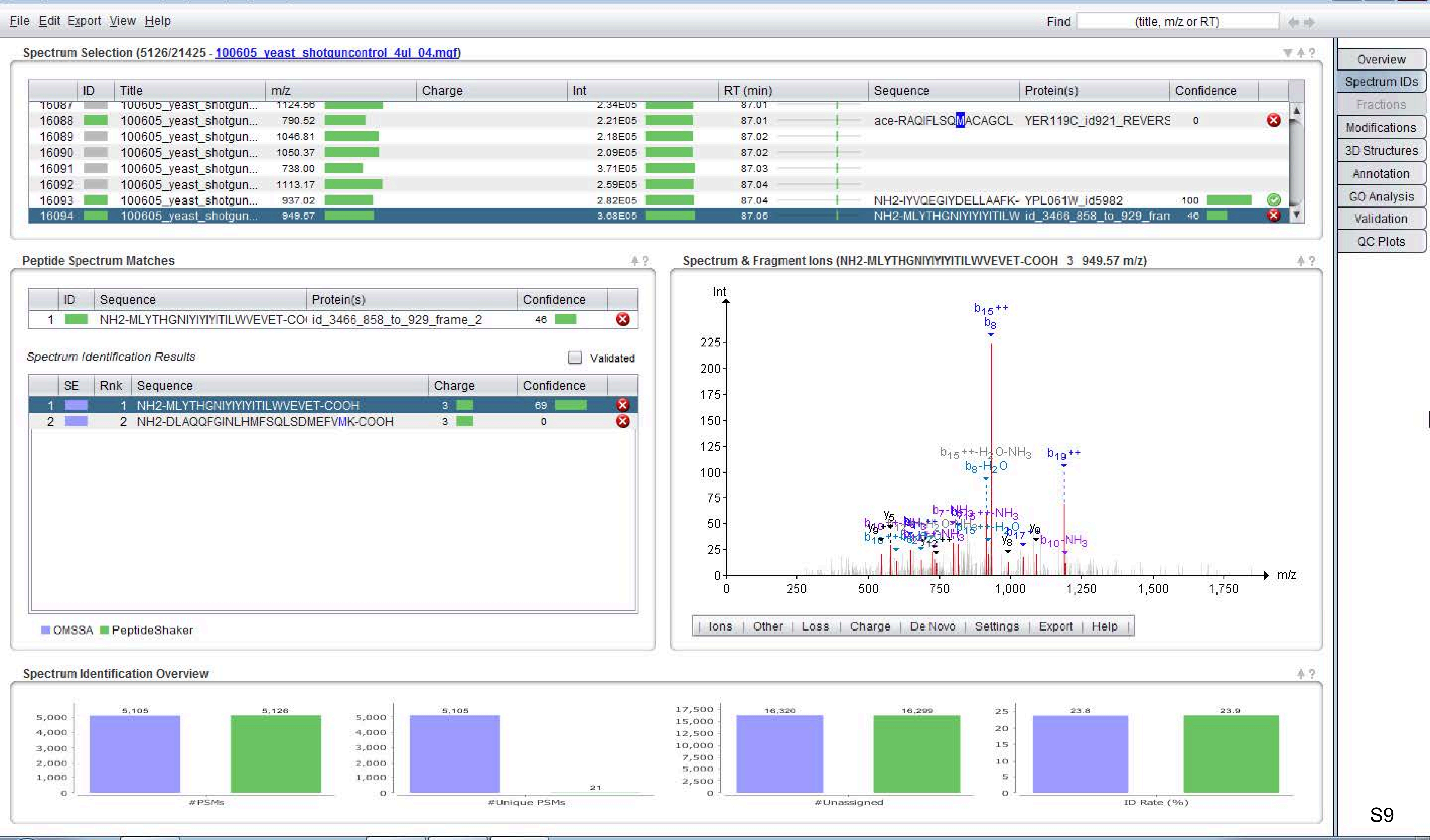
File Edit Export View Help
Find (title, m/z or RT)
Spectrum Selection (5126/21425 - 100605_yeast_shotguncontrol_4ul_04.mgf)
ID Title m/z Charge Int RT (min) Sequence Protein(s) Confidence
16088 100605_yeast_shotgun... 790.52 2.21E05 87.01 ace-RAQIFLSQMACAGCL YER119C_id921_REVERS 0
16089 100605_yeast_shotgun... 1046.81 2.18E05 87.02
16090 100605_yeast_shotgun... 1050.37 2.09E05 87.02
16091 100605_yeast_shotgun... 738.00 3.71E05 87.03
16092 100605_yeast_shotgun... 1113.17 2.59E05 87.04
16093 100605_yeast_shotgun... 937.02 2.82E05 87.04 NH2-IYVQEGIYDELLAAFK- YPL061W_id5982 100
16094 100605_yeast_shotgun... 949.57 3.68E05 87.05 NH2-MLYTHGNIYIYIYITILW id_3466_858_to_929_fran 46
Overview
Spectrum IDs
Fractions
Modifications
3D Structures
Annotation
GO Analysis
Validation
QC Plots
Peptide Spectrum Matches
ID Sequence Protein(s) Confidence
1 NH2-MLYTHGNIYIYIYITILWVEVET-CO id_3466_858_to_929_frame_2 46
Spectrum Identification Results
Validated
SE Rnk Sequence Charge Confidence
1 1 NH2-MLYTHGNIYIYIYITILWVEVET-COOH 3 69
2 2 NH2-DLAQQFGINLHMFSQLSDMEFVMK-COOH 3 0
OMSSA PeptideShaker
Spectrum & Fragment Ions (NH2-MLYTHGNIYIYIYITILWVEVET-COOH 3 949.57 m/z)
Int
m/z
Ions | Other | Loss | Charge | De Novo | Settings | Export | Help
Spectrum Identification Overview
#PSMs
#Unique PSMs
#Unassigned
ID Rate (%)

S9

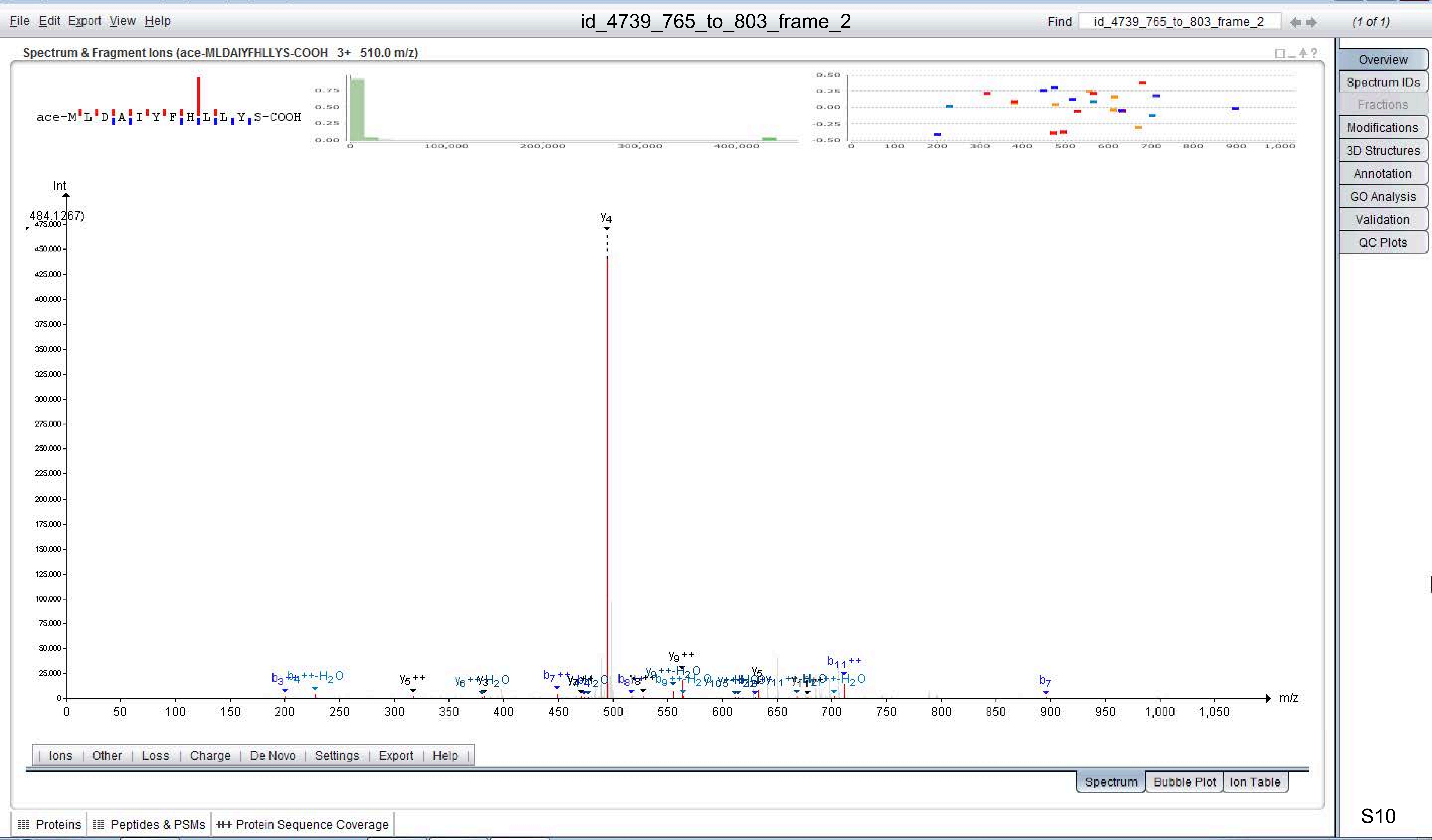
File Edit Export View Help
id_4739_765_to_803_frame_2
Find id_4739_765_to_803_frame_2
(1 of 1)
Spectrum & Fragment Ions (ace-MLDAIYFHLLYS-COOH 3+ 510.0 m/z)
ace-M L D A I Y F H L L Y S-COOH
Overview
Spectrum IDs
Fractions
Modifications
3D Structures
Annotation
GO Analysis
Validation
QC Plots
Int
m/z
y4
Ions | Other | Loss | Charge | De Novo | Settings | Export | Help
Spectrum
Bubble Plot
Ion Table
Proteins
Peptides & PSMs
Protein Sequence Coverage

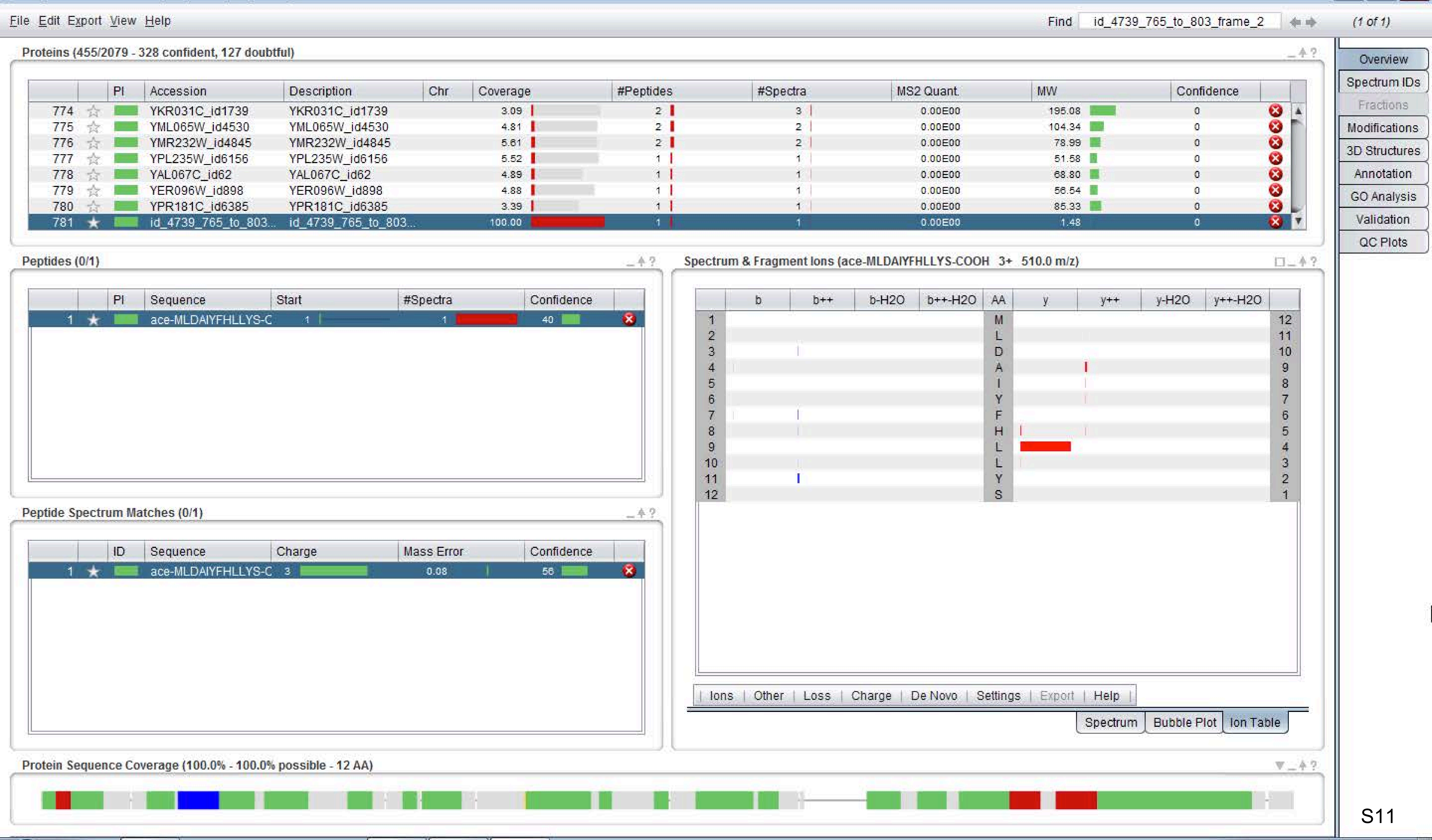
File Edit Export View Help
Find id_4739_765_to_803_frame_2
(1 of 1)
Proteins (455/2079 - 328 confident, 127 doubtful)
PI Accession Description Chr Coverage #Peptides #Spectra MS2 Quant. MW Confidence
774 YKR031C_id1739 YKR031C_id1739 3.09 2 3 0.00E00 195.08 0
775 YML065W_id4530 YML065W_id4530 4.81 2 2 0.00E00 104.34 0
776 YMR232W_id4845 YMR232W_id4845 5.61 2 2 0.00E00 78.99 0
777 YPL235W_id6156 YPL235W_id6156 5.52 1 1 0.00E00 51.58 0
778 YAL067C_id62 YAL067C_id62 4.89 1 1 0.00E00 68.80 0
779 YER096W_id898 YER096W_id898 4.88 1 1 0.00E00 56.54 0
780 YPR181C_id6385 YPR181C_id6385 3.39 1 1 0.00E00 85.33 0
781 id_4739_765_to_803... id_4739_765_to_803... 100.00 1 1 0.00E00 1.48 0
Overview
Spectrum IDs
Fractions
Modifications
3D Structures
Annotation
GO Analysis
Validation
QC Plots
Peptides (0/1)
PI Sequence Start #Spectra Confidence
1 ace-MLDAIYFHLLYS-C 1 1 40
Spectrum & Fragment Ions (ace-MLDAIYFHLLYS-COOH 3+ 510.0 m/z)
b b++ b-H2O b++-H2O AA y y++ y-H2O y++-H2O
1 M 12
2 L 11
3 D 10
4 A 9
5 I 8
6 Y 7
7 F 6
8 H 5
9 L 4
10 L 3
11 Y 2
12 S 1
Peptide Spectrum Matches (0/1)
ID Sequence Charge Mass Error Confidence
1 ace-MLDAIYFHLLYS-C 3 0.08 56
Ions Other Loss Charge De Novo Settings Export Help
Spectrum Bubble Plot Ion Table
Protein Sequence Coverage (100.0% - 100.0% possible - 12 AA)

S11

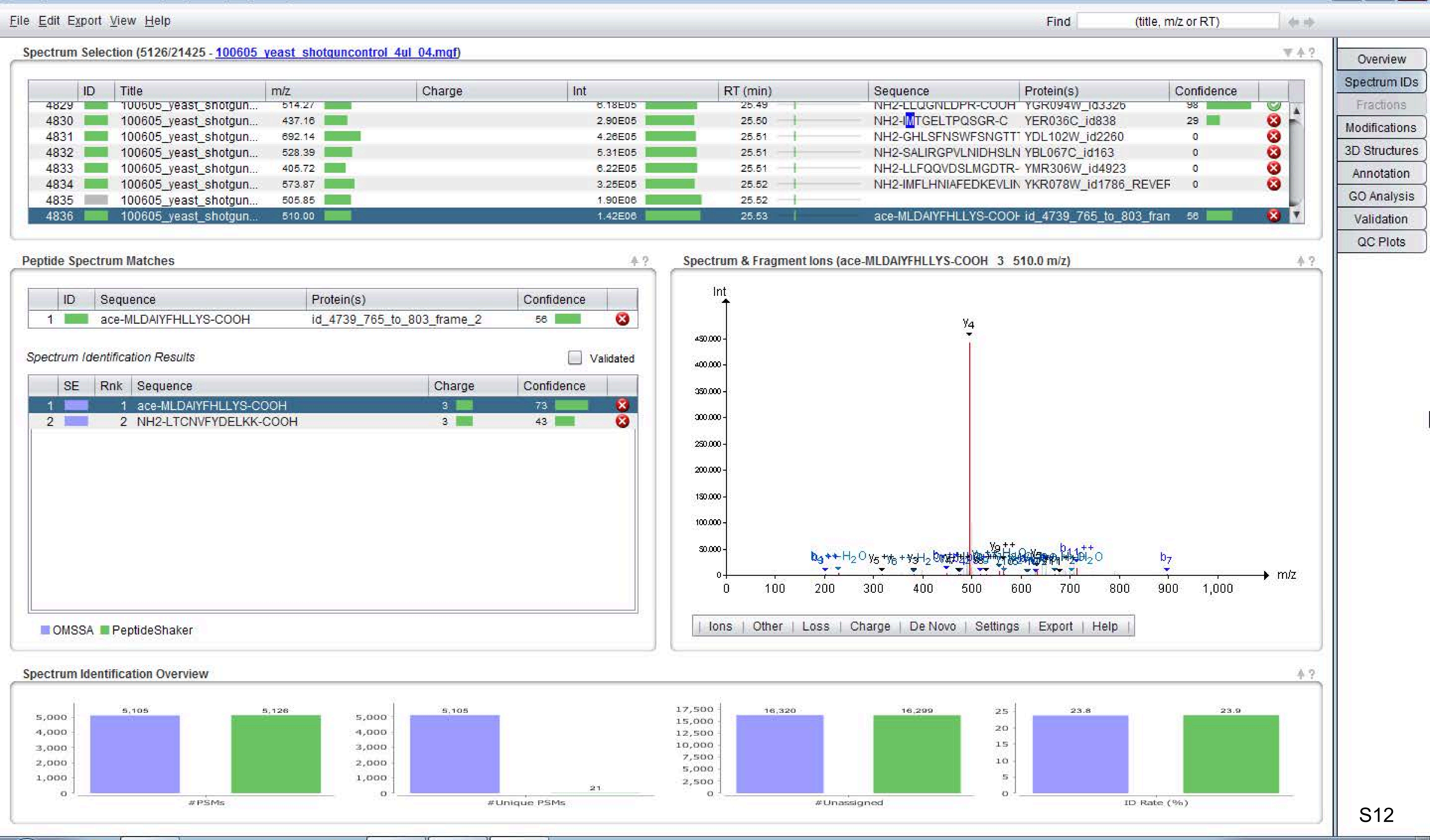
File Edit Export View Help
Find (title, m/z or RT)
Spectrum Selection (5126/21425 - 100605_yeast_shotguncontrol_4ul_04.mgf)
ID Title m/z Charge Int RT (min) Sequence Protein(s) Confidence
4829 100605_yeast_shotgun... 514.27 8.18E05 25.49 NH2-LLQGNLDPR-COOH YGR094W_id3326 98
4830 100605_yeast_shotgun... 437.16 2.90E05 25.50 NH2-IMTGELTPQSGR-C YER036C_id838 29
4831 100605_yeast_shotgun... 692.14 4.26E05 25.51 NH2-GHLSFNSWFSNGTT YDL102W_id2260 0
4832 100605_yeast_shotgun... 528.39 5.31E05 25.51 NH2-SALIRGPVLNIDHSLN YBL067C_id163 0
4833 100605_yeast_shotgun... 405.72 6.22E05 25.51 NH2-LLFQQVDSLMGDTR- YMR306W_id4923 0
4834 100605_yeast_shotgun... 573.87 3.25E05 25.52 NH2-IMFLHNIAFEDKEVLIN YKR078W_id1786_REVEF 0
4835 100605_yeast_shotgun... 505.85 1.90E06 25.52
4836 100605_yeast_shotgun... 510.00 1.42E06 25.53 ace-MLDAIYFHLLYS-COOH id_4739_765_to_803_fran 56
Overview
Spectrum IDs
Fractions
Modifications
3D Structures
Annotation
GO Analysis
Validation
QC Plots
Peptide Spectrum Matches
ID Sequence Protein(s) Confidence
1 ace-MLDAIYFHLLYS-COOH id_4739_765_to_803_frame_2 56
Spectrum Identification Results
Validated
SE Rnk Sequence Charge Confidence
1 1 ace-MLDAIYFHLLYS-COOH 3 73
2 2 NH2-LTCNVFYDELKK-COOH 3 43
OMSSA PeptideShaker
Spectrum & Fragment Ions (ace-MLDAIYFHLLYS-COOH 3 510.0 m/z)
Int
y4
450.000
400.000
350.000
300.000
250.000
200.000
150.000
100.000
50.000
0
b7
m/z
0 100 200 300 400 500 600 700 800 900 1,000
Ions Other Loss Charge De Novo Settings Export Help
Spectrum Identification Overview
5,105 5,126
#PSMs
5,105 21
#Unique PSMs
16,320 16,299
#Unassigned
23.8 23.9
ID Rate (%)

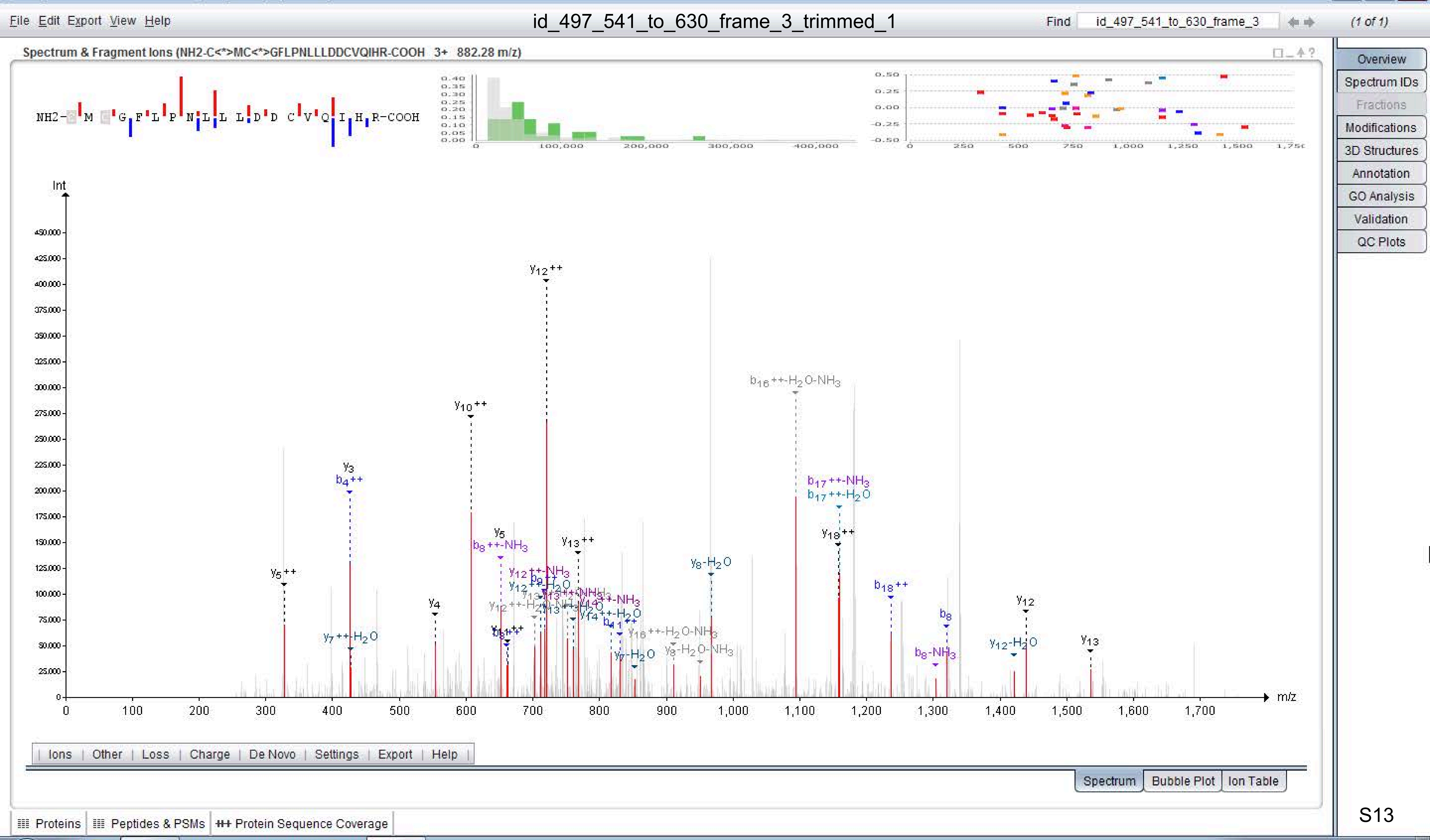

File Edit Export View Help
id_497_541_to_630_frame_3_trimmed_1
Find id_497_541_to_630_frame_3
(1 of 1)
Spectrum & Fragment Ions (NH2-C<*>MC<*>GFLPNLLLDDCVQIHR-COOH 3+ 882.28 m/z)
NH2-C M C G F L P N L L L D D C V Q I H R-COOH
Int
m/z
y12++
y10++
y3
b4++
y5++
y4
y5
b8++-NH3
y13++
y8-H2O
b16++-H2O-NH3
b17++-NH3
b17++-H2O
y18++
b18++
b8
b8-NH3
y12
y12-H2O
y13
y7++-H2O
y7-H2O
Ions Other Loss Charge De Novo Settings Export Help
Spectrum Bubble Plot Ion Table
Proteins Peptides & PSMs Protein Sequence Coverage
Overview
Spectrum IDs
Fractions
Modifications
3D Structures
Annotation
GO Analysis
Validation
QC Plots

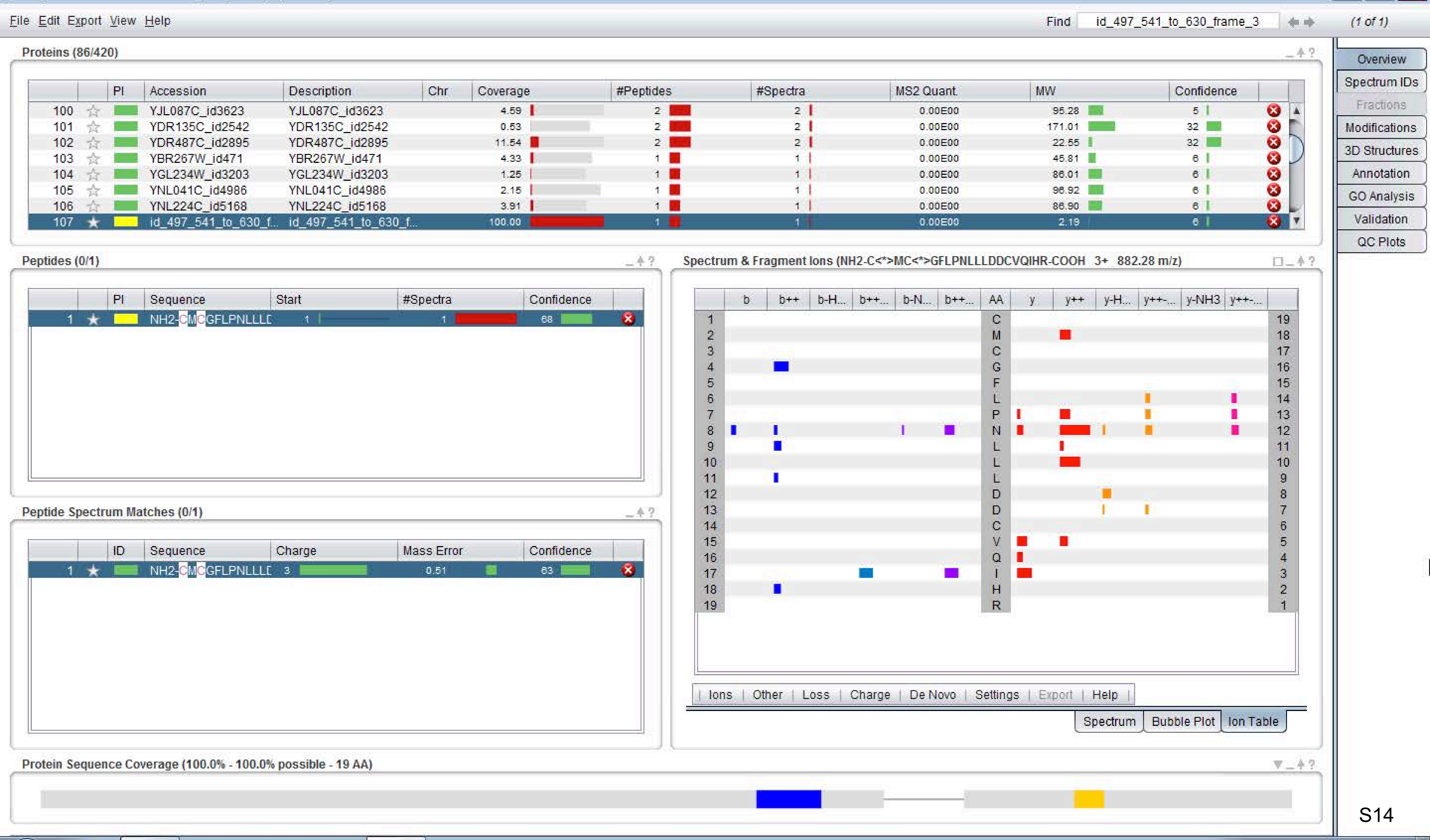
File Edit Export View Help
Find id_497_541_to_630_frame_3
(1 of 1)
Proteins (86/420)
PI Accession Description Chr Coverage #Peptides #Spectra MS2 Quant. MW Confidence
100 YJL087C_id3623 YJL087C_id3623 4.59 2 2 0.00E00 95.28 5
101 YDR135C_id2542 YDR135C_id2542 0.53 2 2 0.00E00 171.01 32
102 YDR487C_id2895 YDR487C_id2895 11.54 2 2 0.00E00 22.55 32
103 YBR267W_id471 YBR267W_id471 4.33 1 1 0.00E00 45.81 6
104 YGL234W_id3203 YGL234W_id3203 1.25 1 1 0.00E00 86.01 6
105 YNL041C_id4986 YNL041C_id4986 2.15 1 1 0.00E00 96.92 6
106 YNL224C_id5168 YNL224C_id5168 3.91 1 1 0.00E00 86.90 6
107 id_497_541_to_630_f... id_497_541_to_630_f... 100.00 1 1 0.00E00 2.19 6
Overview
Spectrum IDs
Fractions
Modifications
3D Structures
Annotation
GO Analysis
Validation
QC Plots
Peptides (0/1)
PI Sequence Start #Spectra Confidence
1 NH2-CMCGFLPNLLLD 1 1 68
Spectrum & Fragment Ions (NH2-C<*>MC<*>GFLPNLLLDDCVQIHR-COOH 3+ 882.28 m/z)
b b++ b-H... b++... b-N... b++... AA y y++ y-H... y++-... y-NH3 y++-...
C M C G F L P N L L L D D C V Q I H R
Ions Other Loss Charge De Novo Settings Export Help
Spectrum Bubble Plot Ion Table
Peptide Spectrum Matches (0/1)
ID Sequence Charge Mass Error Confidence
1 NH2-CMCGFLPNLLLD 3 0.51 63
Protein Sequence Coverage (100.0% - 100.0% possible - 19 AA)

S14

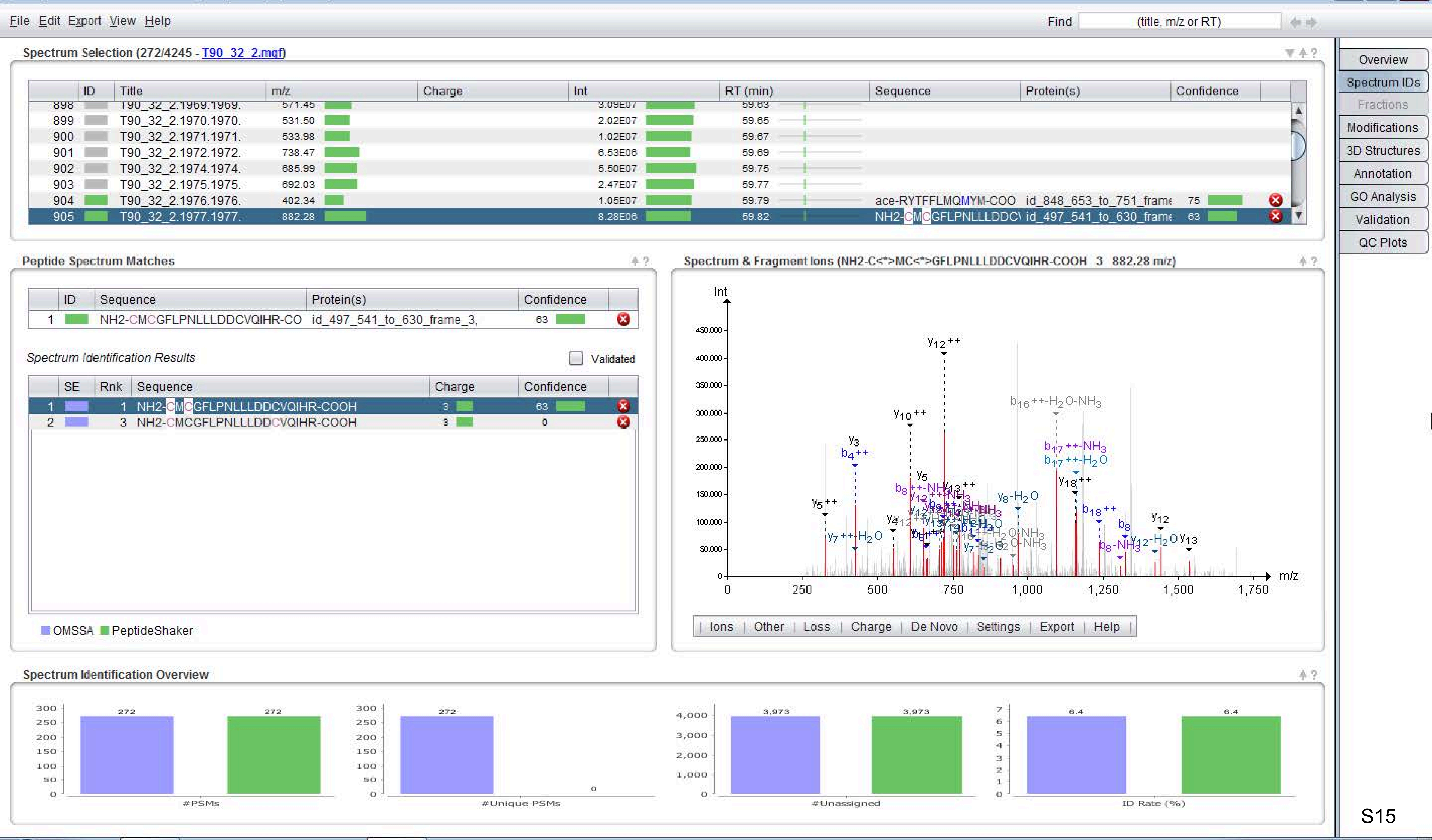
File Edit Export View Help
Find (title, m/z or RT)
Spectrum Selection (272/4245 - T90_32_2.mgf)
ID Title m/z Charge Int RT (min) Sequence Protein(s) Confidence
899 T90_32_2.1970.1970. 531.50 2.02E07 59.65
900 T90_32_2.1971.1971. 533.98 1.02E07 59.67
901 T90_32_2.1972.1972. 738.47 6.53E06 59.69
902 T90_32_2.1974.1974. 685.99 5.50E07 59.75
903 T90_32_2.1975.1975. 692.03 2.47E07 59.77
904 T90_32_2.1976.1976. 402.34 1.05E07 59.79 ace-RYTFFLMQMYM-COO id_848_653_to_751_frame 75
905 T90_32_2.1977.1977. 882.28 8.28E06 59.82 NH2-CMCGFLPNLLLDDC\ id_497_541_to_630_frame 63
Overview
Spectrum IDs
Fractions
Modifications
3D Structures
Annotation
GO Analysis
Validation
QC Plots
Peptide Spectrum Matches
ID Sequence Protein(s) Confidence
1 NH2-CMCGFLPNLLLDDCVQIHR-CO id_497_541_to_630_frame_3, 63
Spectrum Identification Results
Validated
SE Rnk Sequence Charge Confidence
1 1 NH2-CMCGFLPNLLLDDCVQIHR-COOH 3 63
2 3 NH2-CMCGFLPNLLLDDCVQIHR-COOH 3 0
OMSSA PeptideShaker
Spectrum & Fragment Ions (NH2-C<*>MC<*>GFLPNLLLDDCVQIHR-COOH 3 882.28 m/z)
Int
m/z
Ions Other Loss Charge De Novo Settings Export Help
Spectrum Identification Overview
#PSMs
#Unique PSMs
#Unassigned
ID Rate (%)

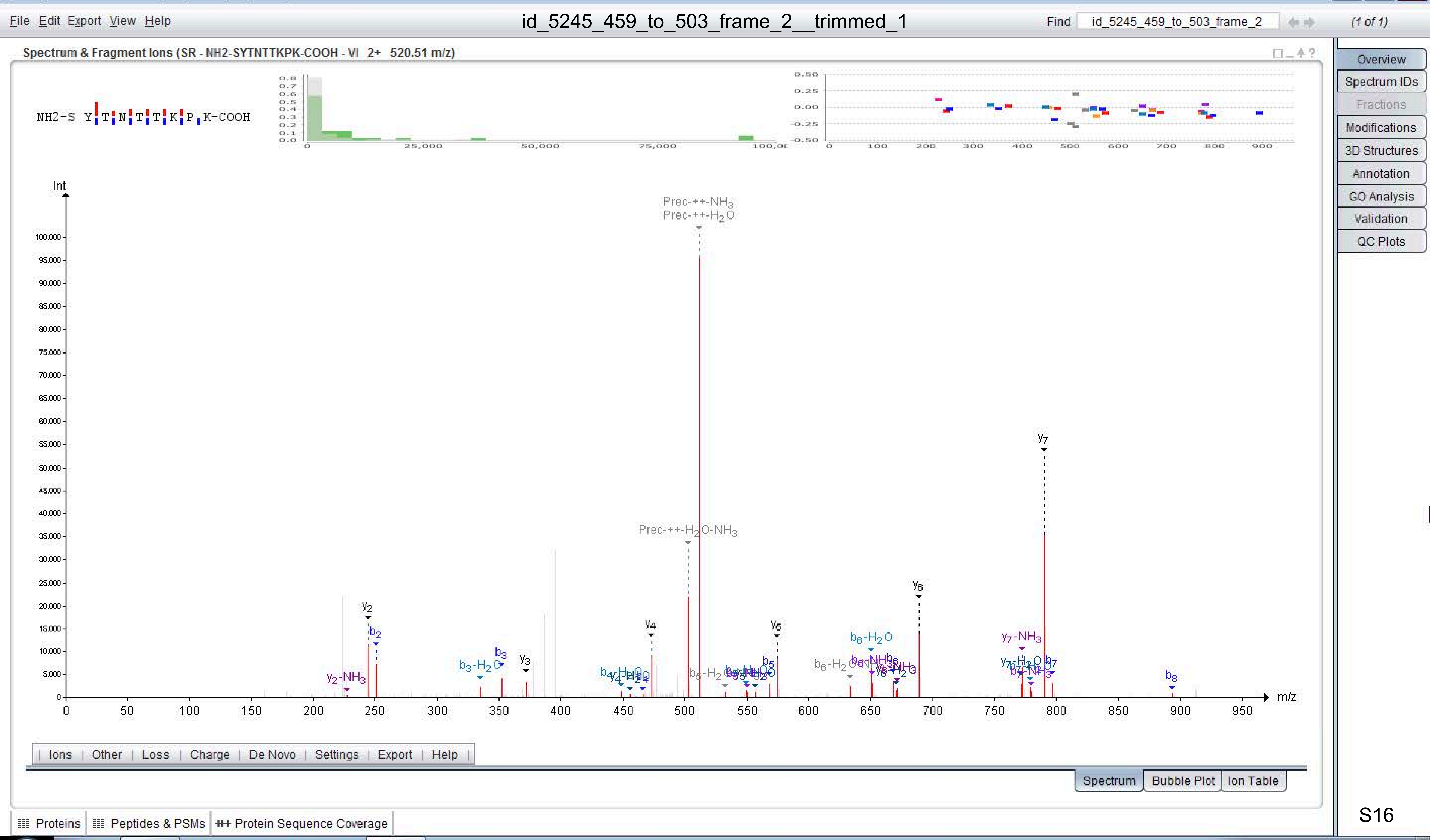
File Edit Export View Help
id_5245_459_to_503_frame_2__trimmed_1
Find id_5245_459_to_503_frame_2
(1 of 1)
Spectrum & Fragment Ions (SR - NH2-SYTNTTKPK-COOH - VI 2+ 520.51 m/z)
NH2-S Y T N T T K P K-COOH
Int
m/z
Prec-++-NH3
Prec-++-H2O
Prec-++-H2O-NH3
y7
y6
y5
y4
y3
y2
b2
b3
b3-H2O
y2-NH3
b6-H2O
y7-NH3
b8
Ions | Other | Loss | Charge | De Novo | Settings | Export | Help
Spectrum
Bubble Plot
Ion Table
Proteins
Peptides & PSMs
Protein Sequence Coverage
Overview
Spectrum IDs
Fractions
Modifications
3D Structures
Annotation
GO Analysis
Validation
QC Plots

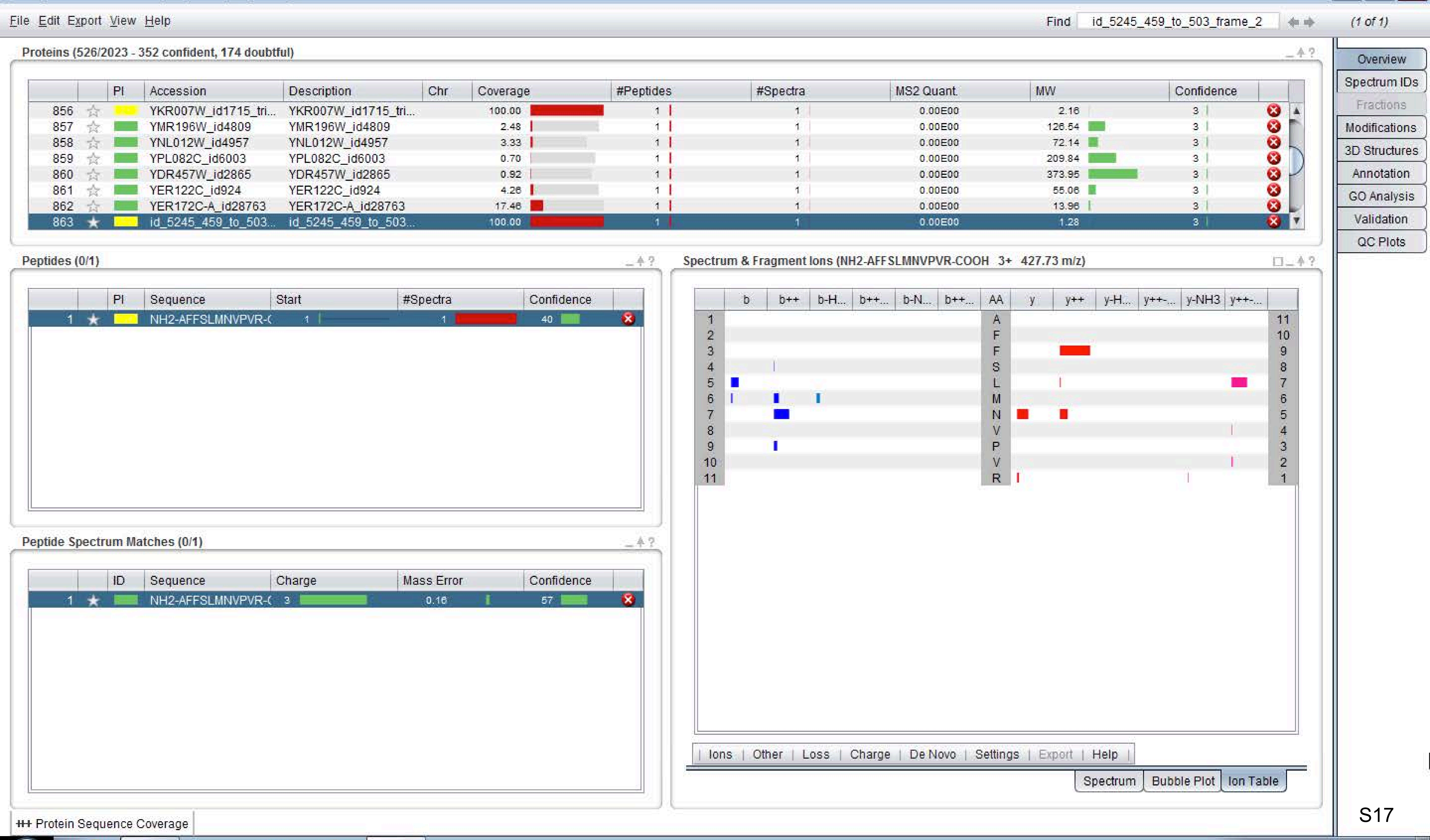
File Edit Export View Help
Find id_5245_459_to_503_frame_2
(1 of 1)
Proteins (526/2023 - 352 confident, 174 doubtful)
PI | Accession | Description | Chr | Coverage | #Peptides | #Spectra | MS2 Quant. | MW | Confidence
856 YKR007W_id1715_tri... YKR007W_id1715_tri... 100.00 1 1 0.00E00 2.16 3
857 YMR196W_id4809 YMR196W_id4809 2.48 1 1 0.00E00 126.54 3
858 YNL012W_id4957 YNL012W_id4957 3.33 1 1 0.00E00 72.14 3
859 YPL082C_id6003 YPL082C_id6003 0.70 1 1 0.00E00 209.84 3
860 YDR457W_id2865 YDR457W_id2865 0.92 1 1 0.00E00 373.95 3
861 YER122C_id924 YER122C_id924 4.26 1 1 0.00E00 55.06 3
862 YER172C-A_id28763 YER172C-A_id28763 17.46 1 1 0.00E00 13.96 3
863 id_5245_459_to_503... id_5245_459_to_503... 100.00 1 1 0.00E00 1.28 3
Overview
Spectrum IDs
Fractions
Modifications
3D Structures
Annotation
GO Analysis
Validation
QC Plots
Peptides (0/1)
PI | Sequence | Start | #Spectra | Confidence
1 NH2-AFFSLMNVPVR-(1 1 40
Spectrum & Fragment Ions (NH2-AFFSLMNVPVR-COOH 3+ 427.73 m/z)
b b++ b-H... b++... b-N... b++... AA y y++ y-H... y++-... y-NH3 y++-...
1 A 11
2 F 10
3 F 9
4 S 8
5 L 7
6 M 6
7 N 5
8 V 4
9 P 3
10 V 2
11 R 1
Peptide Spectrum Matches (0/1)
ID | Sequence | Charge | Mass Error | Confidence
1 NH2-AFFSLMNVPVR-(3 0.16 57
Ions | Other | Loss | Charge | De Novo | Settings | Export | Help
Spectrum
Bubble Plot
Ion Table
Protein Sequence Coverage

S17

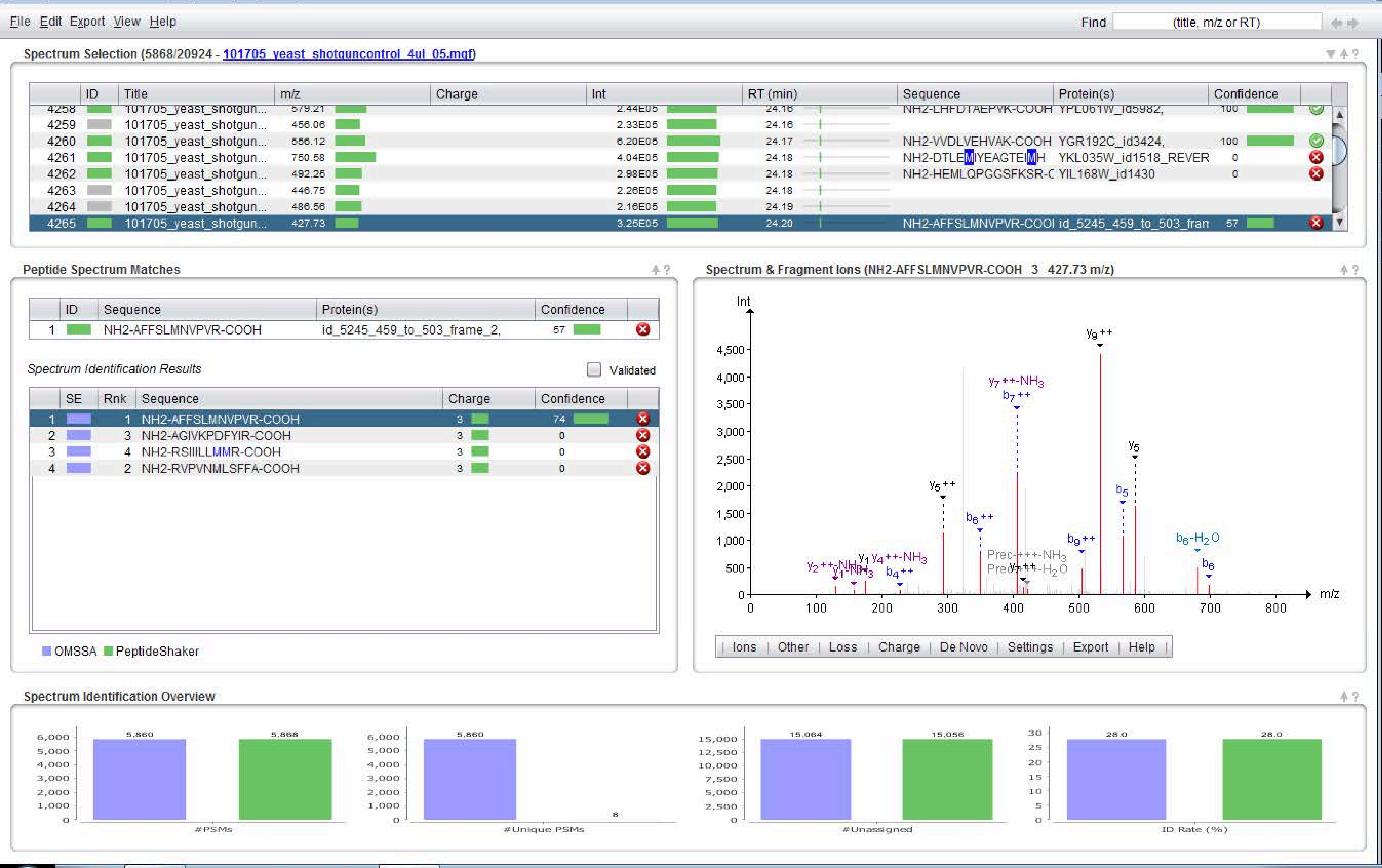
File Edit Export View Help
Find (title, m/z or RT)
Spectrum Selection (5868/20924 - 101705_yeast_shotguncontrol_4ul_05.mgf)
ID Title m/z Charge Int RT (min) Sequence Protein(s) Confidence
4259 101705_yeast_shotgun... 456.06 2.33E05 24.16
4260 101705_yeast_shotgun... 556.12 6.20E05 24.17 NH2-VVDLVEHVAK-COOH YGR192C_id3424, 100
4261 101705_yeast_shotgun... 750.58 4.04E05 24.18 NH2-DTLEMIYEAGTEIMH YKL035W_id1518_REVER 0
4262 101705_yeast_shotgun... 492.25 2.98E05 24.18 NH2-HEMLQPGGSFKSR-C YIL168W_id1430 0
4263 101705_yeast_shotgun... 446.75 2.26E05 24.18
4264 101705_yeast_shotgun... 486.56 2.16E05 24.19
4265 101705_yeast_shotgun... 427.73 3.25E05 24.20 NH2-AFFSLMNVPVR-COOI id_5245_459_to_503_fran 57
Overview
Spectrum IDs
Fractions
Modifications
3D Structures
Annotation
GO Analysis
Validation
QC Plots
Peptide Spectrum Matches
ID Sequence Protein(s) Confidence
1 NH2-AFFSLMNVPVR-COOH id_5245_459_to_503_frame_2, 57
Spectrum Identification Results
Validated
SE Rnk Sequence Charge Confidence
1 1 NH2-AFFSLMNVPVR-COOH 3 74
2 3 NH2-AGIVKPDFYIR-COOH 3 0
3 4 NH2-RSIIILLMMR-COOH 3 0
4 2 NH2-RVPVNMLSFFA-COOH 3 0
OMSSA PeptideShaker
Spectrum & Fragment Ions (NH2-AFFSLMNVPVR-COOH 3 427.73 m/z)
Int
m/z
Ions Other Loss Charge De Novo Settings Export Help
Spectrum Identification Overview
#PSMs
#Unique PSMs
#Unassigned
ID Rate (%)

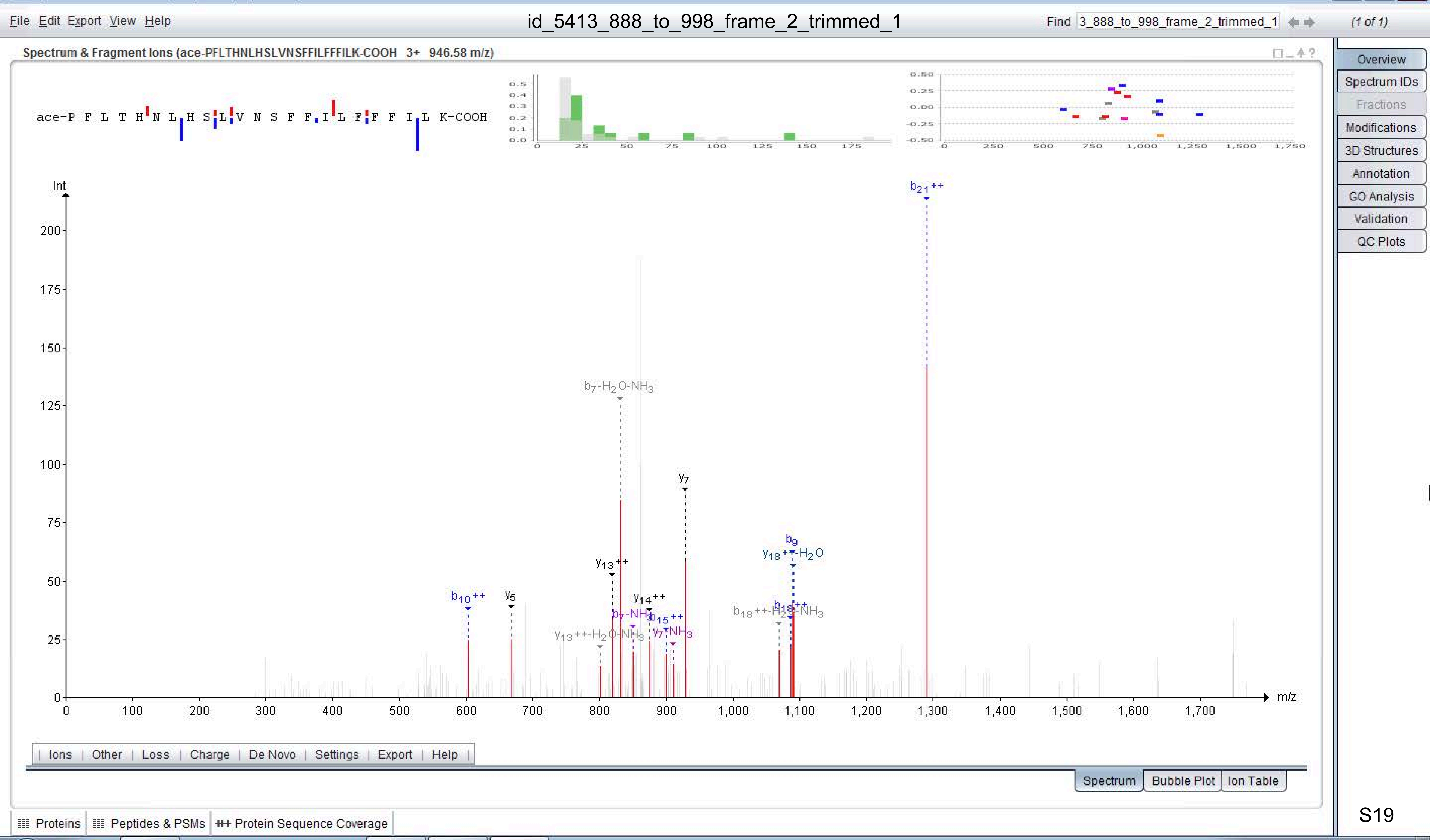

id_5413_888_to_998_frame_2_trimmed_1
File Edit Export View Help
Find 3_888_to_998_frame_2_trimmed_1
(1 of 1)
Spectrum & Fragment Ions (ace-PFLTHNLHSLVNSFFILFFFILK-COOH 3+ 946.58 m/z)
ace-P F L T H N L H S L V N S F F I L F F F I L K-COOH
Int
m/z
b21++
b7-H2O-NH3
y7
b9
y18++-H2O
y13++
b10++
y5
y14++
b7-NH3
b15++
y7-NH3
y13++-H2O-NH3
b18++-H2O-NH3
b18++
Ions | Other | Loss | Charge | De Novo | Settings | Export | Help
Spectrum
Bubble Plot
Ion Table
Overview
Spectrum IDs
Fractions
Modifications
3D Structures
Annotation
GO Analysis
Validation
QC Plots
Proteins
Peptides & PSMs
Protein Sequence Coverage

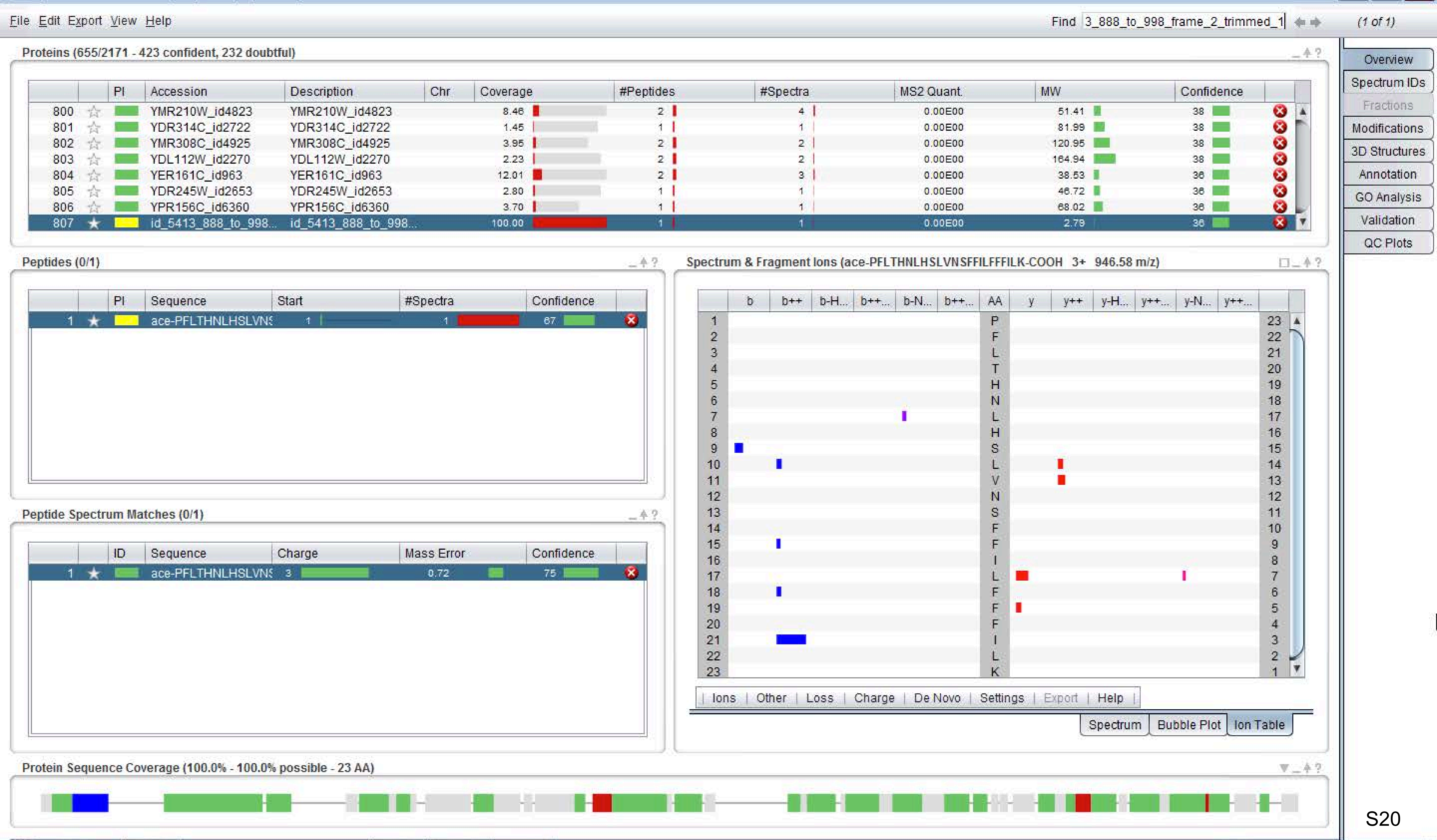
File Edit Export View Help
Find 3_888_to_998_frame_2_trimmed_1
(1 of 1)
Proteins (655/2171 - 423 confident, 232 doubtful)
PI Accession Description Chr Coverage #Peptides #Spectra MS2 Quant. MW Confidence
800 YMR210W_id4823 YMR210W_id4823 8.46 2 4 0.00E00 51.41 38
801 YDR314C_id2722 YDR314C_id2722 1.45 1 1 0.00E00 81.99 38
802 YMR308C_id4925 YMR308C_id4925 3.95 2 2 0.00E00 120.95 38
803 YDL112W_id2270 YDL112W_id2270 2.23 2 2 0.00E00 164.94 38
804 YER161C_id963 YER161C_id963 12.01 2 3 0.00E00 38.53 36
805 YDR245W_id2653 YDR245W_id2653 2.80 1 1 0.00E00 46.72 36
806 YPR156C_id6360 YPR156C_id6360 3.70 1 1 0.00E00 68.02 36
807 id_5413_888_to_998... id_5413_888_to_998... 100.00 1 1 0.00E00 2.79 36
Overview
Spectrum IDs
Fractions
Modifications
3D Structures
Annotation
GO Analysis
Validation
QC Plots
Peptides (0/1)
PI Sequence Start #Spectra Confidence
1 ace-PFLTHNLHSLVNS 1 1 67
Spectrum & Fragment Ions (ace-PFLTHNLHSLVNSFFILFFFILK-COOH 3+ 946.58 m/z)
b b++ b-H... b++... b-N... b++... AA y y++ y-H... y++... y-N... y++...
1 P 23
2 F 22
3 L 21
4 T 20
5 H 19
6 N 18
7 L 17
8 H 16
9 S 15
10 L 14
11 V 13
12 N 12
13 S 11
14 F 10
15 F 9
16 I 8
17 L 7
18 F 6
19 F 5
20 F 4
21 I 3
22 L 2
23 K 1
Ions Other Loss Charge De Novo Settings Export Help
Spectrum Bubble Plot Ion Table
Peptide Spectrum Matches (0/1)
ID Sequence Charge Mass Error Confidence
1 ace-PFLTHNLHSLVNS 3 0.72 75
Protein Sequence Coverage (100.0% - 100.0% possible - 23 AA)

S20

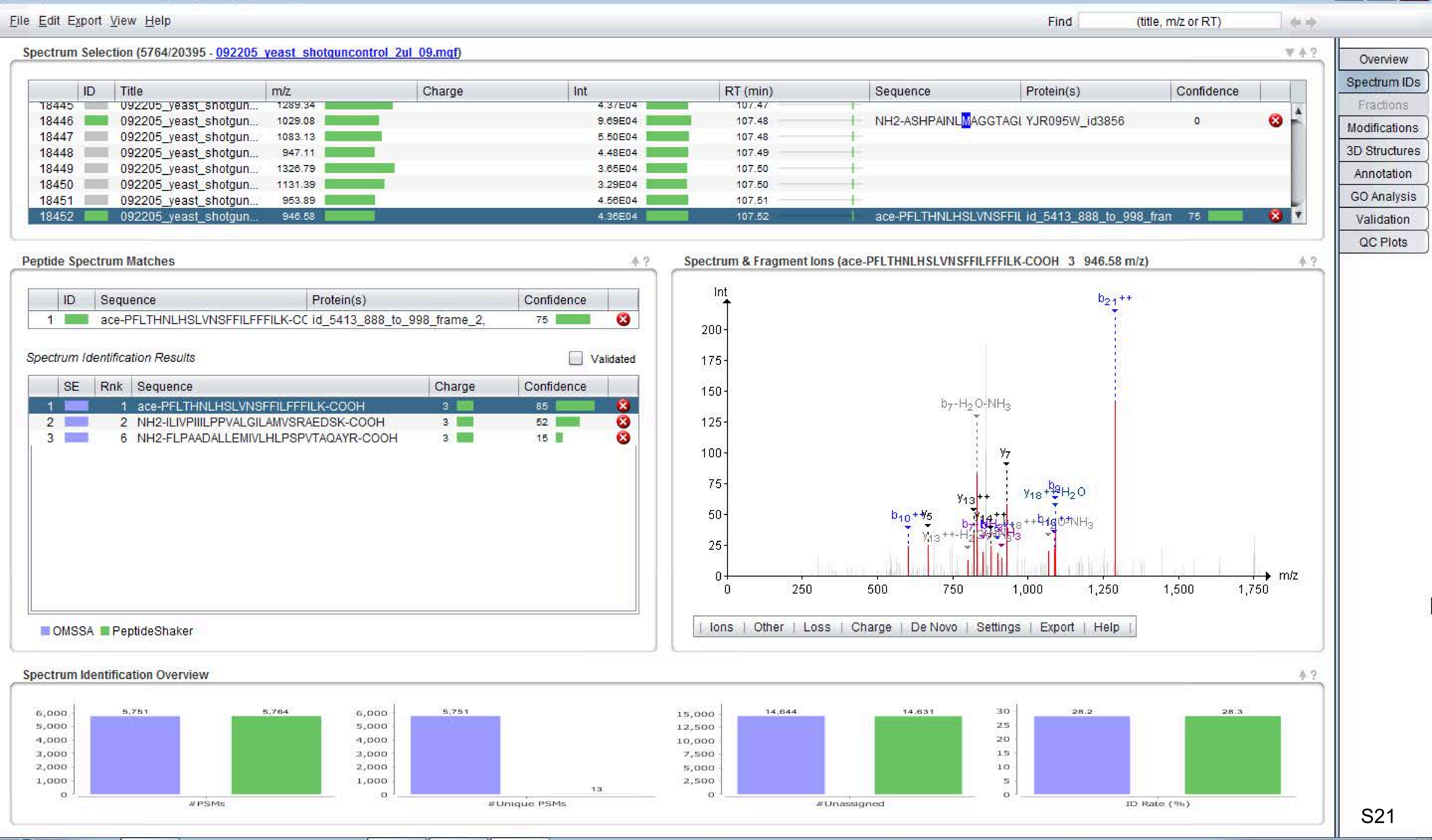
File Edit Export View Help
Find (title, m/z or RT)
Spectrum Selection (5764/20395 - 092205_yeast_shotguncontrol_2ul_09.mgf)
ID Title m/z Charge Int RT (min) Sequence Protein(s) Confidence
18446 092205_yeast_shotgun... 1029.08 9.69E04 107.48 NH2-ASHPAINLMAGGTAGI YJR095W_id3856 0
18447 092205_yeast_shotgun... 1083.13 5.50E04 107.48
18448 092205_yeast_shotgun... 947.11 4.48E04 107.49
18449 092205_yeast_shotgun... 1326.79 3.65E04 107.50
18450 092205_yeast_shotgun... 1131.39 3.29E04 107.50
18451 092205_yeast_shotgun... 953.89 4.56E04 107.51
18452 092205_yeast_shotgun... 946.58 4.36E04 107.52 ace-PFLTHNLHSLVNSFFIL id_5413_888_to_998_fran 75
Overview
Spectrum IDs
Fractions
Modifications
3D Structures
Annotation
GO Analysis
Validation
QC Plots
Peptide Spectrum Matches
ID Sequence Protein(s) Confidence
1 ace-PFLTHNLHSLVNSFFILFFFILK-CC id_5413_888_to_998_frame_2, 75
Spectrum Identification Results
Validated
SE Rnk Sequence Charge Confidence
1 1 ace-PFLTHNLHSLVNSFFILFFFILK-COOH 3 85
2 2 NH2-ILIVPIIILPPVALGILAMVSRAEDSK-COOH 3 52
3 6 NH2-FLPAADALLEMIVLHLPSPVTAQAYR-COOH 3 15
OMSSA PeptideShaker
Spectrum & Fragment Ions (ace-PFLTHNLHSLVNSFFILFFFILK-COOH 3 946.58 m/z)
Int
m/z
Ions | Other | Loss | Charge | De Novo | Settings | Export | Help
Spectrum Identification Overview
5,751 5,764
#PSMs
5,751 13
#Unique PSMs
14,644 14,631
#Unassigned
28.2 28.3
ID Rate (%)

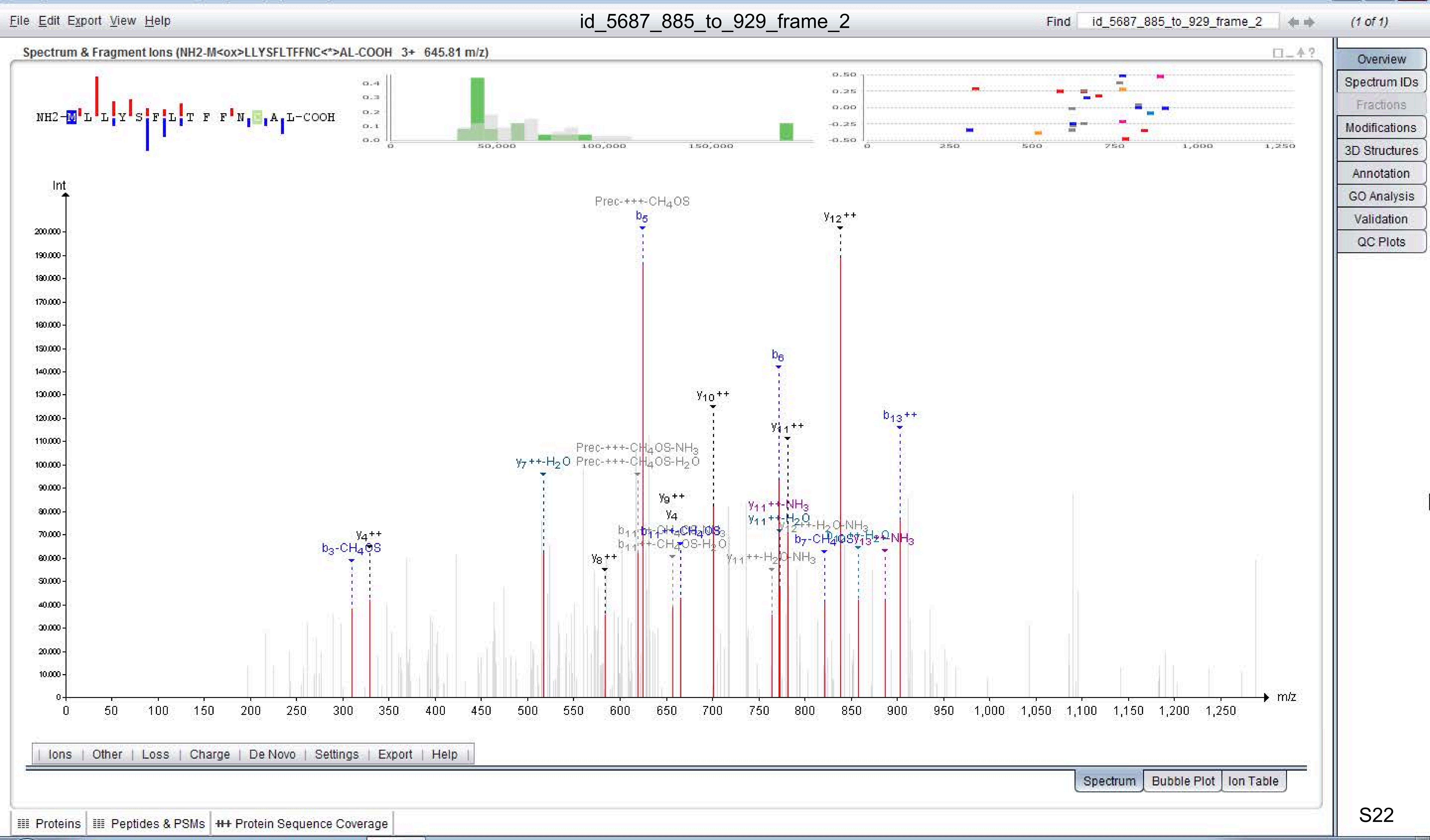
File Edit Export View Help
id_5687_885_to_929_frame_2
Find id_5687_885_to_929_frame_2
(1 of 1)
Spectrum & Fragment Ions (NH2-M<ox>LLYSFLTFFNC<*>AL-COOH 3+ 645.81 m/z)
NH2-M L L Y S F L T F F N C A L-COOH
Int
m/z
Prec-+++-CH4OS
Prec-+++-CH4OS-NH3
Prec-+++-CH4OS-H2O
b5
b6
b13++
y12++
y10++
y11++
y9++
y4
y4++
y8++
y7++-H2O
b3-CH4OS
y11++-NH3
y11++-H2O
b7-CH4OS
Ions | Other | Loss | Charge | De Novo | Settings | Export | Help
Spectrum
Bubble Plot
Ion Table
Overview
Spectrum IDs
Fractions
Modifications
3D Structures
Annotation
GO Analysis
Validation
QC Plots
Proteins
Peptides & PSMs
Protein Sequence Coverage

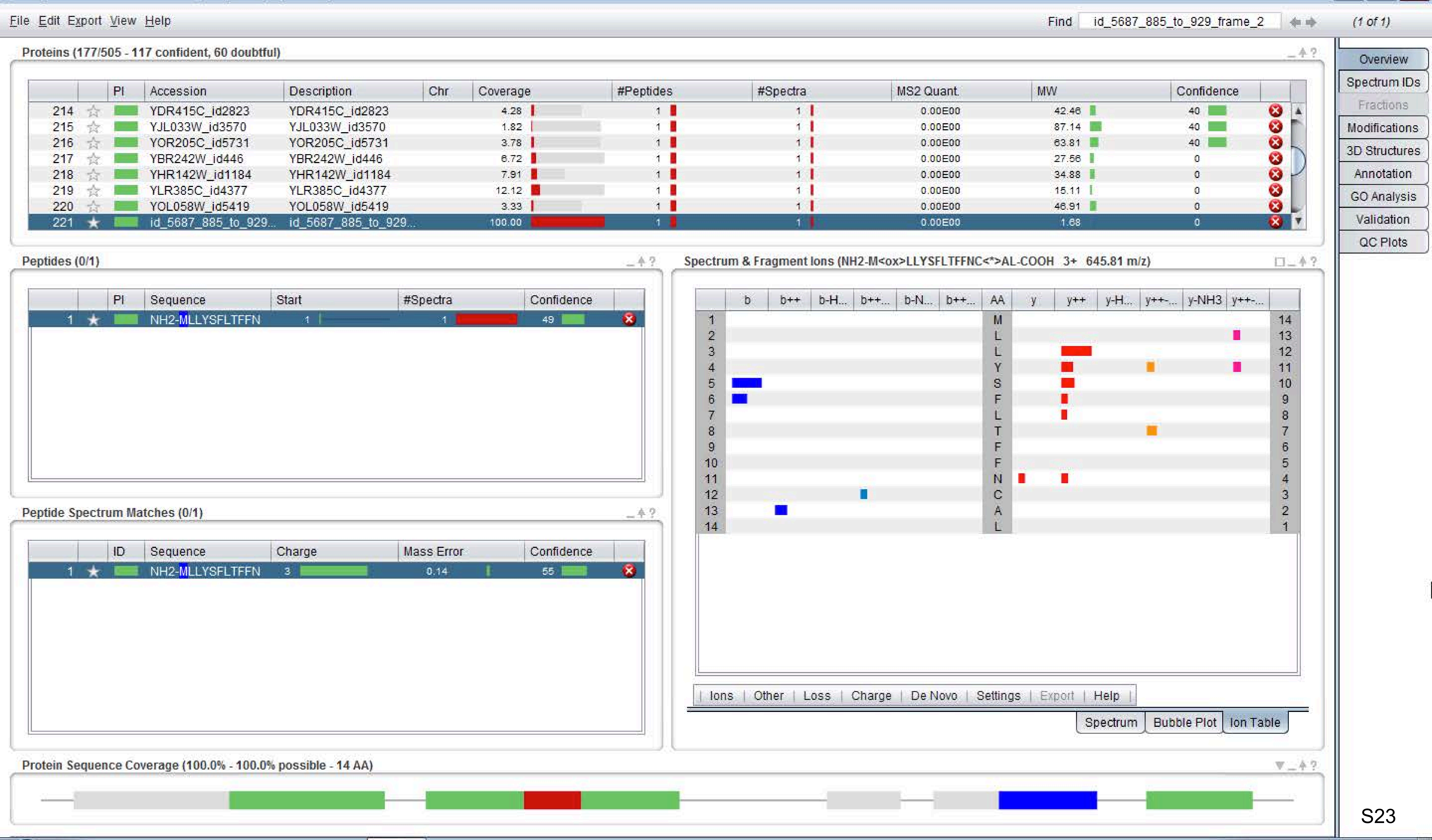
File Edit Export View Help
Find id_5687_885_to_929_frame_2
(1 of 1)
Proteins (177/505 - 117 confident, 60 doubtful)
PI Accession Description Chr Coverage #Peptides #Spectra MS2 Quant. MW Confidence
214 YDR415C_id2823 YDR415C_id2823 4.28 1 1 0.00E00 42.46 40
215 YJL033W_id3570 YJL033W_id3570 1.82 1 1 0.00E00 87.14 40
216 YOR205C_id5731 YOR205C_id5731 3.78 1 1 0.00E00 63.81 40
217 YBR242W_id446 YBR242W_id446 6.72 1 1 0.00E00 27.56 0
218 YHR142W_id1184 YHR142W_id1184 7.91 1 1 0.00E00 34.88 0
219 YLR385C_id4377 YLR385C_id4377 12.12 1 1 0.00E00 15.11 0
220 YOL058W_id5419 YOL058W_id5419 3.33 1 1 0.00E00 46.91 0
221 id_5687_885_to_929... id_5687_885_to_929... 100.00 1 1 0.00E00 1.68 0
Overview
Spectrum IDs
Fractions
Modifications
3D Structures
Annotation
GO Analysis
Validation
QC Plots
Peptides (0/1)
PI Sequence Start #Spectra Confidence
1 NH2-MLLYSFLTFFN 1 1 49
Spectrum & Fragment Ions (NH2-M<ox>LLYSFLTFFNC<*>AL-COOH 3+ 645.81 m/z)
b b++ b-H... b++... b-N... b++... AA y y++ y-H... y++-... y-NH3 y++-...
1 M 14
2 L 13
3 L 12
4 Y 11
5 S 10
6 F 9
7 L 8
8 T 7
9 F 6
10 F 5
11 N 4
12 C 3
13 A 2
14 L 1
Ions Other Loss Charge De Novo Settings Export Help
Spectrum Bubble Plot Ion Table
Peptide Spectrum Matches (0/1)
ID Sequence Charge Mass Error Confidence
1 NH2-MLLYSFLTFFN 3 0.14 55
Protein Sequence Coverage (100.0% - 100.0% possible - 14 AA)

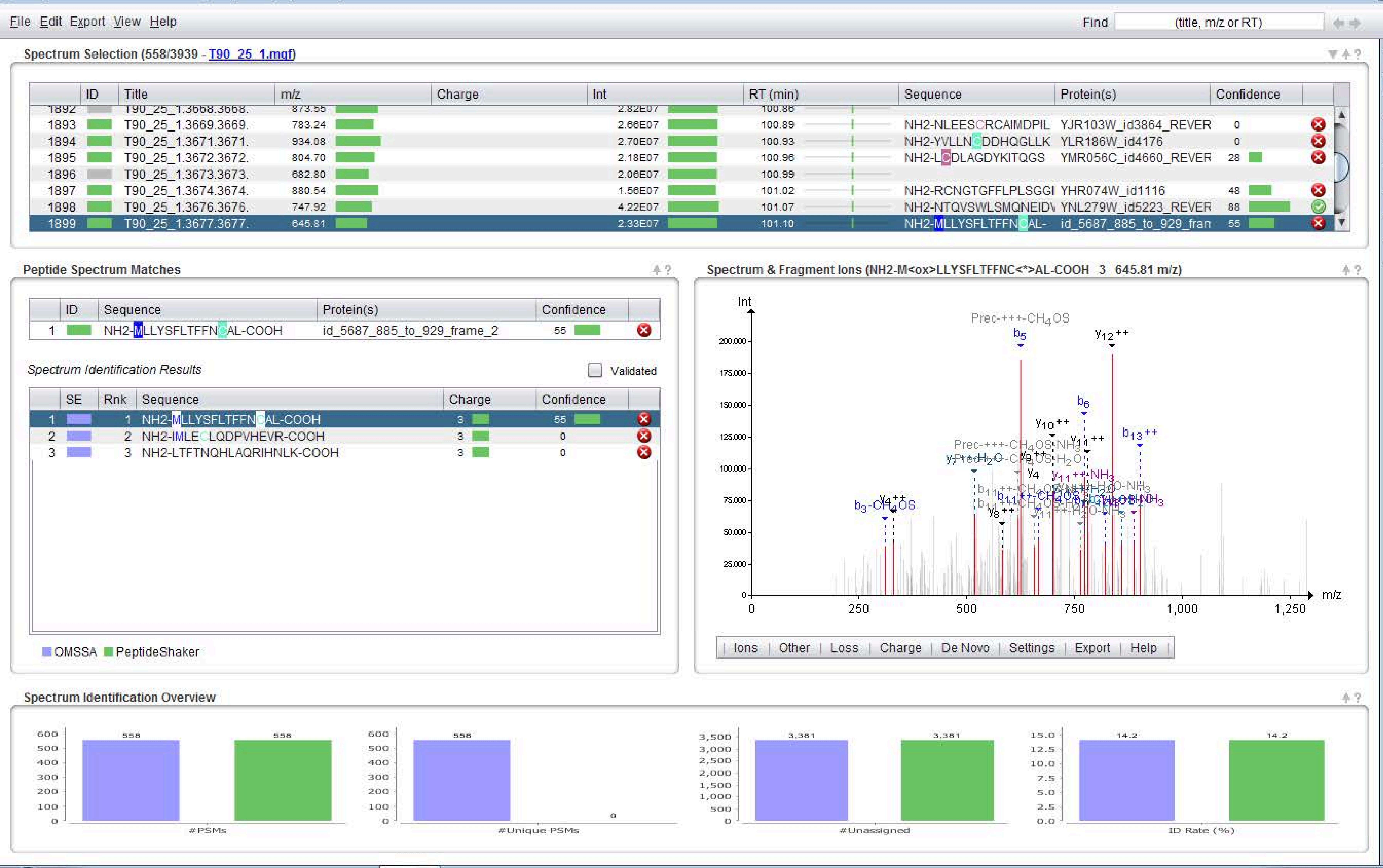
File Edit Export View Help
Find (title, m/z or RT)
Spectrum Selection (558/3939 - T90_25_1.mgf)
ID Title m/z Charge Int RT (min) Sequence Protein(s) Confidence
1893 T90_25_1.3669.3669. 783.24 2.66E07 100.89 NH2-NLEESCRCAIMDPIL YJR103W_id3864_REVER 0
1894 T90_25_1.3671.3671. 934.08 2.70E07 100.93 NH2-YVLLNCDDHQGLLK YLR186W_id4176 0
1895 T90_25_1.3672.3672. 804.70 2.18E07 100.96 NH2-LCDLAGDYKITQGS YMR056C_id4660_REVER 28
1896 T90_25_1.3673.3673. 682.80 2.06E07 100.99
1897 T90_25_1.3674.3674. 880.54 1.56E07 101.02 NH2-RCNGTGFFLPLSGGI YHR074W_id1116 48
1898 T90_25_1.3676.3676. 747.92 4.22E07 101.07 NH2-NTQVSWLSMQNEID\ YNL279W_id5223_REVER 88
1899 T90_25_1.3677.3677. 645.81 2.33E07 101.10 NH2-MLLYSFLTFFNCAL- id_5687_885_to_929_fran 55
Overview
Spectrum IDs
Fractions
Modifications
3D Structures
Annotation
GO Analysis
Validation
QC Plots
Peptide Spectrum Matches
ID Sequence Protein(s) Confidence
1 NH2-MLLYSFLTFFNCAL-COOH id_5687_885_to_929_frame_2 55
Spectrum Identification Results
Validated
SE Rnk Sequence Charge Confidence
1 1 NH2-MLLYSFLTFFNCAL-COOH 3 55
2 2 NH2-IMLECLQDPVHEVR-COOH 3 0
3 3 NH2-LTFTNQHLAQRIHNLK-COOH 3 0
OMSSA PeptideShaker
Spectrum & Fragment Ions (NH2-M<ox>LLYSFLTFFNC<*>AL-COOH 3 645.81 m/z)
Int
m/z
0 250 500 750 1,000 1,250
Ions Other Loss Charge De Novo Settings Export Help
Spectrum Identification Overview
#PSMs
#Unique PSMs
#Unassigned
ID Rate (%)
558 558 558 0 3,381 3,381 14.2 14.2

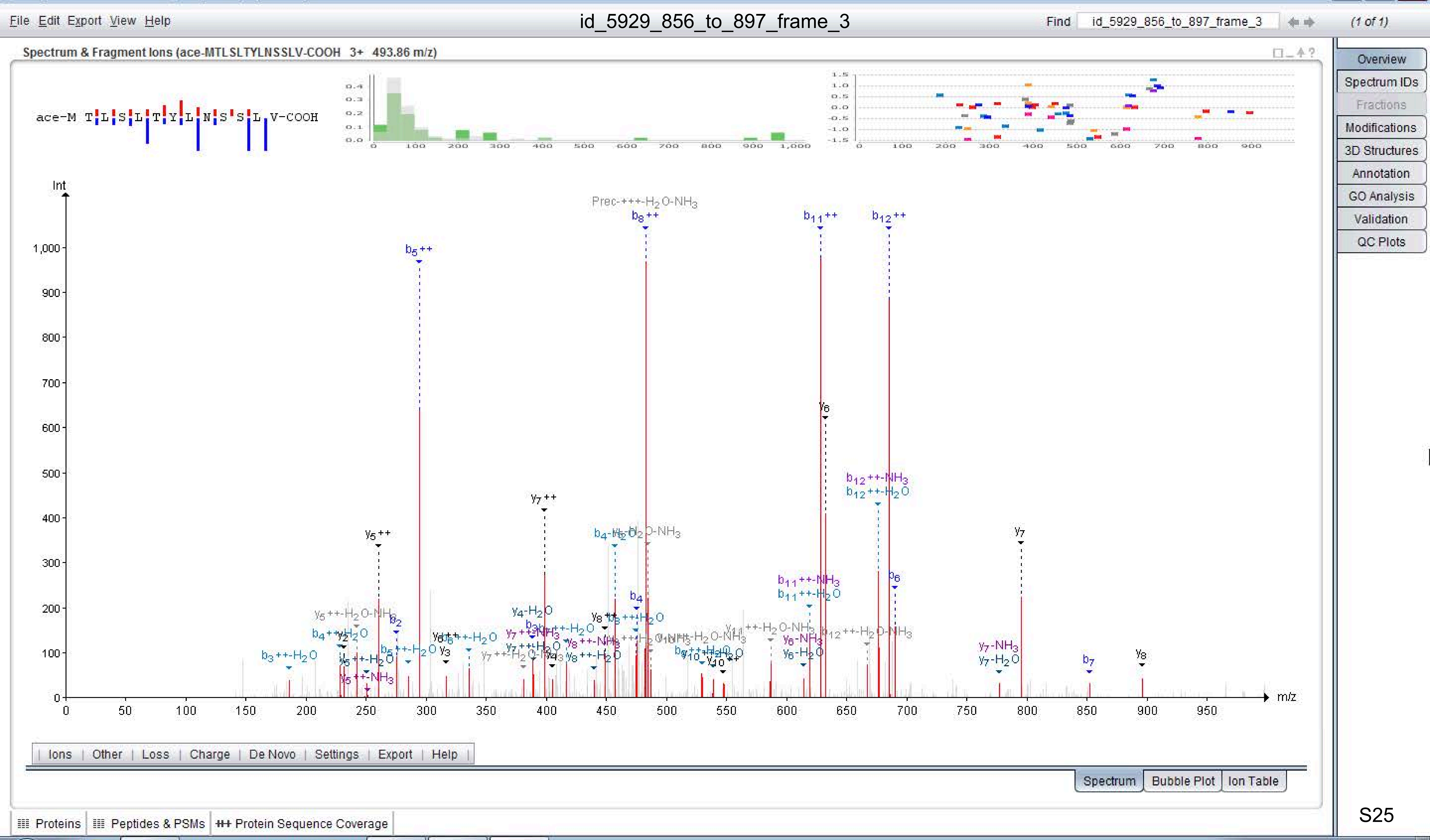

id_5929_856_to_897_frame_3
File Edit Export View Help
Find id_5929_856_to_897_frame_3 (1 of 1)
Spectrum & Fragment Ions (ace-MTLSLTYLNSSLV-COOH 3+ 493.86 m/z)
ace-M T L S L T Y L N S S L V-COOH
Overview
Spectrum IDs
Fractions
Modifications
3D Structures
Annotation
GO Analysis
Validation
QC Plots
Int
m/z
Prec-+++-H2O-NH3
b8++
b5++
b11++
b12++
y6
b12++-NH3
b12++-H2O
y7++
y5++
y7
b11++-NH3
b11++-H2O
b6
b4
y7-NH3
y7-H2O
b7
y8
Ions Other Loss Charge De Novo Settings Export Help
Spectrum Bubble Plot Ion Table
Proteins Peptides & PSMs Protein Sequence Coverage

File Edit Export View Help   Find id_5929_856_to_897_frame_3   (1 of 1)

Overview | Spectrum IDs | Fractions | Modifications | 3D Structures | Annotation | GO Analysis | Validation | QC Plots

Proteins (454/1685 - 328 confident, 126 doubtful)

		PI	Accession	Description	Chr	Coverage	#Peptides	#Spectra	MS2 Quant.	MW	Confidence
634	☆		YDR292C_id2700	YDR292C_id2700		2.74	1	1	0.00E00	69.24	26
635	☆		YER013W_id815	YER013W_id815		1.48	1	1	0.00E00	129.93	26
636	☆		YHR198C_id1241	YHR198C_id1241		6.85	1	1	0.00E00	36.48	26
637	☆		YLR046C_id4036	YLR046C_id4036		5.93	1	1	0.00E00	31.03	26
638	☆		YLR277C_id4267	YLR277C_id4267		2.95	1	1	0.00E00	87.62	26
639	☆		YMR076C_id4681	YMR076C_id4681		1.33	1	1	0.00E00	146.95	26
640	☆		YMR092C_id4698	YMR092C_id4698		3.41	1	1	0.00E00	67.28	26
641	★		id_5929_856_to_897...	id_5929_856_to_897...		100.00	1	1	0.00E00	1.44	26

Peptides (0/1)

		PI	Sequence	Start	#Spectra	Confidence
1	★		ace-MTLSLTYLNSSLV	1	1	71

Spectrum & Fragment Ions (ace-MTLSLTYLNSSLV-COOH 3+ 493.86 m/z)

	b	b++	b-H...	b++...	b-N...	b++...	AA	y	y++	y-H...	y++-...	y-NH3	y++-...	
1							M							13
2							T							12
3							L							11
4							S							10
5							L							9
6							T							8
7							Y							7
8							L							6
9							N							5
10							S							4
11							S							3
12							L							2
13							V							1

Ions | Other | Loss | Charge | De Novo | Settings | Export | Help

Spectrum | Bubble Plot | Ion Table

Peptide Spectrum Matches (0/1)

		ID	Sequence	Charge	Mass Error	Confidence
1	★		ace-MTLSLTYLNSSLV	3	1.40	79

Protein Sequence Coverage

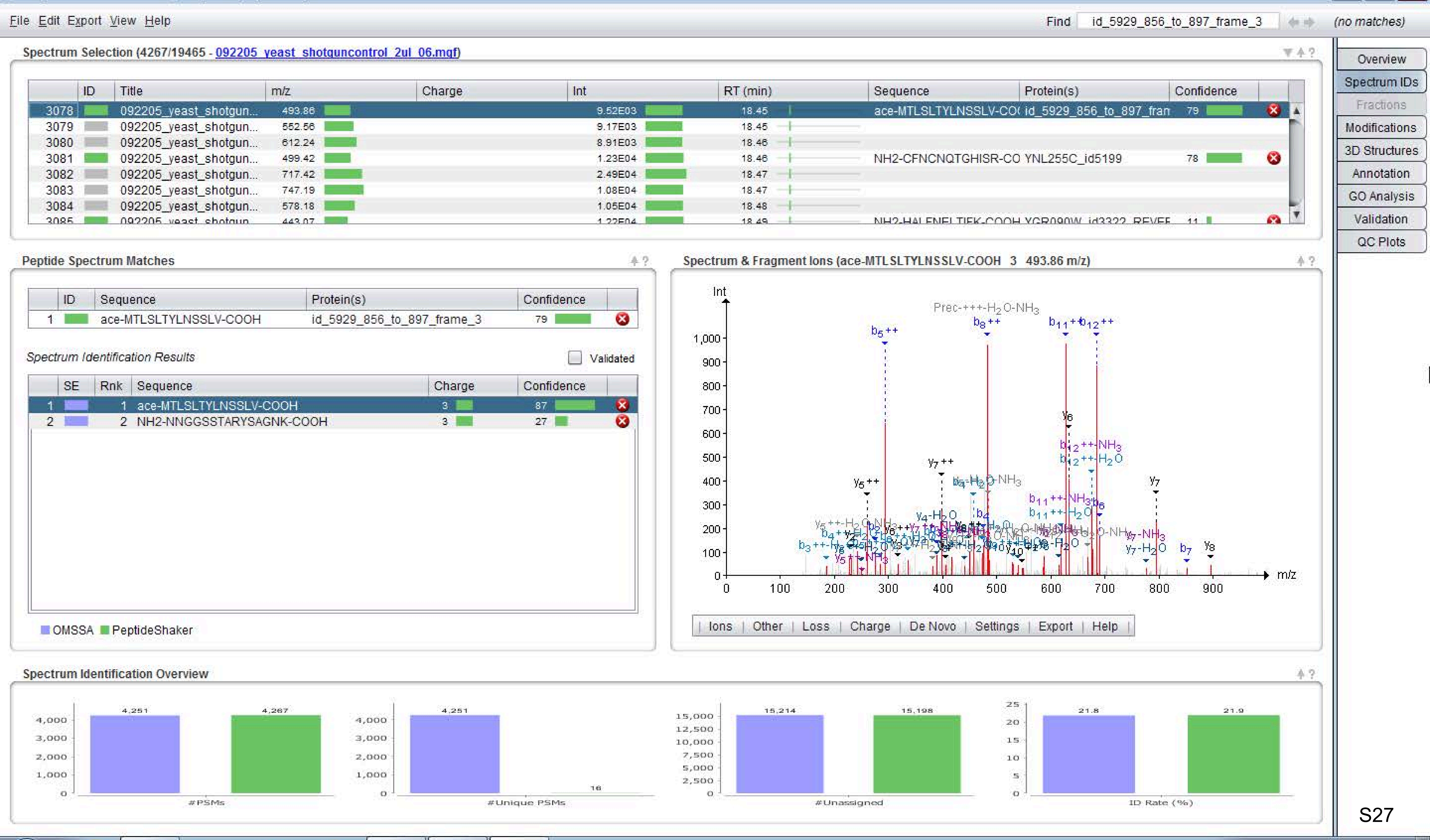
File Edit Export View Help
Find id_5929_856_to_897_frame_3
(no matches)
Spectrum Selection (4267/19465 - 092205_yeast_shotguncontrol_2ul_06.mgf)
ID Title m/z Charge Int RT (min) Sequence Protein(s) Confidence
3078 092205_yeast_shotgun... 493.86 9.52E03 18.45 ace-MTLSLTYLNSSLV-CO(id_5929_856_to_897_fram 79
3079 092205_yeast_shotgun... 552.56 9.17E03 18.45
3080 092205_yeast_shotgun... 612.24 8.91E03 18.46
3081 092205_yeast_shotgun... 499.42 1.23E04 18.46 NH2-CFNCNQTGHISR-CO YNL255C_id5199 78
3082 092205_yeast_shotgun... 717.42 2.49E04 18.47
3083 092205_yeast_shotgun... 747.19 1.08E04 18.47
3084 092205_yeast_shotgun... 578.18 1.05E04 18.48
Overview
Spectrum IDs
Fractions
Modifications
3D Structures
Annotation
GO Analysis
Validation
QC Plots
Peptide Spectrum Matches
ID Sequence Protein(s) Confidence
1 ace-MTLSLTYLNSSLV-COOH id_5929_856_to_897_frame_3 79
Spectrum Identification Results
Validated
SE Rnk Sequence Charge Confidence
1 1 ace-MTLSLTYLNSSLV-COOH 3 87
2 2 NH2-NNGGSSTARYSAGNK-COOH 3 27
OMSSA PeptideShaker
Spectrum & Fragment Ions (ace-MTLSLTYLNSSLV-COOH 3 493.86 m/z)
Int
m/z
Prec-+++-H2O-NH3
b5++
b8++
b11++
b12++
y6
y7++
y5++
y7
y8
b7
Ions | Other | Loss | Charge | De Novo | Settings | Export | Help
Spectrum Identification Overview
4,251
4,267
#PSMs
4,251
16
#Unique PSMs
15,214
15,198
#Unassigned
21.8
21.9
ID Rate (%)

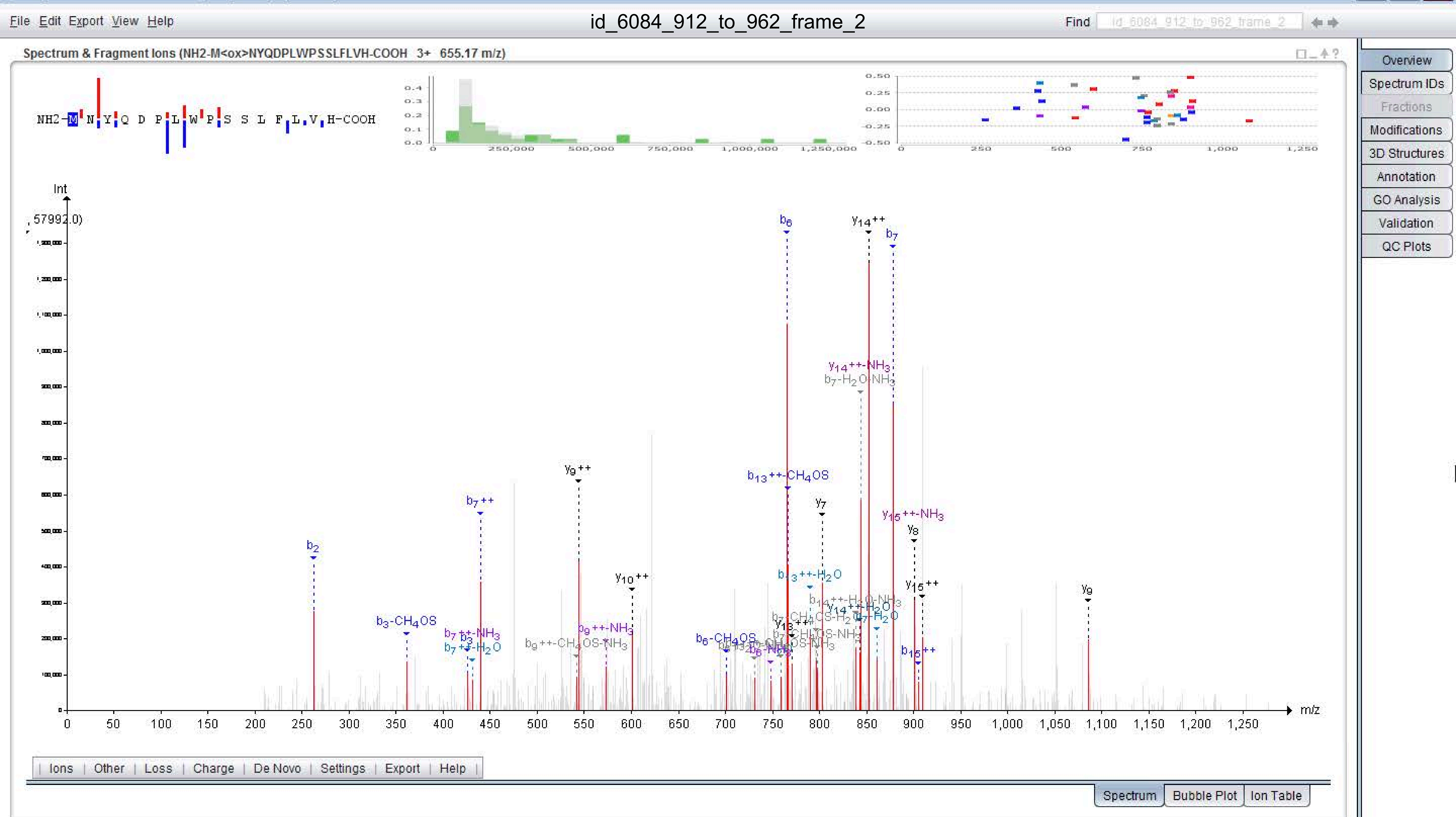
File Edit Export View Help
id_6084_912_to_962_frame_2
Find
Spectrum & Fragment Ions (NH2-M<ox>NYQDPLWPSSLFLVH-COOH 3+ 655.17 m/z)
NH2-M N Y Q D P L W P S S L F L V H-COOH
Int
m/z
b2
b3-CH4OS
b7++
y9++
y10++
b13++-CH4OS
b6
y14++
b7
y7
y8
y15++-NH3
y14++-NH3
y15++
y9
b15++
Ions | Other | Loss | Charge | De Novo | Settings | Export | Help
Spectrum
Bubble Plot
Ion Table
Proteins
Peptides & PSMs
Protein Sequence Coverage
Overview
Spectrum IDs
Fractions
Modifications
3D Structures
Annotation
GO Analysis
Validation
QC Plots

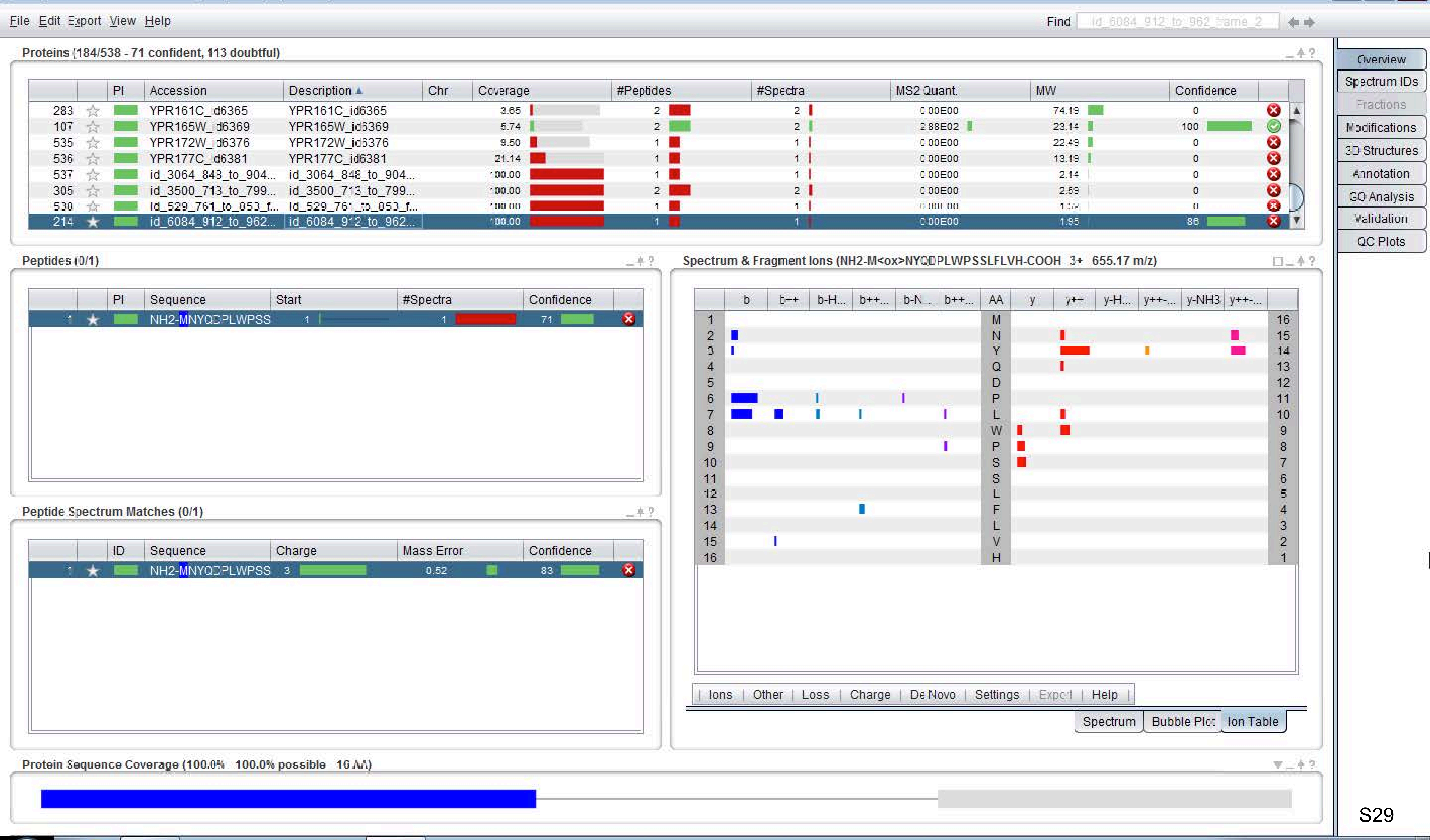

File Edit Export View Help
Find id_6084_912_to_962_frame_2
Proteins (184/538 - 71 confident, 113 doubtful)
PI Accession Description Chr Coverage #Peptides #Spectra MS2 Quant. MW Confidence
283 YPR161C_id6365 YPR161C_id6365 3.65 2 2 0.00E00 74.19 0
107 YPR165W_id6369 YPR165W_id6369 5.74 2 2 2.88E02 23.14 100
535 YPR172W_id6376 YPR172W_id6376 9.50 1 1 0.00E00 22.49 0
536 YPR177C_id6381 YPR177C_id6381 21.14 1 1 0.00E00 13.19 0
537 id_3064_848_to_904... id_3064_848_to_904... 100.00 1 1 0.00E00 2.14 0
305 id_3500_713_to_799... id_3500_713_to_799... 100.00 2 2 0.00E00 2.59 0
538 id_529_761_to_853_f... id_529_761_to_853_f... 100.00 1 1 0.00E00 1.32 0
214 id_6084_912_to_962... id_6084_912_to_962... 100.00 1 1 0.00E00 1.95 86
Overview
Spectrum IDs
Fractions
Modifications
3D Structures
Annotation
GO Analysis
Validation
QC Plots
Peptides (0/1)
PI Sequence Start #Spectra Confidence
1 NH2-MNYQDPLWPSS 1 1 71
Peptide Spectrum Matches (0/1)
ID Sequence Charge Mass Error Confidence
1 NH2-MNYQDPLWPSS 3 0.52 83
Spectrum & Fragment Ions (NH2-M<ox>NYQDPLWPSSLFLVH-COOH 3+ 655.17 m/z)
b b++ b-H... b++... b-N... b++... AA y y++ y-H... y++-... y-NH3 y++-...
1 M 16
2 N 15
3 Y 14
4 Q 13
5 D 12
6 P 11
7 L 10
8 W 9
9 P 8
10 S 7
11 S 6
12 L 5
13 F 4
14 L 3
15 V 2
16 H 1
Ions Other Loss Charge De Novo Settings Export Help
Spectrum Bubble Plot Ion Table
Protein Sequence Coverage (100.0% - 100.0% possible - 16 AA)

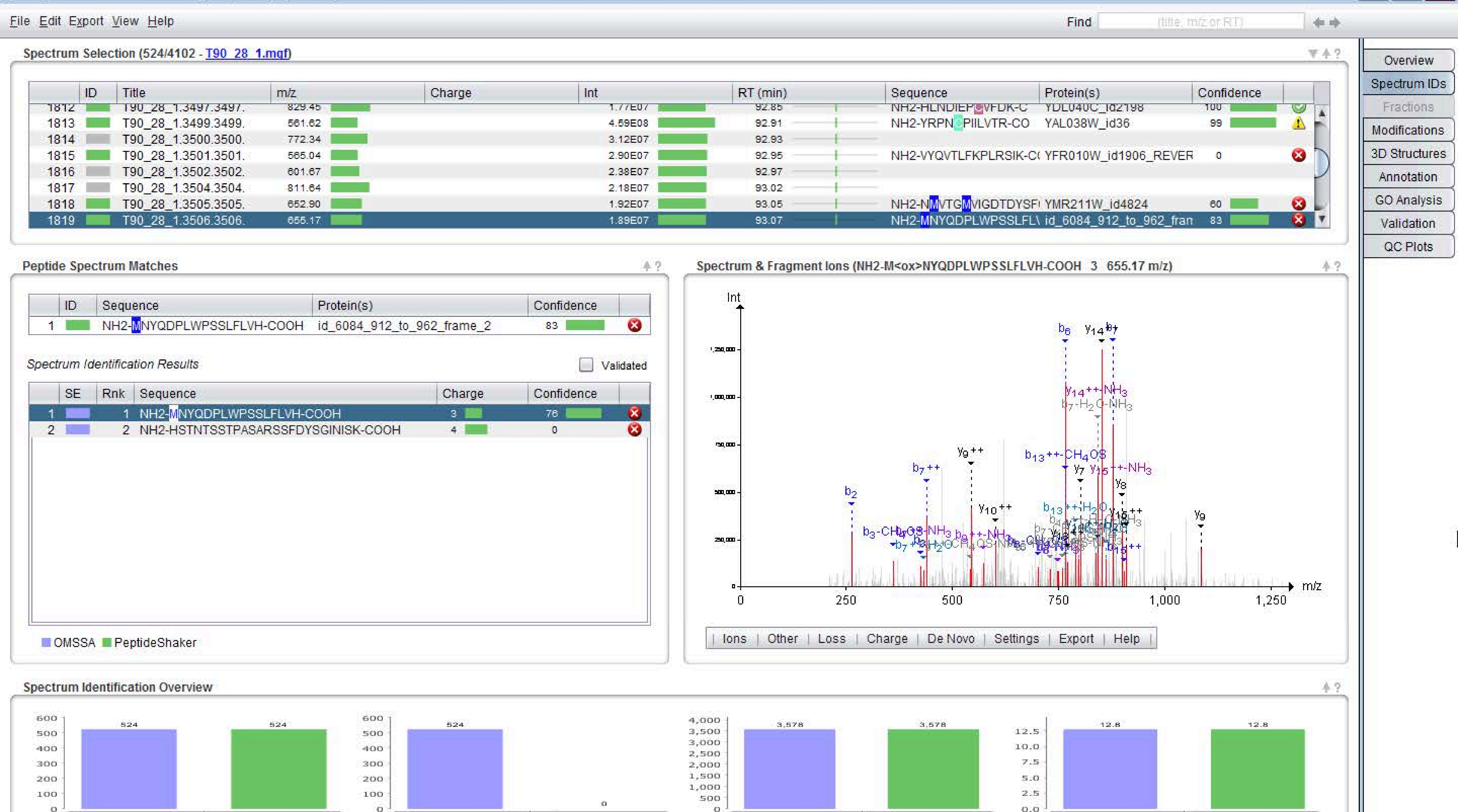
File Edit Export View Help
Find
Spectrum Selection (524/4102 - T90_28_1.mgf)
ID Title m/z Charge Int RT (min) Sequence Protein(s) Confidence
1813 T90_28_1.3499.3499. 561.62 4.59E08 92.91 NH2-YRPNCPIILVTR-CO YAL038W_id36 99
1814 T90_28_1.3500.3500. 772.34 3.12E07 92.93
1815 T90_28_1.3501.3501. 565.04 2.90E07 92.95 NH2-VYQVTLFKPLRSIK-C YFR010W_id1906_REVER 0
1816 T90_28_1.3502.3502. 601.67 2.38E07 92.97
1817 T90_28_1.3504.3504. 811.64 2.18E07 93.02
1818 T90_28_1.3505.3505. 652.90 1.92E07 93.05 NH2-NMVTGMVIGDTDYSF YMR211W_id4824 60
1819 T90_28_1.3506.3506. 655.17 1.89E07 93.07 NH2-MNYQDPLWPSSLFL id_6084_912_to_962_fran 83
Overview
Spectrum IDs
Fractions
Modifications
3D Structures
Annotation
GO Analysis
Validation
QC Plots
Peptide Spectrum Matches
ID Sequence Protein(s) Confidence
1 NH2-MNYQDPLWPSSLFLVH-COOH id_6084_912_to_962_frame_2 83
Spectrum Identification Results
Validated
SE Rnk Sequence Charge Confidence
1 1 NH2-MNYQDPLWPSSLFLVH-COOH 3 76
2 2 NH2-HSTNTSSTPASARSSFDYSGINISK-COOH 4 0
OMSSA PeptideShaker
Spectrum & Fragment Ions (NH2-M<ox>NYQDPLWPSSLFLVH-COOH 3 655.17 m/z)
Int
m/z
0 250 500 750 1,000 1,250
Ions Other Loss Charge De Novo Settings Export Help
Spectrum Identification Overview
#PSMs
#Unique PSMs
#Unassigned
ID Rate (%)

S30

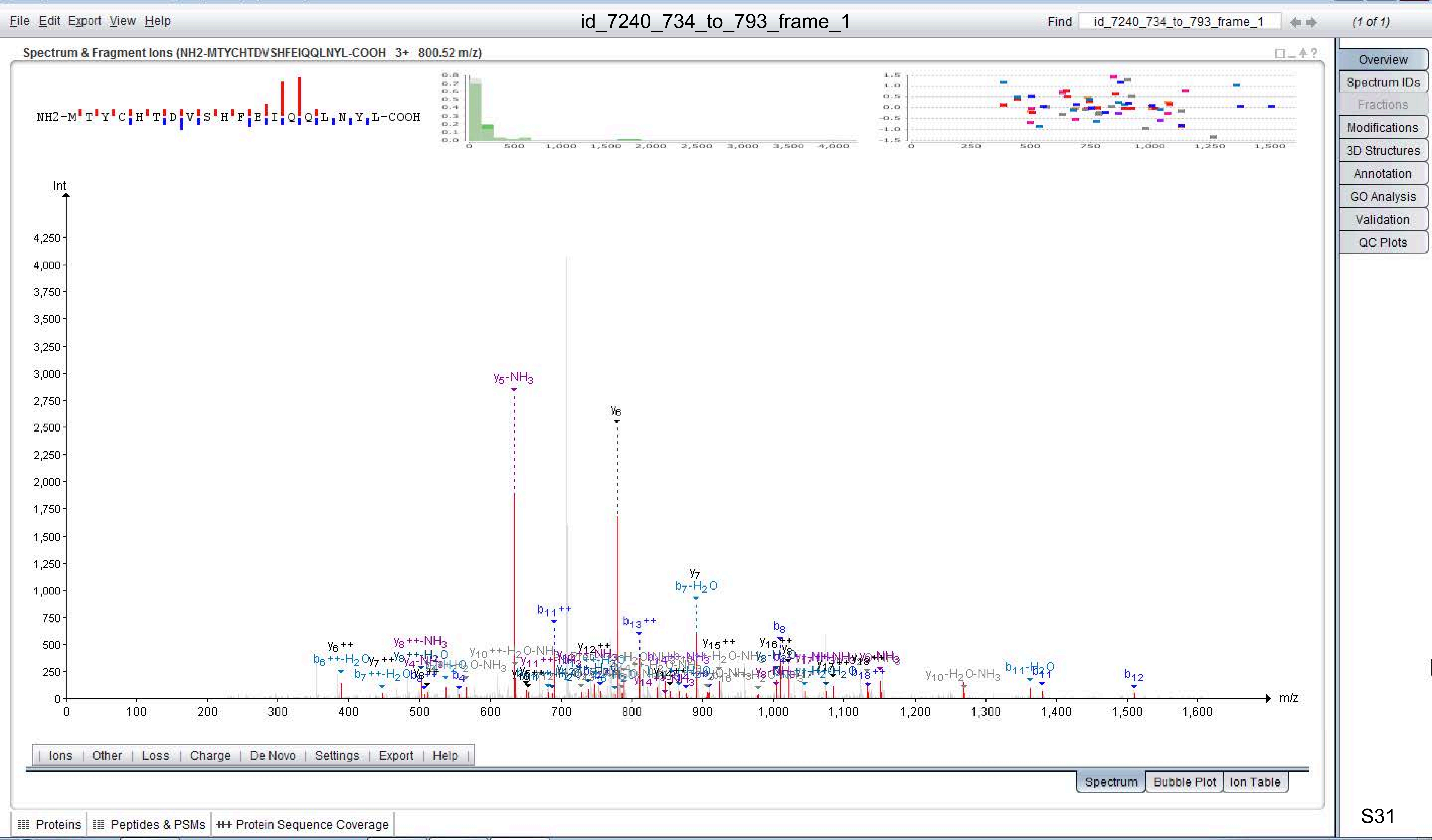
File Edit Export View Help
id_7240_734_to_793_frame_1
Find id_7240_734_to_793_frame_1
(1 of 1)
Spectrum & Fragment Ions (NH2-MTYCHTDVSHFEIQQLNYL-COOH 3+ 800.52 m/z)
NH2-M T Y C H T D V S H F E I Q Q L N Y L-COOH
Int
m/z
y5-NH3
y6
y7
b7-H2O
b11++
b13++
b8
y15++
y16++
y12++
y6++
y8++-NH3
y10-H2O-NH3
b11-H2O
b11
b12
Ions Other Loss Charge De Novo Settings Export Help
Spectrum Bubble Plot Ion Table
Overview
Spectrum IDs
Fractions
Modifications
3D Structures
Annotation
GO Analysis
Validation
QC Plots
Proteins
Peptides & PSMs
Protein Sequence Coverage

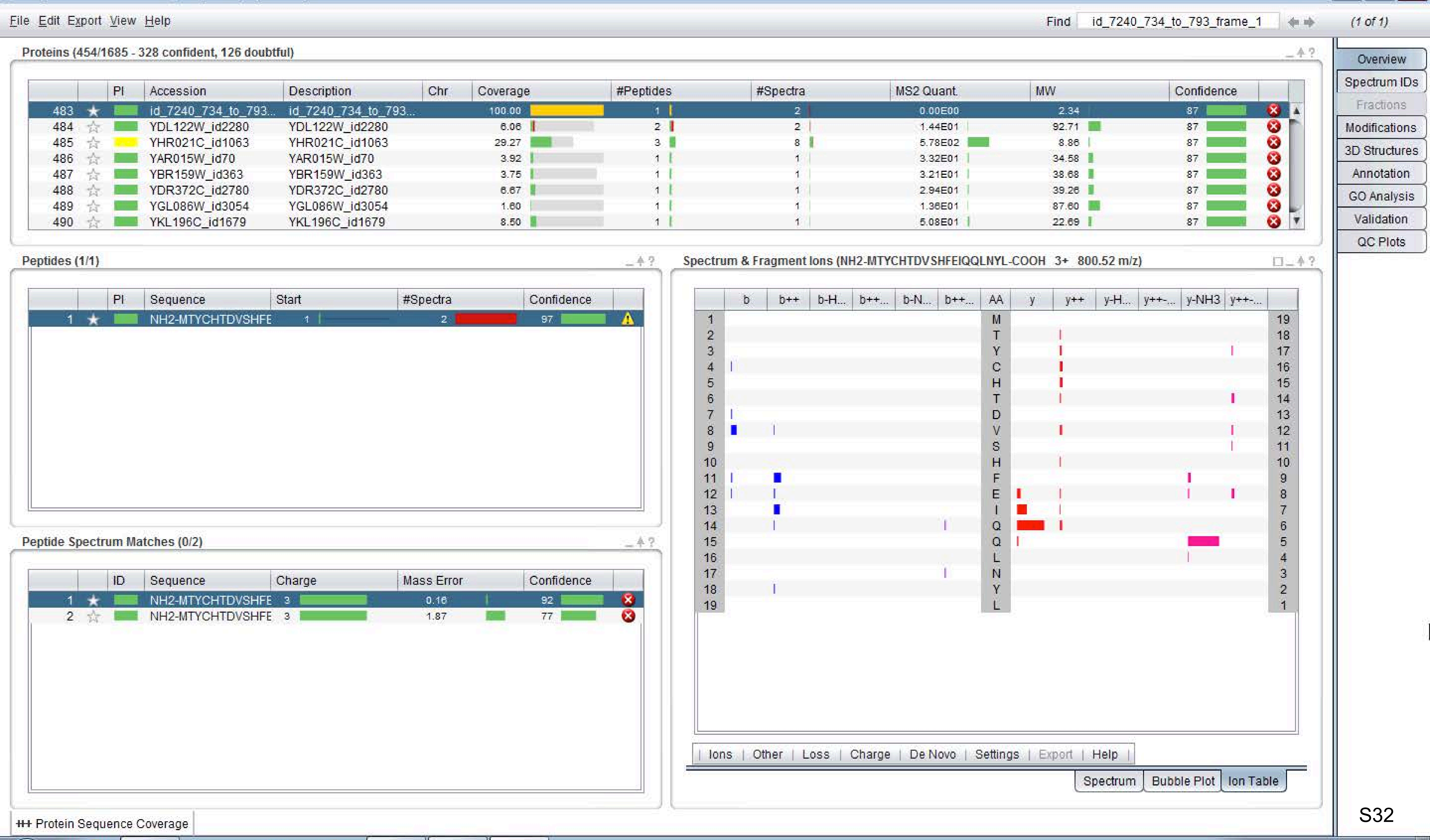
File Edit Export View Help
Find id_7240_734_to_793_frame_1 (1 of 1)
Proteins (454/1685 - 328 confident, 126 doubtful)
PI Accession Description Chr Coverage #Peptides #Spectra MS2 Quant. MW Confidence
483 id_7240_734_to_793... id_7240_734_to_793... 100.00 1 2 0.00E00 2.34 87
484 YDL122W_id2280 YDL122W_id2280 6.06 2 2 1.44E01 92.71 87
485 YHR021C_id1063 YHR021C_id1063 29.27 3 8 5.78E02 8.86 87
486 YAR015W_id70 YAR015W_id70 3.92 1 1 3.32E01 34.58 87
487 YBR159W_id363 YBR159W_id363 3.75 1 1 3.21E01 38.68 87
488 YDR372C_id2780 YDR372C_id2780 6.67 1 1 2.94E01 39.26 87
489 YGL086W_id3054 YGL086W_id3054 1.60 1 1 1.36E01 87.60 87
490 YKL196C_id1679 YKL196C_id1679 8.50 1 1 5.08E01 22.69 87
Overview
Spectrum IDs
Fractions
Modifications
3D Structures
Annotation
GO Analysis
Validation
QC Plots
Peptides (1/1)
PI Sequence Start #Spectra Confidence
1 NH2-MTYCHTDVSHFE 1 2 97
Spectrum & Fragment Ions (NH2-MTYCHTDVSHFEIQQLNYL-COOH 3+ 800.52 m/z)
b b++ b-H... b++... b-N... b++... AA y y++ y-H... y++-... y-NH3 y++-...
1 M 19
2 T 18
3 Y 17
4 C 16
5 H 15
6 T 14
7 D 13
8 V 12
9 S 11
10 H 10
11 F 9
12 E 8
13 I 7
14 Q 6
15 Q 5
16 L 4
17 N 3
18 Y 2
19 L 1
Peptide Spectrum Matches (0/2)
ID Sequence Charge Mass Error Confidence
1 NH2-MTYCHTDVSHFE 3 0.16 92
2 NH2-MTYCHTDVSHFE 3 1.87 77
Ions Other Loss Charge De Novo Settings Export Help
Spectrum Bubble Plot Ion Table
Protein Sequence Coverage

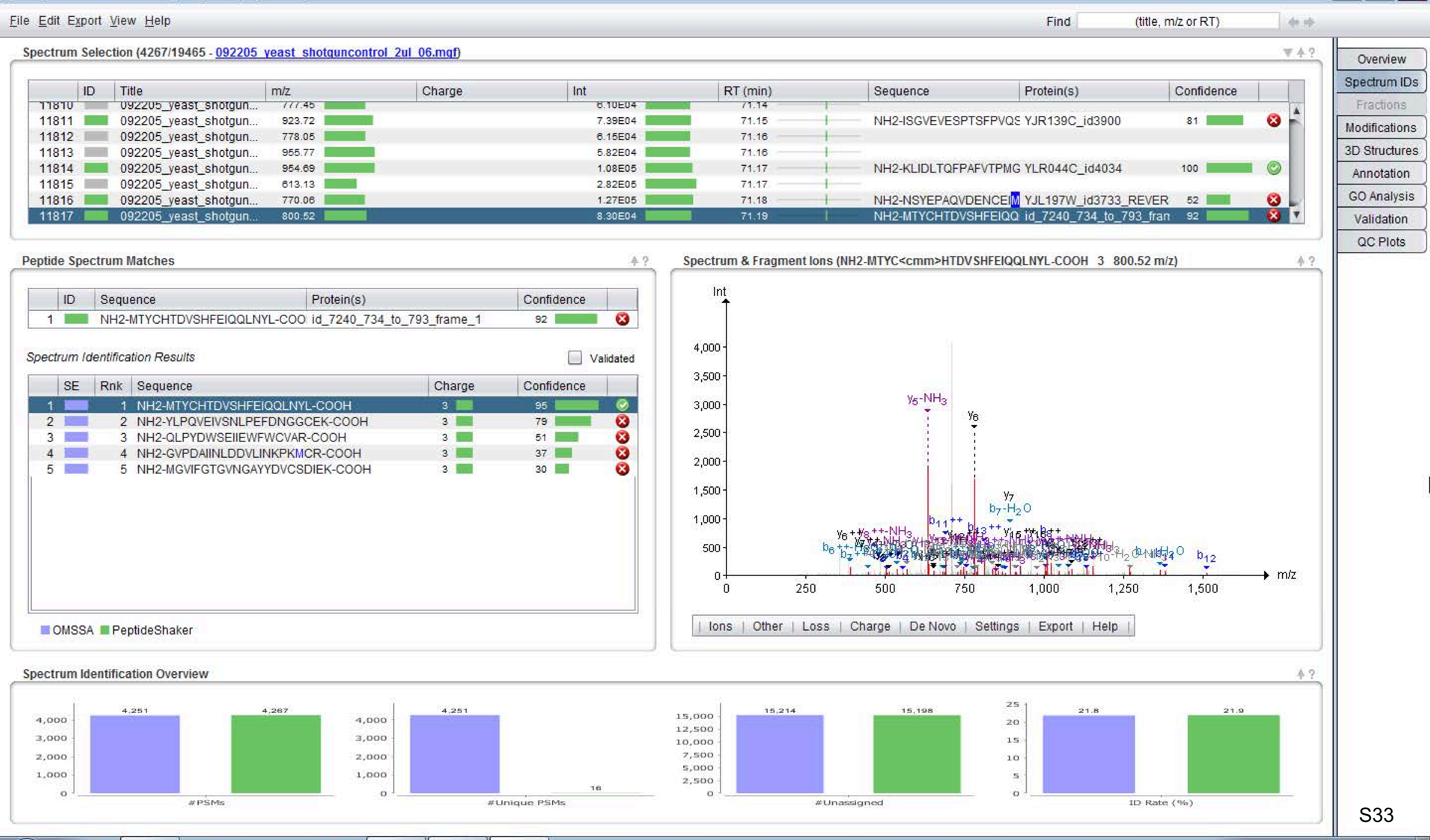
File Edit Export View Help
Find (title, m/z or RT)
Spectrum Selection (4267/19465 - 092205_yeast_shotguncontrol_2ul_06.mgf)
ID | Title | m/z | Charge | Int | RT (min) | Sequence | Protein(s) | Confidence
11811 092205_yeast_shotgun... 923.72 7.39E04 71.15 NH2-ISGVEVESPTSFPVQS YJR139C_id3900 81
11812 092205_yeast_shotgun... 778.05 6.15E04 71.16
11813 092205_yeast_shotgun... 955.77 5.82E04 71.16
11814 092205_yeast_shotgun... 954.69 1.08E05 71.17 NH2-KLIDLTQFPAFVTPMG YLR044C_id4034 100
11815 092205_yeast_shotgun... 613.13 2.82E05 71.17
11816 092205_yeast_shotgun... 770.06 1.27E05 71.18 NH2-NSYEPAQVDENCEIM YJL197W_id3733_REVER 52
11817 092205_yeast_shotgun... 800.52 8.30E04 71.19 NH2-MTYCHTDVSHFEIQQ id_7240_734_to_793_fran 92
Overview
Spectrum IDs
Fractions
Modifications
3D Structures
Annotation
GO Analysis
Validation
QC Plots
Peptide Spectrum Matches
ID | Sequence | Protein(s) | Confidence
1 NH2-MTYCHTDVSHFEIQQLNYL-COO id_7240_734_to_793_frame_1 92
Spectrum Identification Results
Validated
SE | Rnk | Sequence | Charge | Confidence
1 1 NH2-MTYCHTDVSHFEIQQLNYL-COOH 3 95
2 2 NH2-YLPQVEIVSNLPEFDNGGCEK-COOH 3 79
3 3 NH2-QLPYDWSEIIEWFWCVAR-COOH 3 51
4 4 NH2-GVPDAIINLDDVLINKPKMCR-COOH 3 37
5 5 NH2-MGVIFGTGVNGAYYDVCSDIEK-COOH 3 30
OMSSA PeptideShaker
Spectrum & Fragment Ions (NH2-MTYC<cmm>HTDVSHFEIQQLNYL-COOH 3 800.52 m/z)
Int
m/z
Ions | Other | Loss | Charge | De Novo | Settings | Export | Help
Spectrum Identification Overview
4,251 4,267 #PSMs
4,251 16 #Unique PSMs
15,214 15,198 #Unassigned
21.8 21.9 ID Rate (%)

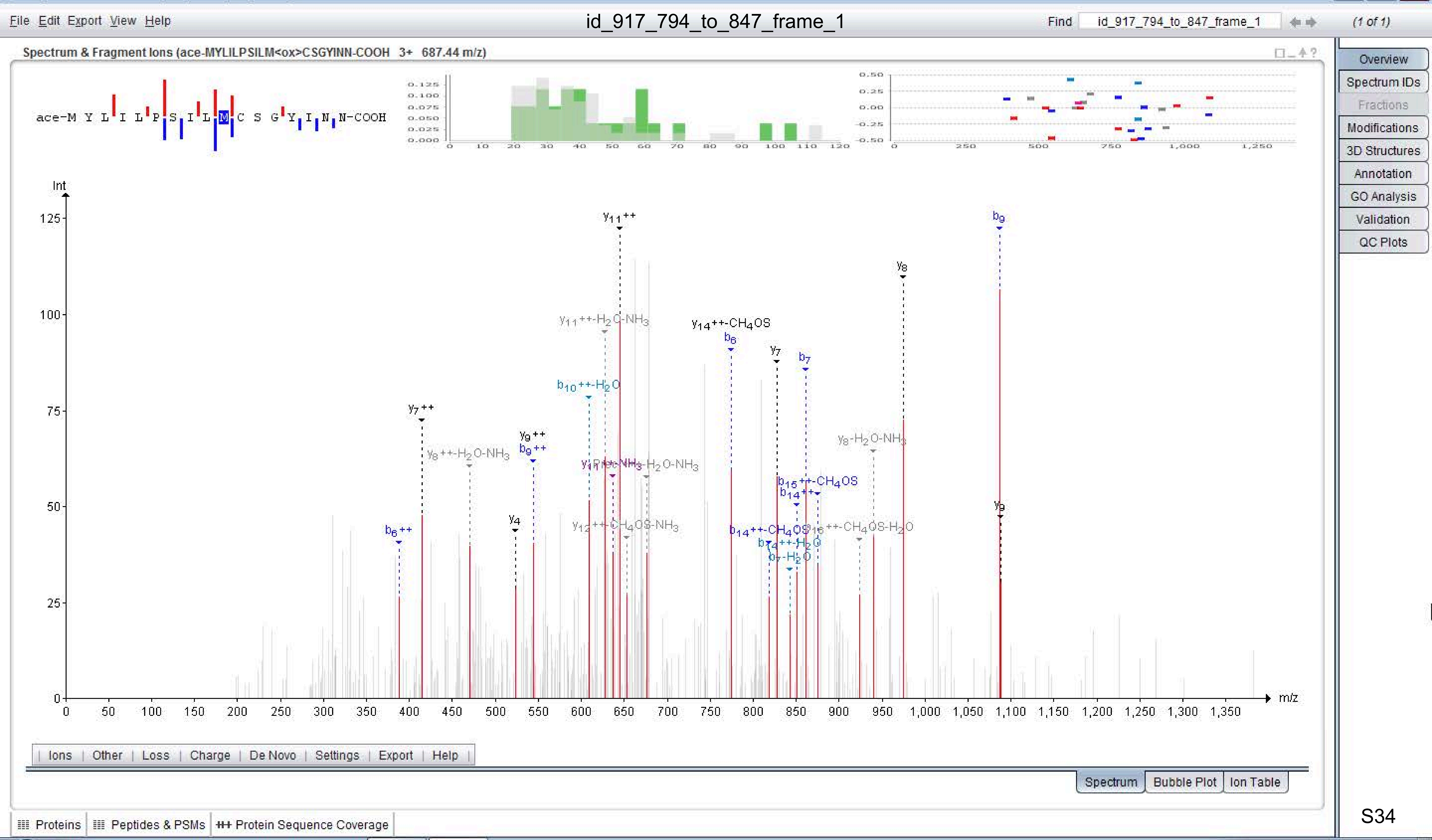
File Edit Export View Help
id_917_794_to_847_frame_1
Find id_917_794_to_847_frame_1
(1 of 1)
Spectrum & Fragment Ions (ace-MYLILPSILM<ox>CSGYINN-COOH 3+ 687.44 m/z)
ace-M Y L I L P S I L M C S G Y I N N-COOH
Overview
Spectrum IDs
Fractions
Modifications
3D Structures
Annotation
GO Analysis
Validation
QC Plots
Int
m/z
Ions | Other | Loss | Charge | De Novo | Settings | Export | Help
Spectrum
Bubble Plot
Ion Table
Proteins
Peptides & PSMs
Protein Sequence Coverage

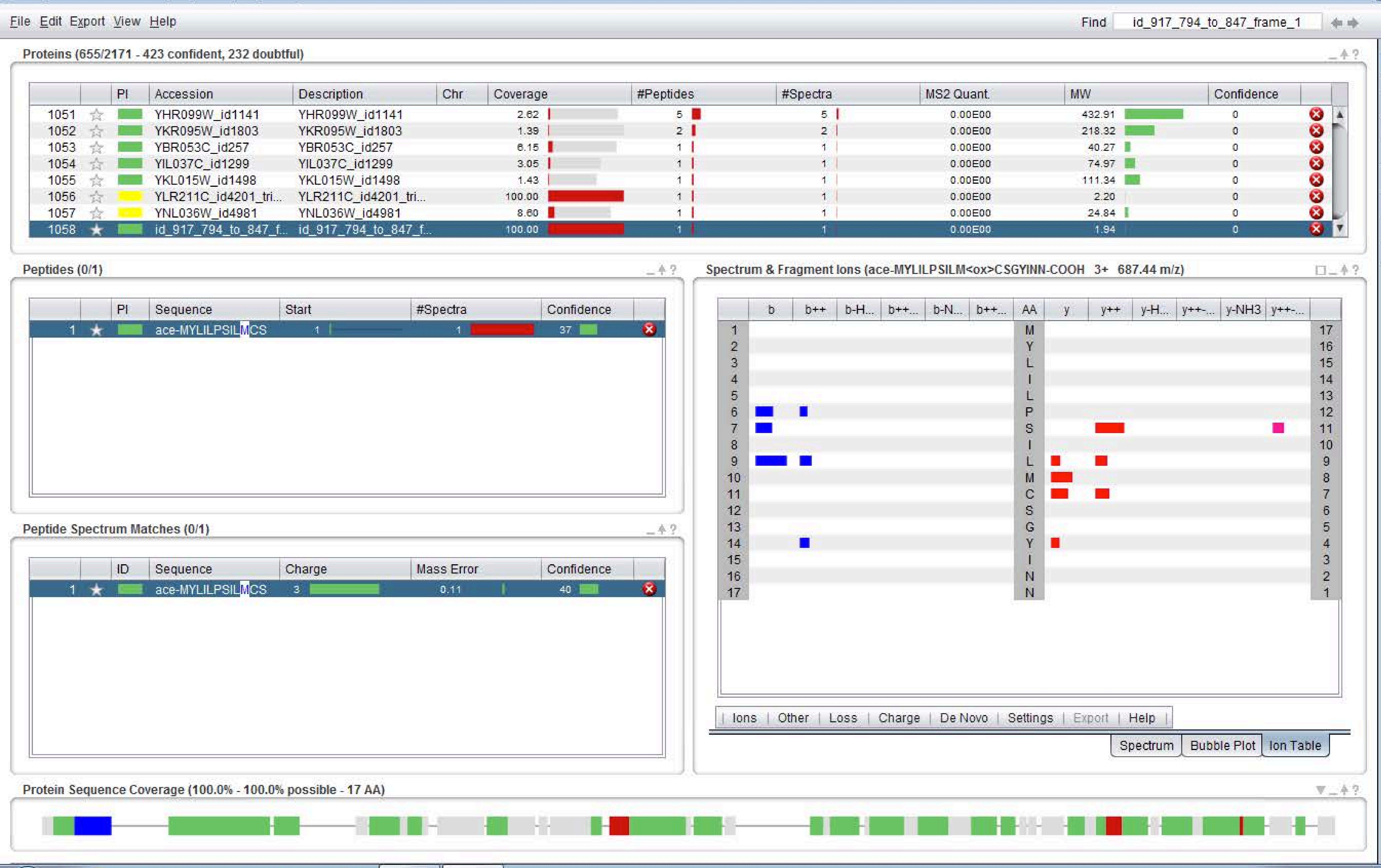
File Edit Export View Help
Find id_917_794_to_847_frame_1 (1 of 1)
Overview
Spectrum IDs
Fractions
Modifications
3D Structures
Annotation
GO Analysis
Validation
QC Plots
Proteins (655/2171 - 423 confident, 232 doubtful)
PI Accession Description Chr Coverage #Peptides #Spectra MS2 Quant. MW Confidence
1051 YHR099W_id1141 YHR099W_id1141 2.62 5 5 0.00E00 432.91 0
1052 YKR095W_id1803 YKR095W_id1803 1.39 2 2 0.00E00 218.32 0
1053 YBR053C_id257 YBR053C_id257 6.15 1 1 0.00E00 40.27 0
1054 YIL037C_id1299 YIL037C_id1299 3.05 1 1 0.00E00 74.97 0
1055 YKL015W_id1498 YKL015W_id1498 1.43 1 1 0.00E00 111.34 0
1056 YLR211C_id4201_tri... YLR211C_id4201_tri... 100.00 1 1 0.00E00 2.20 0
1057 YNL036W_id4981 YNL036W_id4981 8.60 1 1 0.00E00 24.84 0
1058 id_917_794_to_847_f... id_917_794_to_847_f... 100.00 1 1 0.00E00 1.94 0
Peptides (0/1)
PI Sequence Start #Spectra Confidence
1 ace-MYLILPSILMCS 1 1 37
Peptide Spectrum Matches (0/1)
ID Sequence Charge Mass Error Confidence
1 ace-MYLILPSILMCS 3 0.11 40
Spectrum & Fragment Ions (ace-MYLILPSILM<ox>CSGYINN-COOH 3+ 687.44 m/z)
b b++ b-H... b++... b-N... b++... AA y y++ y-H... y++-... y-NH3 y++-...
1 M 17
2 Y 16
3 L 15
4 I 14
5 L 13
6 P 12
7 S 11
8 I 10
9 L 9
10 M 8
11 C 7
12 S 6
13 G 5
14 Y 4
15 I 3
16 N 2
17 N 1
Ions Other Loss Charge De Novo Settings Export Help
Spectrum Bubble Plot Ion Table
Protein Sequence Coverage (100.0% - 100.0% possible - 17 AA)

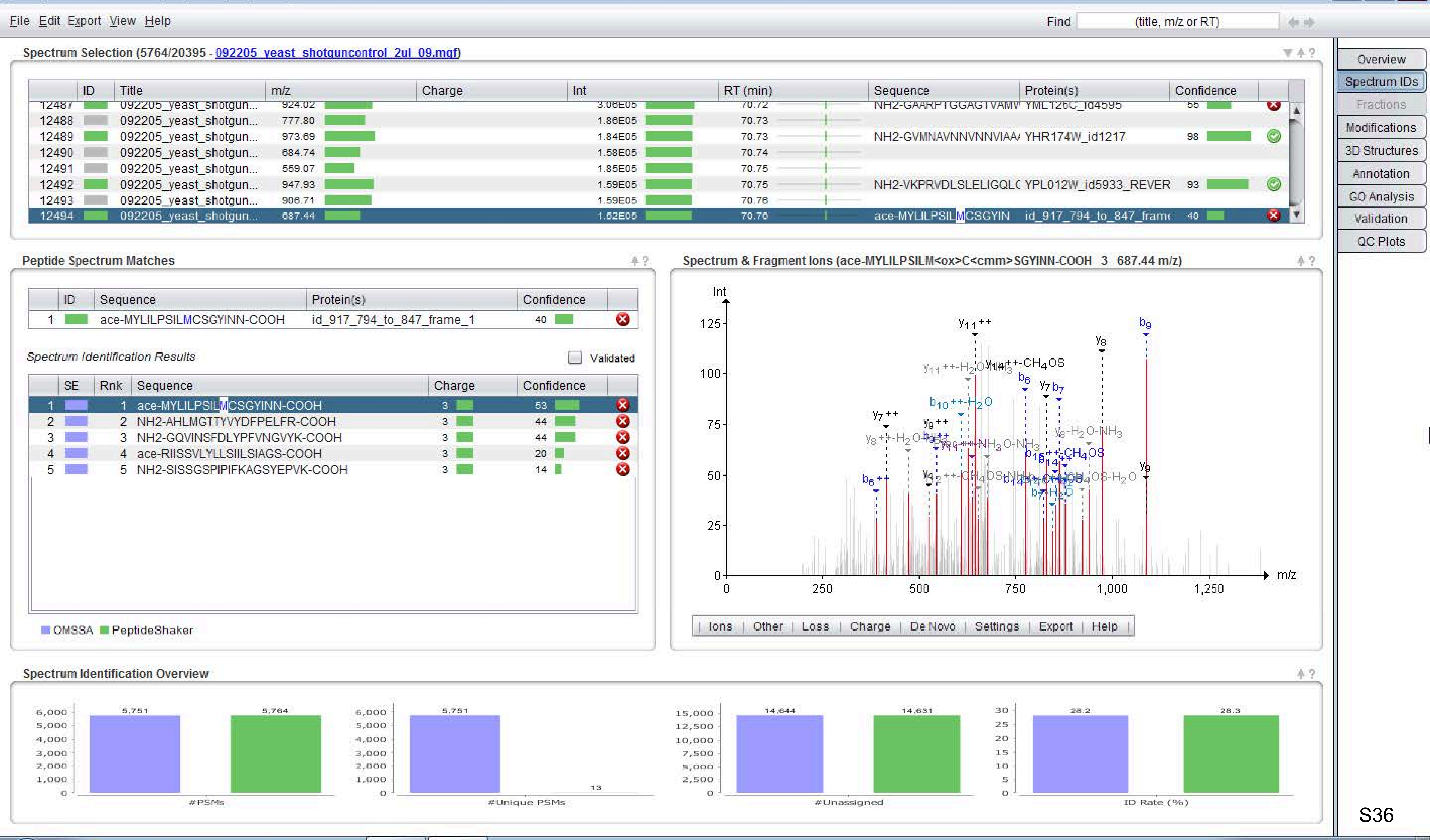
File Edit Export View Help
Find (title, m/z or RT)
Spectrum Selection (5764/20395 - 092205_yeast_shotguncontrol_2ul_09.mgf)
ID Title m/z Charge Int RT (min) Sequence Protein(s) Confidence
12488 092205_yeast_shotgun... 777.80 1.86E05 70.73
12489 092205_yeast_shotgun... 973.69 1.84E05 70.73 NH2-GVMNAVNNVNNVIAA YHR174W_id1217 98
12490 092205_yeast_shotgun... 684.74 1.58E05 70.74
12491 092205_yeast_shotgun... 569.07 1.85E05 70.75
12492 092205_yeast_shotgun... 947.93 1.59E05 70.75 NH2-VKPRVDLSLELIGQLC YPL012W_id5933_REVER 93
12493 092205_yeast_shotgun... 906.71 1.59E05 70.76
12494 092205_yeast_shotgun... 687.44 1.52E05 70.76 ace-MYLILPSILMCSGYIN id_917_794_to_847_frame 40
Overview
Spectrum IDs
Fractions
Modifications
3D Structures
Annotation
GO Analysis
Validation
QC Plots
Peptide Spectrum Matches
ID Sequence Protein(s) Confidence
1 ace-MYLILPSILMCSGYINN-COOH id_917_794_to_847_frame_1 40
Spectrum Identification Results
Validated
SE Rnk Sequence Charge Confidence
1 1 ace-MYLILPSILMCSGYINN-COOH 3 53
2 2 NH2-AHLMGTTYVYDFPELFR-COOH 3 44
3 3 NH2-GQVINSFDLYPFVNGVYK-COOH 3 44
4 4 ace-RIISSVLYLLSIILSIAGS-COOH 3 20
5 5 NH2-SISSGSPIPIFKAGSYEPVK-COOH 3 14
OMSSA PeptideShaker
Spectrum & Fragment Ions (ace-MYLILPSILM<ox>C<cmm>SGYINN-COOH 3 687.44 m/z)
Int
m/z
Ions Other Loss Charge De Novo Settings Export Help
Spectrum Identification Overview
5,751 5,764
#PSMs
5,751 13
#Unique PSMs
14,644 14,631
#Unassigned
28.2 28.3
ID Rate (%)
